# Genomic analysis of the nomenclatural type strain of the nematode-associated entomopathogenic bacterium *Providencia vermicola*

**DOI:** 10.1186/s12864-021-08027-w

**Published:** 2021-10-02

**Authors:** Giuseppe Andolfo, Christina Schuster, Haifa Ben Gharsa, Michelina Ruocco, Andreas Leclerque

**Affiliations:** 1 Department of Agricultural Sciences, University of Naples “Federico II”, Via Università 100, 80055 Portici, Italy; 2 Department of Biology, Technische Universität Darmstadt, Schnittspahnstraße 10, 64287 Darmstadt, Germany; 3 Istituto per la Protezione Sostenibile delle Piante (IPSP), Consiglio Nazionale delle Ricerche (CNR), Piazzale Enrico Fermi 1, 80055 Portici, Italy

**Keywords:** *Providencia vermicola*, Whole genome analysis, Phylogenomics, Entomopathogenicity, Nematode association, Antibiotic resistance, Type III secretion system (T3SS), Biological control

## Abstract

**Background:**

Enterobacteria of the genus *Providencia* are mainly known as opportunistic human pathogens but have been isolated from highly diverse natural environments. The species *Providencia vermicola* comprises insect pathogenic bacteria carried by entomoparasitic nematodes and is investigated as a possible insect biocontrol agent. The recent publication of several genome sequences from bacteria assigned to this species has given rise to inconsistent preliminary results.

**Results:**

The genome of the nematode-derived *P. vermicola* type strain DSM_17385 has been assembled into a 4.2 Mb sequence comprising 5 scaffolds and 13 contigs. A total of 3969 protein-encoding genes were identified. Multilocus sequence typing with different marker sets revealed that none of the previously published presumed *P. vermicola* genomes represents this taxonomic species. Comparative genomic analysis has confirmed a close phylogenetic relationship of *P. vermicola* to the *P. rettgeri* species complex. *P. vermicola* DSM_17385 carries a type III secretion system (T3SS-1) with probable function in host cell invasion or intracellular survival. Potentially antibiotic resistance-associated genes comprising numerous efflux pumps and point-mutated house-keeping genes, have been identified across the *P. vermicola* genome. A single small (3.7 kb) plasmid identified, pPVER1, structurally belongs to the *qnrD*-type family of fluoroquinolone resistance conferring plasmids that is prominent in *Providencia* and *Proteus* bacteria, but lacks the *qnrD* resistance gene.

**Conclusions:**

The sequence reported represents the first well-supported published genome for the taxonomic species *P. vermicola* to be used as reference in further comparative genomics studies on *Providencia* bacteria. Due to a striking difference in the type of injectisome encoded by the respective genomes, *P. vermicola* might operate a fundamentally different mechanism of entomopathogenicity when compared to insect-pathogenic *Providencia sneebia* or *Providencia burhodogranariea*. The complete absence of antibiotic resistance gene carrying plasmids or mobile genetic elements as those causing multi drug resistance phenomena in clinical *Providencia* strains, is consistent with the invertebrate pathogen *P. vermicola* being in its natural environment efficiently excluded from the propagation routes of multidrug resistance (MDR) carrying genetic elements operating between human pathogens. Susceptibility to MDR plasmid acquisition will likely become a major criterion in the evaluation of *P. vermicola* for potential applications in biological pest control.

**Supplementary Information:**

The online version contains supplementary material available at 10.1186/s12864-021-08027-w.

## Background

Gamma-proteobacteria of the genus *Providencia* (Enterobacterales; Morganellaceae) have been identified in diverse environmental samples as well as in association with both vertebrate and invertebrate animals and humans. Six out of 10 currently recognized *Providencia* species, namely *P. rettgeri, P. stuartii, P. alcalifaciens, P. rustigianii, P. heimbachae* and *P. huaxiensis* [1], were isolated from clinical samples [2–4] and comprise opportunistic human pathogens typically causing diarrhea [5–7] and urinary tract infections [8] that are often clinically complicated by multidrug resistance of the pathogen [9–12]. Moreover, bacteria assigned to the recognized *Providencia* species *P. vermicola, P. rettgeri, P. alcalifaciens, P. sneebia,* and *P. burhodogranariaea* [1] together with the recently proposed new species *P. entomophila* [13] have been found associated with or pathogenic to insects as honeybees [14], house and blow flies [15, 16], the fly-parasitic wasp *Nasonia vitripennis* [17], and a diverse range of fruit flies [13, 18–23].

The species *P. vermicola* is conspicuous among these as the putative insecticidal agent carried by entomoparasitic nematodes of the genera *Steinernema* [24], *Butlerius* [25] or *Rhabditis* [26]. However, further nematode-associated bacterial entomopathogens were identified as *Providencia* sp. [27] or *P. rettgeri* [28, 29]. The host-vector-pathogen relationship of these *Providencia* strains is functionally reminiscent of closely related *Xenorhabdus* and *Photorhabdus* bacteria (Enterobacterales; Morganellaceae) [30, 31]. A possible contribution of nematode-associated *Providencia* bacteria to insect biocontrol has been evaluated [25, 28, 29].

Moreover, *P. vermicola* has been reported to be a fish pathogen [32–34]. Several *Providencia* species, including *P. vermicola,* [35, 36] have been described as remarkably resistant to high concentrations of metals [37–40] and to several types of antibiotics [35, 36, 41]. During the past decade, a multi drug resistance (MDR) phenomenon linked to the spread of integrons carrying antibiotic resistance genes including the New Delhi metallo-lactamase gene (*ndm1)* has gained clinical importance globally. The *ndm1* encoded carbapenemase enables pathogenic enterobacteria, including *P. rettgeri* and *P. stuartii*, to hydrolyze a wide spectrum of ß-lactam antibiotics [41, 42].

An increasing number of *Providencia* genome sequences have been published during the past decade, including the recent publication of the genomes (i.e. assemblies GCA_014396895.1, GCA_010748935.1 and GCA_016618195.1) of three *Providencia* strains assigned to the species *P. vermicola,* namely strain G1 isolated from fish in Algeria, strain P8538 obtained from a clinical sample in Congo and strain LLDRA6 isolated from contaminated soil in China [43]. Comparative approaches have been used to explore the genomes of both clinical [44] and insect derived [45] *Providencia* bacteria. However, the species *P. vermicola* has not been covered by these studies, mainly due to the lack of a reliable reference genome.

This study reports the genome sequence of the nomenclatural type strain *Providencia vermicola* DSM_17385 and presents the first complete genome analysis for the taxonomic species *P. vermicola*. Strain DSM_17385 has previously been isolated from infective juveniles of the entomoparasitic nematode *Steinernema thermophilum* collected in soils at New Delhi, India, and has been recognized as the type strain of a new taxonomic species on the basis of 16S rRNA gene comparisons, restriction pattern based ribo-printing, metabolic property analyses, and a DNA-DNA relatedness value of 30% with respect to the *P. rettgeri* type strain as determined by physical DNA-DNA reassociation [24].

## Results

### General characteristics of the *P. vermicola* DSM_17385 genome

**Fig. 1:**
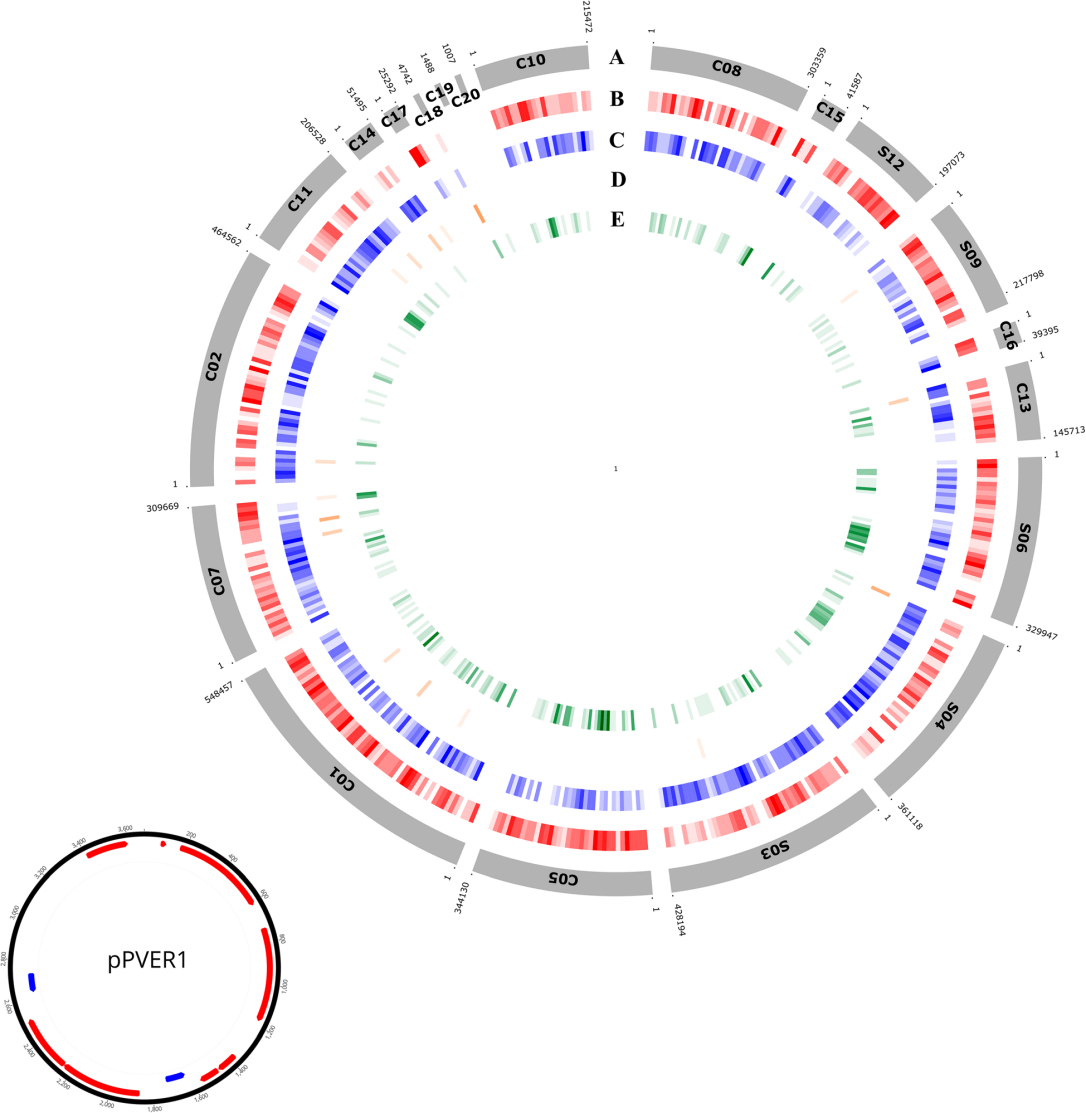
Circular maps of the *P. vermicola* DSM_17385 genome (**A**) and of plasmid pPVER1 (**B**). **A** The scaffolds and contigs are ordered and oriented for maximum synteny with the *P. rettgeri* Dmel1 genome sequence. Scaffolds (S) and contigs (**C**) are not positioned in a complete circle because their order and orientation are not empirically known. The size of the gaps between scaffolds is unknown. Rings from the outermost to the center: genes on the forward strand (red), genes on the reverse strand (blue), tRNA and rRNA genes (orange), genes unique to *P. vermicola* (green). **B** Physical map of plasmid pPVER1: ORFs on the forward strand (red), genes on the reverse strand (blue)

The genome of the *P. vermicola* type strain DSM_17385 was sequenced in this study and assembled into 5 scaffolds and 13 contigs, starting from 765,178 paired-end reads (MiSeq 2 × 250 bp). Basic genome information is given in Table 1. The accumulated scaffold and contig length of this assembly is 4.23 Mb (Fig. 1). The GC content, N50, and L50 of the DSM_17385 genome were 41.1%, 344,020 and 5, respectively. Annotation with GLIMMER resulted in 3969 protein-encoding genes, 220 (i.e. app. 6%) hypothetical proteins and 74 structural RNA encoding genes, more exactly three 5S rRNA, one 16S rRNA, one 23S rRNA, and 69 tRNA genes. As sequence gaps can cause interruption or deletion of ORFs, and as the DSM_17385 genome was not optically mapped, inferred annotations might be incomplete.

**Table 1:** Genomic features of *P. vermicola* strain DSM_17385

Attribute	Value
Sequencing platform	Illumina MiSeq
Assembler	SPADES
Assembly accession	JAGSPI000000000
Topology	Circular
No. of scaffolds/contigs	18
Genome size (bp)	4,233,718
DNA G + C (%)	41.1
Genome coverage (X)	30
Number of RNAs genes	74
Number of tRNAs genes	69
N50	344,819
L50	5
Number of CDSs	3969
Pseudo genes	220

**Fig. 2:**
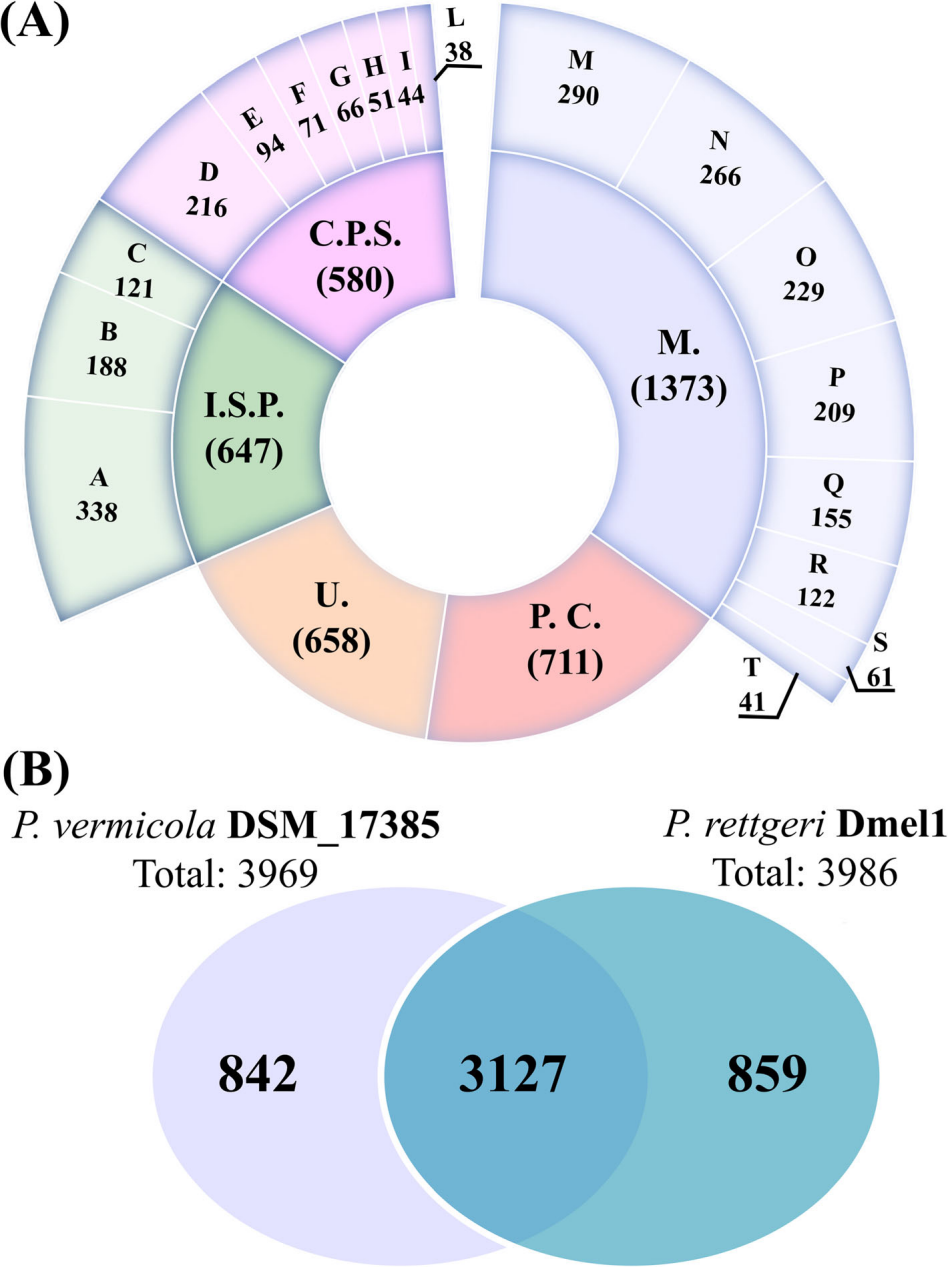
Functional annotation of *P. vermicola* DSM_17385 genes. **A** COG categories of predicted *P. vermicola* genes on the inner and COG subcategories on the outer ring. Each category or subcategory is graphed as a percentage of the total number of genes. M.: metabolism; P.C.: poorly characterized; U.: uncharacterized; I.S.P.: information storage and processing; C.P.S.: cellular processes and signaling. **B** Numbers of shared and unique protein-encoding genes when comparing *P. vermicola* DSM_17385 to *P. rettgeri* Dmel1. Numbers are the gene counts within each sector of the Venn diagram

We performed a GO-term annotation analysis of all protein-encoding genes identified by GLIMMER. Through this analysis, we were able to assign functional annotations to 3749 (app. 95%) of the predicted genes (Fig. 2a). Coding sequences were assigned to putative super-functional and functional categories using the clusters of orthologous groups of proteins (COG) database [46]. Approximately 40% of the predicted genes were dedicated to metabolic functions. One-third was roughly evenly split between cellular process/signaling functions and information storage/processing functions. Functions of those predicted genes in the remaining 20% of genome were either poorly categorized or uncategorized (Fig. 2a).

### Multilocus sequence typing and phylogenetic reconstruction

Phylogenetic reconstruction from three independent datasets was performed in order to assess the systematic position of *P. vermicola* DSM_17385. The complete 16S ribosomal RNA encoding gene comprising in length 1528 nucleotides was employed at a first level of phylogenetic analysis, extending the previous 16S rRNA based phylogenetic analysis [24] to a larger set of reference strains. The second dataset, referred to as “hMLST”, was composed of five housekeeping genes encoding translation elongation factor EF-G (*fusA),* DNA gyrase subunit B *(gyrB),* isoleucyl-tRNA synthetase *(ileS),* translation elongation factor EF-4 (*lepA)*, and leucyl-tRNA synthetase *(leuS)* that have been used previously in molecular taxonomy studies of *Providencia* bacteria [13, 19]. The third dataset, referred to as “rMLST”, comprised the full set of ribosomal *rpl, rpm* and *rps* proteins employed in bacterial rMLST approaches [47]. The full coding sequences of the 16S rRNA, the five hMLST and 53 rMLST markers were identified in the genome sequence under study (Additional files 1 and 2).

Comparison of the 16S rRNA genes from a set of 31 bacterial strains representing the major groups of *Providencia* bacteria including the nomenclatural type strains of all recognized *Providencia* species gave rise to a phylogenetic tree (Suppl. Figure 1) generally characterized by insufficiently bootstrap supported clades. *P. vermicola* DSM_17385 together with supposed *P. vermicola* strain G1 and several strains assigned to the species *P. rettgeri* were grouped together in a clade with branches receiving from 20 to 52% bootstrap support. The two further supposed *P. vermicola* strains P8358 and LLDRA6 together with the *P. sneebia* type strain were loosely, i.e. with bootstrap support values between 16 and 43%, associated to an optimally supported clade comprising all *P. stuartii* strains together with the *P. thailandensis* type strain. Insufficient resolution of 16S rRNA phylogenies at the level of *Providencia* species delineation had already been stated previously [19, 24].

**Fig. 3:**
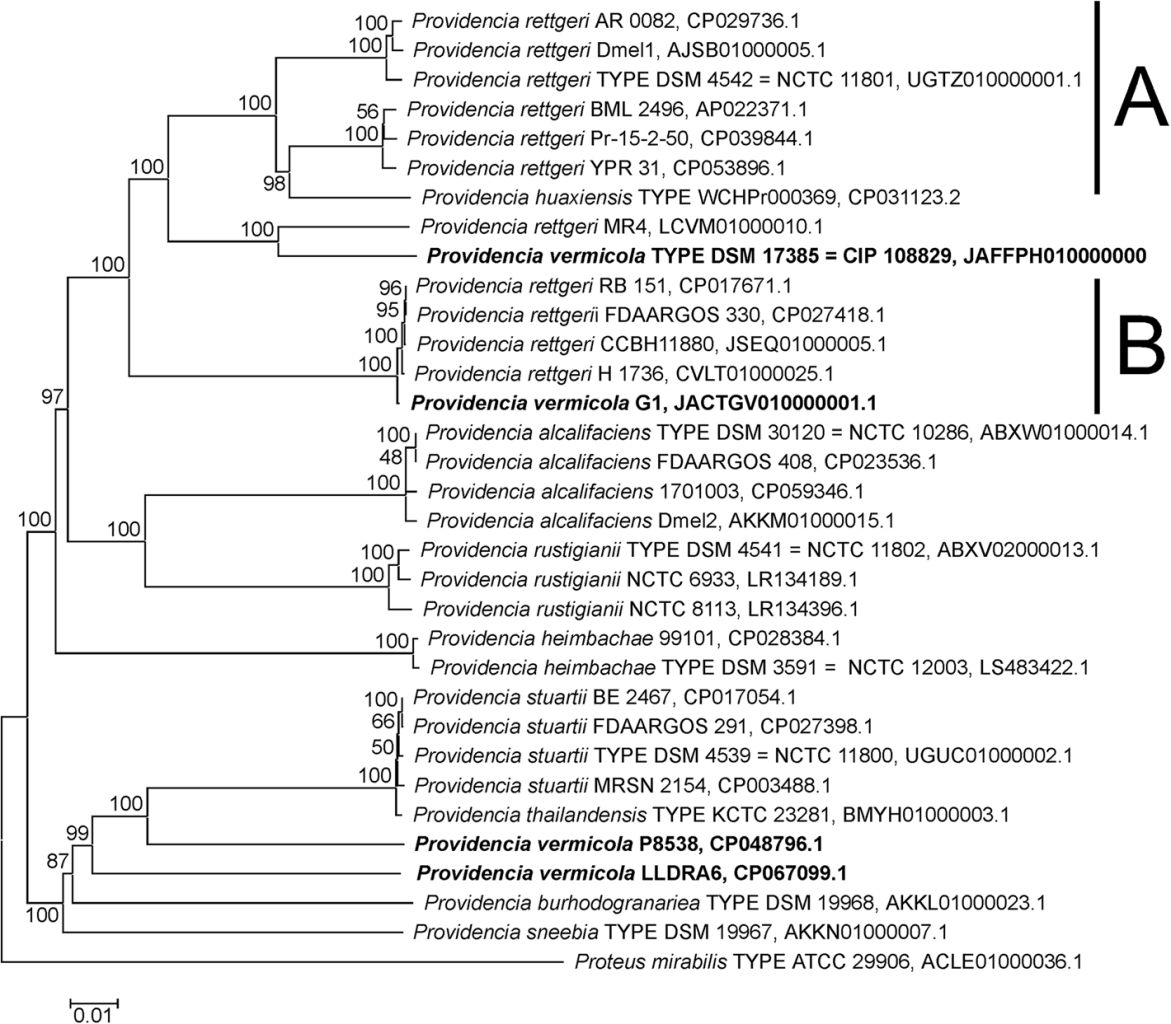
Neighbor Joining (NJ) phylogeny of *Providencia* bacteria as reconstructed from concatenated complete *fusA, gyrB, ileS, lepA* and *leuS* gene sequences. Terminal branches are labelled by genus, species and strain designations as well as GenBank accession numbers; “TYPE” indicates nomenclatural type strains of the respective taxonomic species. Bacterial strains that have been assigned to the species *P. vermicola* are in bold type. Numbers on branches indicate bootstrap support percentages. *P. rettgeri* clades A and B are indicated at the right margin. The size bar corresponds to 1% sequence divergence; the length of dashed lines is not true to scale. The concatenation of orthologous sequences from the closely related bacterium *Proteus mirabilis* has been used as outgroup

**Fig. 4:**
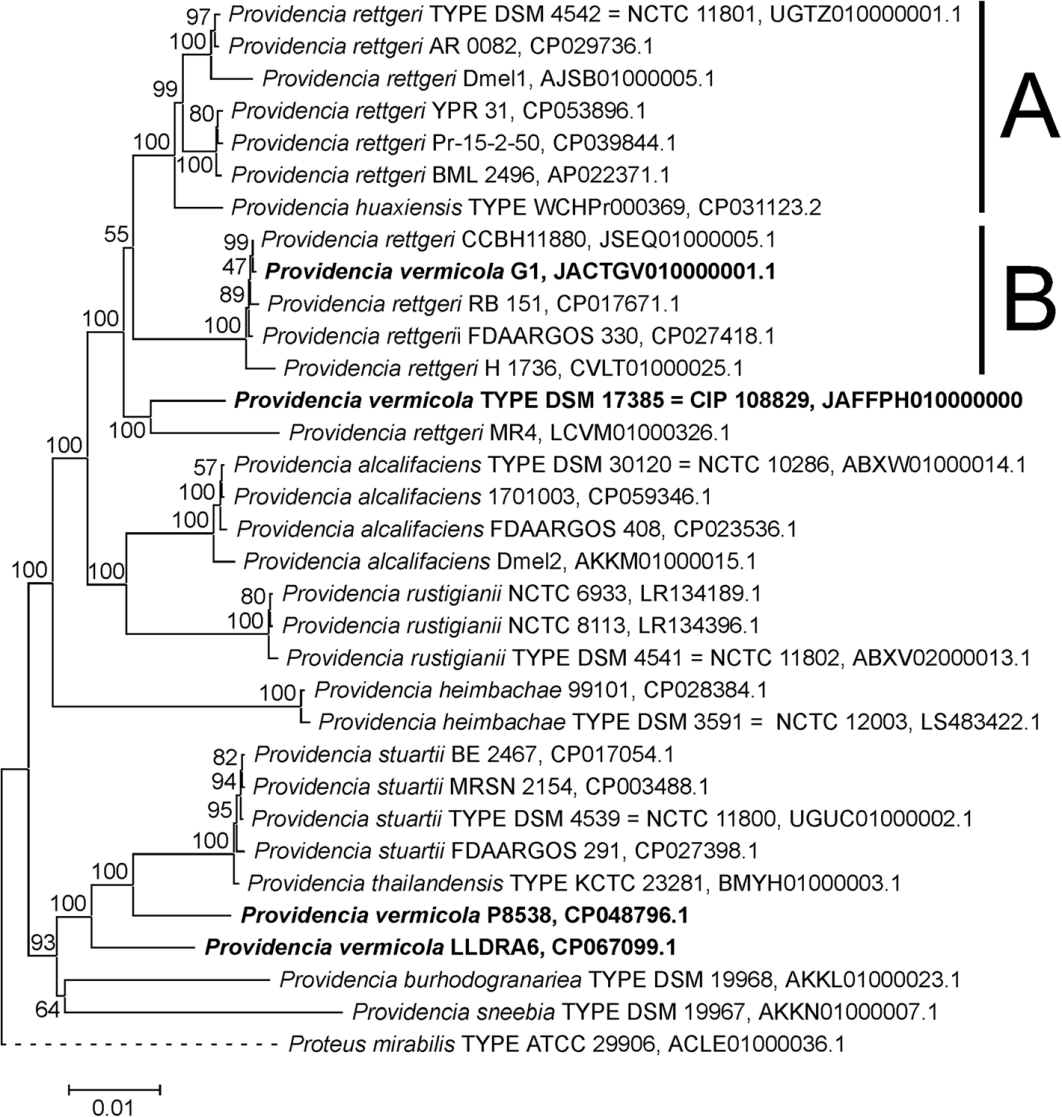
Neighbor Joining (NJ) phylogeny of *Providencia* bacteria as reconstructed from 53 concatenated ribosomal protein encoding genes. Terminal branches are labelled by genus, species and strain designations as well as GenBank accession numbers; “TYPE” indicates nomenclatural type strains of the respective taxonomic species. Bacterial strains that have been assigned to the species *P. vermicola* are in bold type. Numbers on branches indicate bootstrap support percentages. *P. rettgeri* clades A and B are indicated at the right margin. The size bar corresponds to 1% sequence divergence; the length of dashed lines is not true to scale. The concatenation of orthologous sequences from the closely related bacterium *Proteus mirabilis* has been used as outgroup

Concatenation of the identified hMLST and rMLST marker genes resulted in combined meta-gene sequences comprising 11,619 bp and 21,267 bp in length, respectively. Comparison with the concatenated orthologs from 31 representative *Providencia* genomes gave rise to phylogenies of essentially identical tree topology (Figs. 3 and 4). In particular, both the hMLST and rMLST based phylogenies coincided i) in placing the *P. vermicola* type strain DSM_17385 into a sister clade position to clades A and B of the *P. rettgeri* complex, well delineated from the type strains of all further *Providencia* species, and ii) in not co-locating the presumed *P. vermicola* whole genome sequences from *Providencia* strains P8538, LLDRA6 and G1 with the *P. vermicola* type strain. Whereas *Providencia* strain G1 was firmly, i.e. with 100% bootstrap support in both phylogenies, positioned within the *P. rettgeri* clade B, strains P8538 and LLDRA6 appeared loosely related to each other and to an optimally bootstrap supported clade comprising the nomenclatural type strains of both the species *P. stuartii* and *P. thailandensis*. Concerning the systematic position of *Providencia* strains P8358 and LLDRA6, both these hMLST and rMLST phylogenies essentially reproduced the results obtained from 16S rRNA gene comparisons.

When phylogenetic reconstruction from the concatenated hMLST marker set was extended to all 195 *Providencia* genomes currently available in the Genbank database (Suppl. Figure S2), both strains P8358 and LLDRA6 formed 100% bootstrap supported clades with a small number of strains assigned to the species *P. stuartii*. However, these clades were well delineated from the “main” *P. stuartii* clade comprising at 100% bootstrap support the vast majority of all strains assigned to this species together with the nomenclatural type strains of both *P. stuartii* and *P. thailandensis*.

Ribosomal typing of *P. vermicola* DSM_17385 and the three presumed *P. vermicola* genome strains gave rise to highly diverse rMLST based taxonomic assignments (Table 2). When compared to the PubMLST ribosomal protein encoding gene database, exactly matching alleles were identified for only five out of the 53 rMLST marker genes identified in the *P. vermicola* type strain genome, providing a too scarce statistical basis for reliable ribosomal sequence type (rST) assignment. The rMLST based taxonomic assignment to either *P. rettgeri* or *P. burhodogranariea* remained ill-supported and inconclusive. Ribosomal typing of *Providencia* strain P8538, in contrast, resulted in an apparently highly conclusive outcome, with 53/53 exact allele matches giving rise to an unequivocal rST identification and a maximally supported taxonomic assignment to the species *P. vermicola*. However, as the P8538 genome currently serves as reference for the species *P. vermicola* in the PubMLST database, these results cannot be judged meaningful. On the basis of ribosomal typing, presumed *P. vermicola* strains LLDRA6 and G1 were assigned with low to intermediate support to the species *P. stuartii* and *P. rettgeri* (clade B), respectively (Table 2).

**Table 2:** rMLST results for presumed *P. vermicola* genome sequencing strains

Strain designation	Type strain	Exact allele matches	Number of rST assigned (Mismatch threshold appl.)	rMLST based assignment (support level)
DSM_17385	Yes	5/53	0 (n.a.)	*P. rettgeri* (75%) *P. burhodogranariea* (25%)
G1	No	41/52	12 (20)	*P. rettgeri* (92%)
LLDRA6	No	53/53	3 (20)	*P. stuartii* (79%)
P8538	No	53/53	1 (1–5)	*P. vermicola* (100%)
MR4	No	49/50	3 (20)	*P. rettgeri* (100%)

Phylogenetic reconstruction based on the concatenated hMLST marker set from the 195 *Providencia* genomes available in the Genbank database demonstrated that *P. vermicola* DSM_17385 appears molecular taxonomically most closely related to a single genome strain, namely *Providencia* strain MR4 that has previously been assigned to the species *P. rettgeri* (Suppl. Figure S2). In both the concatenated hMLST and rMLST marker based phylogenies *P. vermicola* DSM_17385 and strain MR4 form a maximally bootstrap supported clade with comparatively long branches indicating considerable sequence divergence (Figs. 3 and 4). However, orthologs of only 4/5 hMLST markers (all but gene *lepA*) and 50/53 rMLST markers (all but genes *rplM, rpsI* and *rpsL*) were identified in the published MR4 genome sequence. With respect to the hMLST data set, the respective clade was supported in the *gyrB, ileS* and *leuS*, but not in the the *fusA* single marker phylogenies. With respect to the rMLST data set, the p-distance matrix based pair-wise sequence similarity for the concatenation of 50 rMLST marker alleles from strains DSM_17385 and MR4 was calculated to be 97.8%. This corresponded to the pair-wise sequence similarities of the *P. vermicola* type strain to the *P. rettgeri* (clade A) type strain DSM_4542 (97.9%), the *P. huaxiensis* type strain WCHPr000369 (97.8%) or strains making up *P. rettgeri* clade B (range 97.4 - 97.5%) with sequence similarities to the type strains representing further *Providencia* species being 97.0% (*P. alcalifaciens*) or considerably lower (Suppl. Table S1). In contrast, sequence similarities within *P. rettgeri* clades A and B were generally superior to 99%. However, analogous pairwise sequence similarity percentages calculated from the hMLST data set are consistent with a comparatively closer phylogenetic relationship of *P. vermicola* DSM_17385 and strain MR4 (Suppl. Table S1). Ribosomal typing of *Providencia* strain MR4 identified 49/50 exact matches and gave rise to a maximally supported taxonomic assignment to the species *P. rettgeri* and to three rSTs representing this species (Table 2).

### Digital DNA-DNA hybridization analysis

**Fig. 5:**
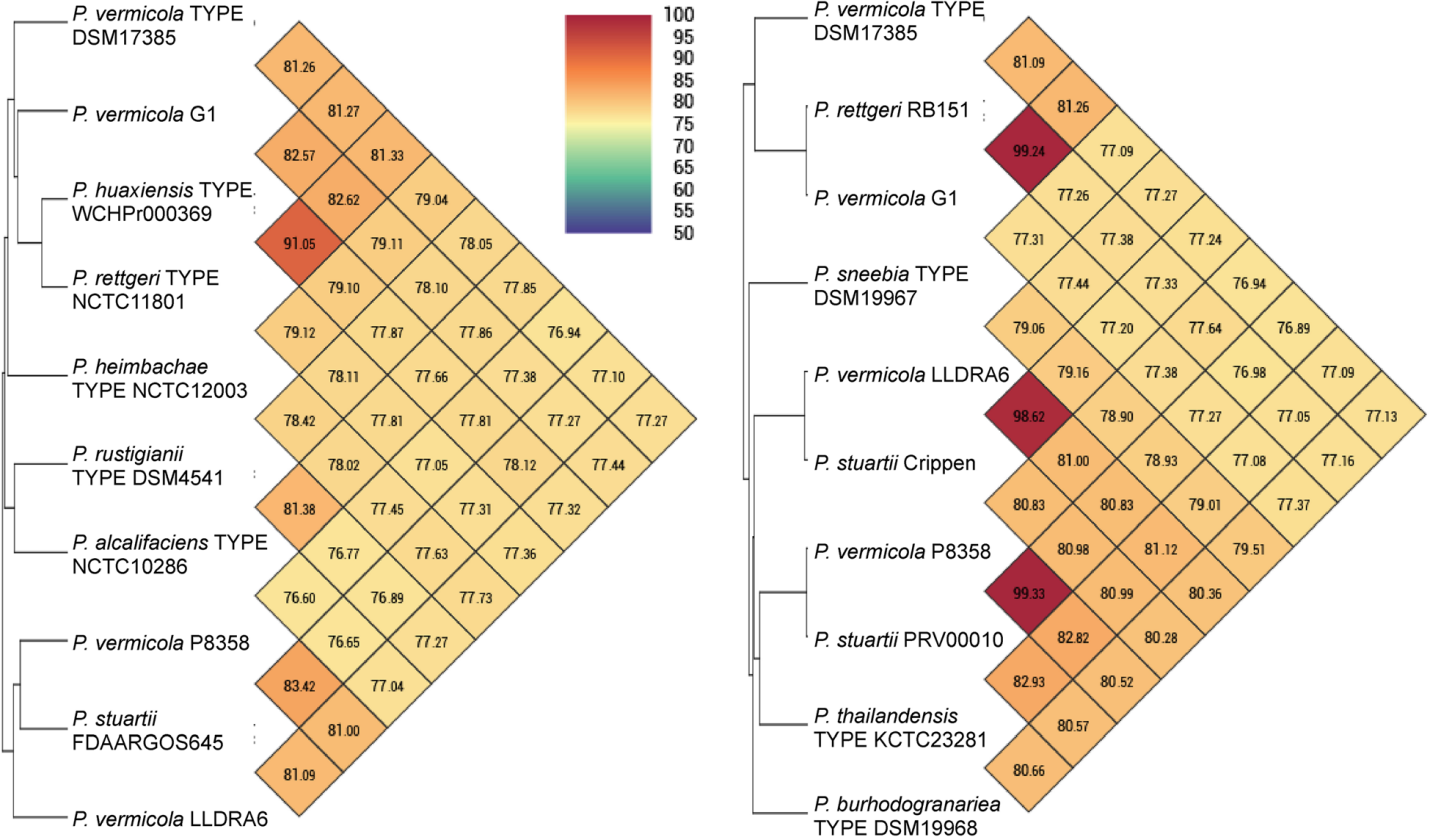
Heatmaps showing pair-wise average nucleotide identity by orthology (OrthoANI) percentages for two sets of *Providencia* genomes as calculated using the OAT software. *Providencia* strains are labelled by species and strain designations; “TYPE” indicates nomenclatural type strains of the respective taxonomic species

Among the methods for evolutionary distance assessment between bacterial species based on digital whole genome comparison, average nucleotide identity (ANI) is one of the most powerful approaches [48]. Therefore, pair-wise genome-wide average nucleotide identity by orthology (OrthoANI) has been determined for two subsets of 10 *Providencia* strains. The main results obtained were as follows (Fig. 5): i) pair-wise ANI values for the *P. vermicola* DSM_17385 genome and one of the nine *Providencia* type strains representing one of the further recognized species ranged between 77 and 81%, i.e. were in the range of values normally found for pair-wise ANI percentages across this set of specific type strains; ii) pair-wise ANI values for comparisons of strain DSM_17385 with one of the three further supposed *P. vermicola* strains G1, P8538 and LLDRA6 were in this same range, more exactly 81% for strain G1 and 77% for both strains P8538 and LLDRA6; iii) pair-wise ANI values for comparisons of supposed *P. vermicola* strains G1, P8538 and LLDRA6 and *Providencia* strains assigned to other species were found to be considerably higher than the above percentage range as, for instance, in the order of magnitude of 99% for the pair-wise comparisons of strain G1 with *P. rettgeri* strain RB151 (representing *P. rettgeri* clade B), of strain P8358 with *P. stuartii* strain PRV00010, and of strain LLDRA6 with *P. stuartii* strain Crippen.

Under the assumption that ANI values of 95–96% indicate bacterial species boundaries [49], these results are consistent with the following statements: i) *P. vermicola* strain DSM_17385 is not more closely related to any of the other specific type strains recognized within the genus *Providencia* as the latter are among each other and is, therefore, correctly considered type strain of an independent species; ii) none of the three supposed *P. vermicola* strains G1, P8358 and LLDRA6 belongs to the same species as the *P. vermicola* type strain; iii) instead, *Providencia* strains G1, P8358 and LLDRA6 should at the species level be assigned to the same taxon as strains *P. rettgeri* RB151, *P. stuartii* PRV00010 and *P. stuartii* Crippen, respectively.

### Comparative genomic analysis

A comparative genomics approach was employed to identify orthologous proteins in *P. vermicola* DSM_17385 and the insect-derived *P. rettgeri (*clade A) strain Dmel1. Dmel1 has been originally isolated from wild-caught *Drosophila melanogaster* and has been demonstrated to be a fruit fly pathogen [20]. Moreover, the genome of strain Dmel1 is well annotated and has been compared to genomes of fruit fly associated *Providencia* strains falling under the species *P. alcalifaciens, P. sneebia*, and *P. burhodogranariea* [45].

Based on this comparison, 3127 bona fide orthologous pairs were identified (Fig. 2b) with the large majority being present as single copies. This core genome is 78% of the total genes identified in each genome alone, and the corresponding orthologous gene pairs were distributed over 86% of the *P. vermicola* and 83% of the *P. rettgeri* genome. The corresponding genes together covered an analyzed genomic region of 3.6 Mb in both *P. vermicola* and *P. rettgeri*. The absolute number of unique genes, i.e. those not assigned to any orthologous pair, was very similar in both genomes, with 847 and 859 unique genes being detected in *P. vermicola* and *P. rettgeri*, respectively (Fig. 2b). Thus, unique single-copy genes represent app. 21% of the total genome content for both species.

**Fig. 6:**
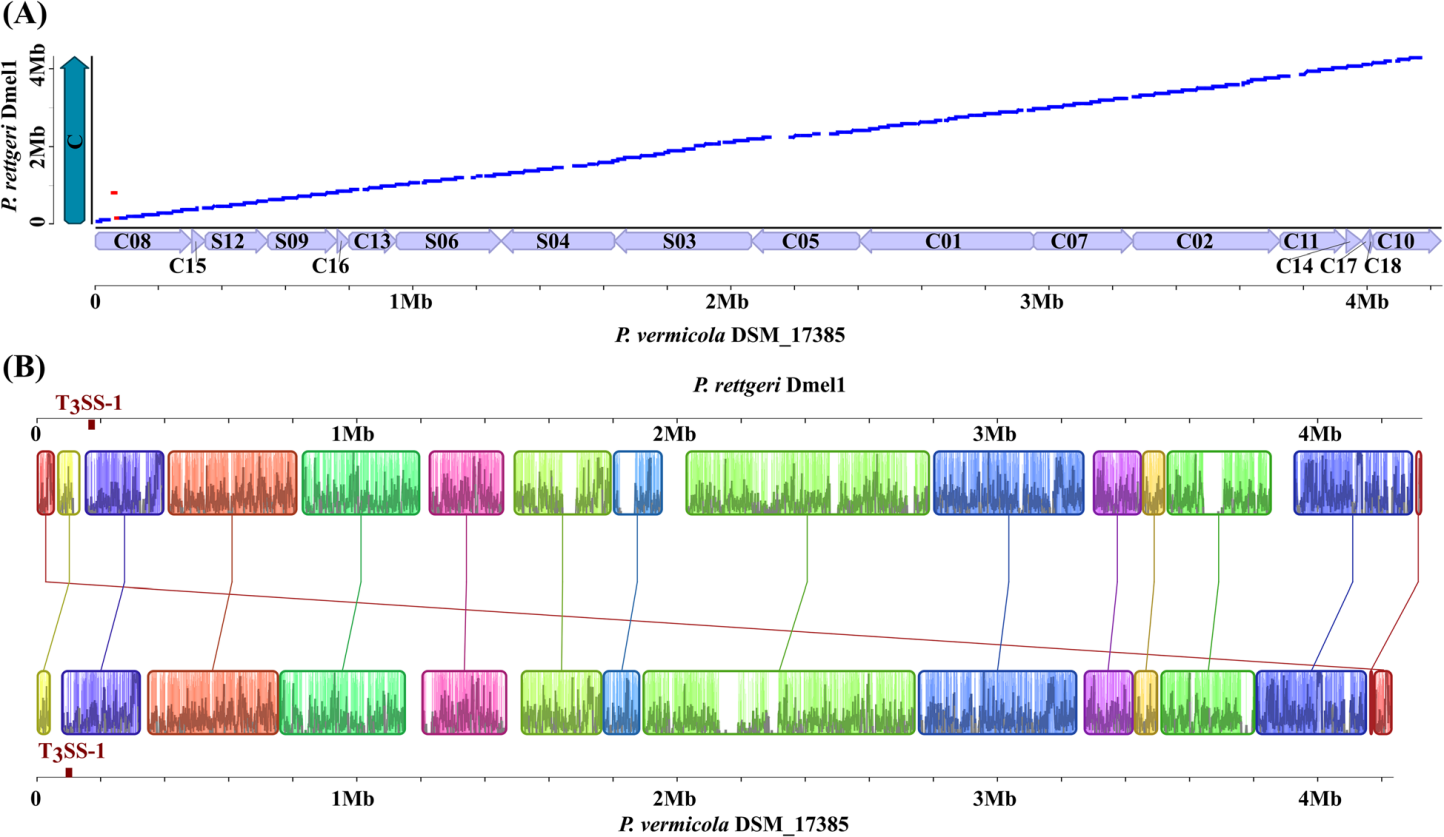
Comparison of strain *P. vermicola* DSM_17385 and *P. rettgeri* Dmel1 genome sequences. **A** Syntenic dotplot between *P. vermicola* DSM_17385 scaffolds or contigs (x-axis) and the *P. rettgeri* Dmel1 chromosome (y-axis). Syntenic regions were derived from orthologous blocks. Deflections of segments along either axis indicate insertions of DNA sequence. Blue dots represent homologous regions in the same, red dots in opposite orientation in both genome pairs. **B** Identification of collinear blocks in syntenic genomic regions. Collinear blocks are labelled with the same color and connected by lines. Block boundaries indicate breakpoints of genome rearrangements

The scaffolds and contigs of DSM_17385 were ordered and oriented so that they were as similar to the *P. rettgeri* Dmel1 genomic orientations as possible, assuming the most parsimonious evolution of genome arrangements. The global identity estimated from LASTZ was 82.3% (Fig. 6a). Genomic rearrangements are highlighted on the physical synteny map reported in Fig. 6b. It is in principle possible that any of the *P. vermicola* contigs could be inverted or rearranged relative to their positions on our comparative syntenic plot, but only if the rearrangement breakpoints lie at contig breakpoints.

The genomic rearrangements associated with speciation of *P. vermicola* and *P. rettgeri* from a common ancestor have partially preserved the location and organization of several homologous genomic regions. A total of 15 collinear blocks were discovered and identified between *P. vermicola* and *P. rettgeri* genomes (Fig. 6b). Many small rearrangements and two larger inversions were apparent across both genomes, the latter (in red Fig. 6a) involving a genomic region of app. 16 kb on contig 8. In particular, a large genomic inversion of app. 800 kb including a type III secretion system (T3SS-1) encoding gene cluster that has been identified previously in *P. sneebia* [45] is syntenically oriented in the *P. vermicola* genome with respect to *P. rettgeri* Dmel1.

### Type III secretion systems

**Fig. 7:**
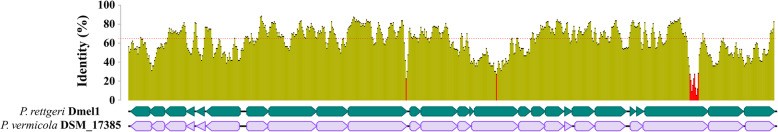
Alignment of type III secretion systems (T3SS-1) of *P. vermicola* DSM_17385 and *P. rettgeri* Dmel1. The graph shows the pairwise identity (sliding windows size of 100 nucleotide) between T3SS-1 sequences. Average pairwise identity across the full length sequence (64.5%) is indicated by the dashed red line. Colored arrows indicate individual genes and their orientation. The aligned genomic regions indicate the approximate boundaries of the T3SS-1 islands based on gene annotation

A single gene cluster encoding a type III secretion system (T3SS) or “injectisome”, i.e. a needle-like apparatus involved in protein secretion across a host cell membrane, was identified in the *P. vermicola* DSM_17385 genome. The cluster comprising 22 kb and 23 ORFs was highly similar (app. 65% of pairwise identity) in gene orientation and putative gene function to a T3SS-1 island of *P. rettgeri* Dmel1 (Fig. 7); in particular, both clusters comprised a gene encoding an InvA-type ATPase and were located in a region of synteny that is inversed in *P. sneebia*. In contrast, no region of significant similarity to a second T3SS-2 island comprising a Ysc-type ATPase gene that is present in insect-associated *P. sneebia* and *P. burhodogranariea* bacteria, was identified in the genome sequence under study.

### Plasmids

No sequences corresponding to the 5.6 kb plasmid pPRET1 previously detected in *P. rettgeri* Dmel1 or to the plasmids known from other *D. melanogaster* associated *Providencia* species [45] nor to the multi-drug resistance plasmids of *P. rettgeri* and *P. stuartii* [50] were identified in the *P. vermicola* DSM_17385 genome. However, one small non-transmissible plasmid was identified. pPVER1 comprised in length 3682 bp and contained 10 ORFs (Fig. 1b). ORF2, ORF3 and ORF4 encoded hypothetical proteins comprising deduced sequences of, respectively, 66, 146, and 147 amino acids with > 90% similarity to gene products encoded by a family of *qnrD*-carrying plasmids that have been described previously for several strains of *P. rettgeri* (plasmids pDIJ09–518a, pGHS09–09a, pAB213, pYPR25–3), *P. stuartii* (pMF1A) and *P. alcalifaciens* (pBT169) and further *Morganellaceae* bacteria [51] as well as to (partially truncated) gene products encoded by plasmid p3–000369 of *P. huaxiensis* [4]. ORF1b of pPVER1 encoded a hypothetical protein of 88 amino acids with lower similarity to presumed orthologs in the genomes of *Klebsiella, Enterobacter* and *Citrobacter* bacteria (app. 60% similarity) and of sporadic presence in genomes of several *P. rettgeri* (51%), *P. alcalifaciens* (51%), *P. stuartii* (39%) and *P. heimbachae* (37%) strains, whereas the partially overlapping ORF1a encoded a hypothetical gene product of 170 amino acids with no significant similarities identified across the Genbank database. Moreover, five short (< 100 bp) ORFs named ORF5 through ORF9 with no significant similarity hits across Genbank were found located up- and downstream of ORF1a/b. Two 24 bp imperfect inverted repeats with eight mismatches delineated the region comprising colinear ORF1a, ORF1b and ORF2 and consistently defined a mobile insertion cassette *(mic*) of 2663 bp bracketed by both copies of a presumed duplicated CA insertion site.

### Antibiotic resistance genes

Antibiotic resistance factors encoded in the *P. vermicola* DSM_17385 genome operate by four resistance mechanisms with antibiotic efflux being the predominant one, followed by antibiotic target alteration, antibiotic inactivation and reduced antibiotic uptake (Table 3, Suppl. Table S2, Additional file 3).

**Table 3:** Putative antibiotic resistance-associated factors (ARF) encoded by the *P. vermicola* DSM_17385 genome

ARF Acronym	ARF Family^**a**^	Best hit^**b**^ CARD	Best hit^**b**^ Genbank	Antibiotic class
**Antibiotic uptake reduction**				
OmpK37	porin	56% (98%)	91% (100%)	cephamycin, penem, monobactam, penam, cephalosporin, carbapenem
**Antibiotic efflux**				
AbeS	SMR	48% (100%)	89% (100%)	macrolides, aminocoumarin
CRP	transcriptional regulator	98% (78%)	100% (100%)	penam, macrolide, fluoroquinolone
CsrA (RsmA)	RND	88% (100%)	100% (100%)	phenicol, diaminopyrimidine, fluoroquinolone
Bcr1 (Bcr1)	MFS	44% (100%)	93% (100%)	bicyclomycinsulfonamid
Bcr1 (Bcr2/3)	MFS	34% (98%)	98% (100%)	bicyclomycin
EmrE2	SMR	54% (100%)	90% (100%)	macrolides, small polyaromatic cations, potentially ethidium bromide, erythromycinE. coli, tetraphenylphosphonium, methyl viologen, gentamicin, kanamycin, neomycin
MdtG (YceE)	MFS	70% (100%)	99% (98%)	phosphomycin, deoxycholate
MdtH	MFS	75% (100%)	98% (100%)	fluoroquinolone
Tet(59)	MFS	81% (100%)	79% (97%)	tetracycline
TolC	outer membrane efflux protein	65% (97%)	95% (99%)	macrolide, fluoroquinolone, aminoglycosides, carbapenem, cephalosporin, glycylcycline, cephamycin, penam, tetracycline, peptide antibiotics, aminocoumarin, rifamycin, phenicol, triclosan, penem
AcrA	RND	67% (98%)	95% (95%)	fluoroquinolone, cephalosporin, glycylcycline, penam, tetracycline, rifamycin, phenicols, triclosan
AcrB	RND	75% (98%)	98% (100%)	fluoroquinolone, cephalosporin, glycylcycline, penam, tetracycline, rifamycin, phenicols, triclosan
AcrR	transcriptional regulator	52% (100%)	94% (99%)	fluoroquinolone, cephalosporin, glycylcycline, penam, tetracycline, rifamycin, phenicols, triclosan
EmrA	MFS	56% (100%)	94% (100%)	fluoroquinolone, nalidixic acid, thiolactomycin
EmrB	MFS	66% (99%)	99% (99%)	fluoroquinolone, nalidixic acid, thiolactomycin
MacA (PvdR)	ABC	37% (94%)	90% (97%)	macrolideserythromycin
MacB (PvdT)	ABC	51% (100%)	89% (100%)	macrolideserythromycin
MdtA (YegM)	RND	57% (100%)	91% (100%)	aminocoumarin, novobiocin
MdtB (YegN)	RND	77% (99%)	94% (100%)	aminocoumarin, novobiocin
MdtC (YegO)	RND	73% (100%)	95% (100%)	aminocoumarin, novobiocin
MexG	RND	63% (96%)	92% (100%)	fluoroquinolone, tetracycline, acridine, vanadium, norfloxacin, acriflavin
MexH	RND	58% (96%)	93% (100%)	fluoroquinolone, tetracycline, acridine, vanadium, norfloxacin, acriflavin
MexI	RND	78% (100%)	96% (100%)	fluoroquinolone, tetracycline, acridine, vanadium, norfloxacin, acriflavin
KpnE (MdtJ)	SMR	64% (100%)	95% (100%)	macrolides, aminoglycosides, cephalosporin, tetracycline, peptide antibiotics, rifamycin
KpnF (MdtI)	SMR	71% (100%)	99% (82%)	macrolides, aminoglycosides, cephalosporin, tetracycline, peptide antibiotics, rifamycin
KpnG (EmrA)	MFS	65% (100%)	92% (100%)	peptide antibiotics, carbapenem, macrolides, cephalosporin, aminoglycosides, penam, fluoroquinolones, penem, nalidixic acid, thiolactomycin
KpnH (EmrB)	MFS	78% (99%)	98% (99%)	peptide antibiotics, carbapenem, macrolides, cephalosporin, aminoglycosides, penam, fluoroquinolones, penem, nalidixic acid, thiolactomycin
RosA	MFS	75% (99%)	96% (100%)	peptide antibioticspolymyxin B, fosmidomycin
RosB	MFS	69% (100%)	99% (100%)	peptide antibioticspolymyxin B, fosmidomycin
**Antibiotic inactivation**				
AAC(2′)-Ia	aminoglycoside acetyltransferase	70% (87%)	72% (100%)	aminoglycosides
CatIII	chloramphenicol acetyltransferase	56% (100%)	71% (100%)	phenicols
FosA7.5	phosphomycin thiol transferase	62% (98%)	74% (100%)	phosphomycin
NmcR	transcriptional regulator	56% (99%)	89% (100%)	penam, cephalosporin, cephamycin, carbapenem
SRT-2	beta-lactamase	56% (100%)	86% (99%)	cephalosporins
**Antibiotic target alteration**				
EF-Tu^c^	translation elongation factor Tu	95% (74%)	99% (100%)	pulvomycin-class of elfamycins
ErmX, (Erm34)	RNA methyltransferase	29% (95%)	98% (100%)	aminoglycosides
GyrA^c^	DNA gyrase subunit alpha	51% (100%)	97% (100%)	fluoroquinolone
GyrB^c^	DNA gyrase subunit beta	55% (96%)	99% (100%)	fluoroquinolone, aminocoumarin
MurA^c^	UDP-N-acetylglucosamine enolpyruvyl transferase	50% (99%)	97% (100%)	phosphomycin
RpoB^c^	RNA polymerase subunit beta	55% (100%)	99% (100%)	rifamycinrifampicin
Ugd (PmrE)	UDP-glucose 6-dehydrogenase, phosphorethanolamine transferase	73% (96%)	98% (100%)	peptide antibiotics, polymyxin
VanW	glycopeptide resistance protein	29% (97%)	92% (99%)	glycopeptidesvancomycin

^a^ Abbreviations used for ARF family designation: ABC: ATP-binding cassette antibiotic efflux pump; MFS: major facilitator superfamily antibiotic efflux pump; RND: resistance-nodulation-cell division antibiotic efflux pump; SMR: small multidrug resistance antibiotic efflux pump

^b^ Best hits indicated as percent sequence similarity (percent sequence coverage)

^c^ Gene carrying resistance-conferring point-mutation(s)

Among the different types of efflux pumps identified, orthologs of the SMR-type efflux pump EmrE [52], the peptide-potassium antiporter RosAB [53], the MFS-type efflux pumps MdtG [54], KpnEF [55] and KpnGH-TolC [56, 57] as well as the ABC-type transporter MacAB-TolC [58, 59] are ubiquitously distributed across the genus *Providencia,* whereas the SMR-type efflux pump AbeS [60] potentially confers macrolide and aminocoumarin resistance to *Providencia* bacteria belonging to the species *P. stuartii* and *P. rettgeri* clade A and the MFS-type transporter Tet (59) [61] is mainly responsible for widespread tetracycline resistance observed in *P. rettgeri, P. alcalifaciens* and *P. heimbachae* bacteria [44]. Further identified efflux pump orthologs as the TolC-dependent transporters MdtABC [62], AcrAB [63] and EmrAB [64], the OpmD-dependent RND-type pump MexGHI [65], the MFS-type systems MdtH [66] and Bcr1/2 [67] appear sporadically across the genus *Providencia*. The identified ortholog of the transcriptional *acrAB* operon repressor AcrR [68] has been found to carry two known (Y114F, V165I) and one previously undescribed (M109L) mutations potentially conferring or increasing resistance to a spectrum of antibiotics including tetracycline, phenicols, penam, triclosan and fluoroquinolones [69]. Orthologs of further potentially resistance-relevant regulators as the carbon storage regulator protein CsrA [70, 71] and the cAMP-activated global transcriptional repressor CRP [72] have expectedly been identified, but will most likely not have an immediate role in antibiotic resistance regulation in *P. vermicola* as their known respective targets, i.e. the efflux pumps MexEF-OprN [73] and MdtEF [74, 75], respectively, are lacking. Moreover, an ortholog of the alternative porin OmpK37 [76] that reduces permeability of the cell envelope for a range of beta-lactams and is present in almost all sequenced *Providencia* genomes [44], has been identified in *P. vermicola*.

Among those antibiotic resistance conferring factors that act by antibiotic inactivation or molecular target alteration and are virtually ubiquitous across published *Providencia* genomes [44], the *P. vermicola* DSM_17385 genome encodes an ortholog of an SRT-2 type beta-lactamase [77] conferring resistance to cephalosporins and comprises two identical genes encoding orthologs of the lipid A modifying phosphoethanolamine transferase PmrE [78] that confers resistance to antimicrobial peptides and polymyxin. An ortholog of the NmcR regulator of the class A beta-lactamase NmcA [79] has been found encoded in the *P. vermicola* genome; however, as no gene encoding an NmrA ortholog has been identified, its relevance for beta-lactamase resistance is unclear. Moreover, *P. vermicola* comprises an ortholog of both chloramphenicol acetyltransferase CAT-III [80] and aminoglycoside acetyltransferase AAC (2′)-Ia [81]; both factors are widespread in mostly clinical strains of *P. rettgeri* and *P. stuartii* [44].

Among the resistance genes occurring sporadically across the genus, the *P. vermicola* DSM_17385 genome comprises orthologs of the phosphomycin thiol transferase FosA7 [82, 83], the ErmX-type rRNA methyltransferase RsmA [84, 85] and an ortholog of the vancomycin resistance protein VanW [86, 87]. Several proteins involved in basic cellular processes as DNA gyrase subunits A and B [88, 89], the RNA polymerase beta subunit [90], translation elongation factor Tu [91] and UDP-N-acetylglucosamine enolpyruvyl transferase MurA [92] carry one or several point mutations conferring resistance to antibiotics as phosphomycin, fluoroquinolones, elfamycin or rifamycin.

All antibiotic resistance factors identified in *P. vermicola* DSM_17385 appear to be chromosomally encoded; no antibiotic or multi drug resistance (MDR) plasmids as, e.g., those found in *P. rettgeri* or *P. stuartii* were identified. Moreover, operons comprising both widespread and sporadically occurring resistance genes are widely distributed over the *P. vermicola* genome. In particular, neither class 1 or 2 integrons nor SXT element that have been described previously to carry accumulated resistance genes in a *Providencia* isolate assigned to the species *P. vermicola* [35], appear to be present in the genome as IntI1, IntI2 or SXT integrases and *qacE*, *qacEdelta1* or *sul1* genes were not identified in the genome under study.

## Discussion

Prior to sequencing of the genome of the *P. vermicola* type strain DSM_17385, three genome sequences from *Providencia* strains assigned to the species *P. vermicola* had been published. The present comparative analysis using ribosomal typing, phylogenetic reconstruction and digital DNA-DNA hybridization has revealed that the presumed 4 *P. vermicola* strains are correctly assigned to the genus *Providencia*, but do not belong to the same taxonomic species.

Phylogenetic reconstruction from 16S rRNA encoding sequences has been found not sufficiently phylogeny informative to provide sound species delineation in the present context, a problem reported earlier with respect to the genus *Providencia* [19, 24]. In contrast, phylogenetic reconstruction from housekeeping gene (hMLST) and ribosomal protein encoding gene (rMLST) datasets comprising marker sequences from the nomenclatural type strains of all currently recognized *Providencia* species has clearly demonstrated that strain DSM_17385 is not closely related to any of the other specific type strains and therefore, expectedly, rightfully represents the independent species *P. vermicola*. In particular, results obtained from the ribosomal marker dataset corroborate the respective earlier conclusions from hMLST based studies [13, 19]. Both phylogenies (Figs. 3 and 4) indicate that *P. vermicola* shares a common ancestor with *P. rettgeri* clade A (comprising strain Dmel1), *P. rettgeri* clade B (proposed to be organized into an independent species named *P. entomophila*) and *P. huaxiensis* before it is phylogenetically related to *P. alcalifaciens* (comprising strain Dmel2) or the still more more distantly related *Drosophila melanogaster* derived *P. sneebia* and *P. burhodogranariea* type strains. These results have been fully corroborated by digital DNA-DNA hybridization analysis. Moreover, this systematic situation is mirrored in the apparently “inconclusive” outcome when strain DSM_17385 is typed across 53 ribosomal marker alleles: as the *P. vermicola* type is not closely related to any of the *Providencia* species represented in the PubMLST database, no existing rST has been assigned to the DSM_17385 genome.

Unexpectedly in view of their previous taxonomic assignment, none of the three presumed *P. vermicola* genome strains appeared closely related to the *P. vermicola* type strain. The outcomes of phylogenetic reconstruction from hMLST and rMLST data sets, digital DNA-DNA hybridization and ribosomal typing are in line with the assignment of *Providencia* strain G1 to the species *P. rettgeri* (clade B), whereas strains LLDRA6 and P8538 appear most closely related to the *P. stuartii / P. thailandensis* species complex. The perfect ribosomal typing match of strain P8358 to the species *P. vermicola* can be judged a bioinformatic artefact as the P8538 genome itself currently serves – both erroneously and misleadingly - as unique reference for this species in the PubMLST database. Taking these results together, *Providencia* strains G1, LLDRA6 and P8538 appear inconsistently assigned to the taxonomic species *P. vermicola* as represented at the genomic level by the genome sequence of the nomenclatural type strain DSM_17385.

Among the 195 *Providencia* genomes currently available from the Genbank database uniquely that of strain MR4, assigned to the species *P. rettgeri*, displayed a comparatively closer molecular taxonomic relationship to *P. vermicola* DSM_17385 (Suppl. Figure S2): *Providencia* strain MR4 might, therefore, be considered a possible candidate for a further *P. vermicola* genome strain. Interestingly, strain MR4 has been isolated from medicinal plant material, more exactly from stem tissue of the Indian mallow, *Abutilon indicum*, in India (Genbank BioSample SAMN03646990) and is, therefore, geographically related to strain DSM_17385. However, with only 2132 ORFs identified on as much as 697 contigs, assembly of the MR4 whole genome shotgun is currently rather incomplete, hampering systematic comparative genomics or OrthoANI analyses. In previous studies, comparisons of pair-wise sequence similarities from the hMLST marker set have been employed to critically evaluate the distinct species status of *P. vermicola*, *P. rettgeri* clades A and B and *P. huaxiensis* [13]. Extending this approach to the systematic relationship of strains DSM_17385 and MR4 has demonstrated that results from both the hMLST and rMLST datasets are consistent with *P. vermicola* DSM_17385 and the presumed *P. rettgeri* strain MR4 belonging to different rather than to the same taxonomic species.

Taking the above results together, the sequence reported here represents not only the nomenclatural type strain, but the to date unique well-supported *P. vermicola* genome. Comparative genomics at the infra-specific level as, e.g., the identification of *P. vermicola*-specific genes is not feasible with the currently available genome data set.

Within the limits set by the mode of genome assembly, orientation of the 18 scaffolds making up the *P. vermicola* genome sequence alongside the genome of *P. rettgeri* strain Dmel1 revealed a very high degree of genomic synteny. In particular, two important genomic rearrangements that have been described earlier for the *P. sneebia* genome in comparison to the genomes of further insect-derived *Providencia* strains from the species *P. rettgeri, P. alcalifaciens* and *P. burhodogranariea* [45] were absent from the genome of *P. vermicola* DSM_17385.

The genome of *P. rettgeri* Dmel1 was found to share 3127 orthologous genes (78%) with that of *P. vermicola* DSM_17385. This is a higher number and fraction of common orthologs than in comparison with insect-associated *P. alcalifaciens* (2672, 70%), *P. burhodogranariea* (2654, 70%) or *P. sneebia* (2211, 58%) strains [45], reflecting the closer phylogenetic relationship of *P. vermicola* and *P. rettgeri.* Given the lack of large block insertions, deletions or rearrangements when comparing both genomes (Fig. 6), genetic speciation understood as the generation of a set of unique genes appears to be the result of many small scale gene gains or losses rather than few large scale events.

No plasmid related to those of insect-associated *Providencia* bacteria has been identified when assembling the *P. vermicola* genome sequence data, a finding that is in line with the generally high variability in identity, conservation and putative functional designation documented for these genetic elements [45]. The plasmid identified, pPVER1, belongs to a class of small non-conjugative *qnrD*-plasmids found mainly in *Morganellaceae* bacteria, including strains from several *Providencia* species [51, 93, 94]. Expression of the *qnrD* gene produces a pentapeptide repeat protein that confers resistance to (fluoro-)quinolone antibiotics by protecting the cellular targets, namely bacterial DNA gyrase and topoisomerase IV, from quinolone binding [95]. The 2683 bp comprising *qnrD*-plasmids from *P. rettgeri, P. alcalifaciens* and *P. stuartii* in addition to the *qnrD* gene typically contain three colinear open reading frames termed ORF2 through ORF4 encoding hypothetical proteins of yet unknown function. The *qnrD* gene and ORF2 are located on a putative *mic* element delineated by 24 bp imperfect inverted repeats. *qnrD*-plasmids from *P. rettgeri, P.stuartii* and *P. alcalifaciens* are highly homologous with pair-wise nucleotide sequence similarities typically ranging between 99.7% and identity.

pPVER1 from the *P. vermicola* DSM_17385 genome shares the same basic structure found in these *qnrD*-type plasmids with the important difference that 747 bp from within the putative *mic* element comprising the *qnrD* gene itself have been replaced by 1887 bp of a sequence comprising the partially overlapping ORFs 1a and 1b that encode two hypothetical proteins of unknown function. Pairwise sequence similarities between the conserved 1795 bp long segment of pPVER1 carrying ORF2 through ORF4 and the homologous region of *Providencia qnrD*-type plasmids are high, ranging from 91.7 to 93.0%, whereas no significant similarity can be detected between the ORF1a/b and *qnrD* regions. Most probably, pPVER1 has already been described previously when strain DSM_17385 was used as reference strain in a study investigating *qnrD* genes of *Proteeae* bacteria [96], but no respective DNA sequence has been published from these studies.

A further *qnrD*-type plasmid, named p3–000369, has been identified in the nomenclatural type strain of the *Providencia* species *P. huaxiensis* [4]. Interestingly, the *qnrD* gene region in this plasmid is replaced by a 979 bp long DNA sequence carrying three colinear ORFs that encode two subunits of a predicted helix-turn-helix transcriptional regulator and a hypothetical protein of unknown function. This region displays no significant similarity to the *Providencia qnrD*-type plasmids or pPVER1, but an almost identical region comprising three orthologous ORFs is encoded by the large (> 200 kb) plasmid of *P. rettgeri* strain BML2526. Moreover, plasmid p3–000369 contains important deletions in both ORF3 and ORF4.

It appears, therefore, that within the genus *Providencia* the region carrying the quinolone resistance gene in small *qnrD*-type plasmids underwent rearrangements including recombination events with both the bacterial chromosome and other plasmids. The rearranged region is part of a putative *mic* element defined by inverted repeats IRR and IRL carrying, respectively, one and two SNPs when compared for *P. vermicola* pPVER1*, P. rettgeri* pDIJ09–518a, *P. stuartii* pMF1A, *P. alcalifaciens* pBT169, and *P. huaxiensis* p3–000369. However, as only part of the putative *mic* element is rearranged and as no known mobilization structures as, e.g., *mob* genes were identified on these plasmids, the mechanism leading to these rearrangements is currently unclear. It appears most parsimonious to suppose that rearrangements within *qnrD*-type plasmids of the genus *Providencia* have occurred subsequently to the appearance of a precursor plasmid carrying *qnrD* within a *mic* element.

With respect to the nature and organization of identified antibiotic resistance determinants, *P. vermicola* DSM_17385 appears generally similar to non-clinical strains from other *Providencia* species including *P. rettgeri* and *P. stuartii*. Striking features are both the complete absence of multi drug resistance plasmids and integrons and the related absence of the range of beta-lactamases found in clinical *P. rettgeri* and *P. stuartii* strains that are the main sources of the MDR phenotype occurring in *Providencia* [41]. These findings are fully in line with the invertebrate pathogen *P. vermicola* being in its natural environment efficiently excluded from the propagation routes of MDR carrying genetic elements operating between human pathogens. Susceptibility to MDR plasmid acquisition will likely become a major criterion in the evaluation of *P. vermicola* for potential applications in biological pest control.

Gram-negative bacterial pathogens use type III secretion systems (T3SS) or “injectisomes” in a wide variety of physiological contexts to translocate effector proteins simultaneously across their own cell envelope and a eukaryotic host cell or vacuolar membrane. There are different T3SS families named according to the type of ATPase being part of the injectisome. T3SS structural components, but not the effectors translocated by them are encoded by gene clusters that have been transferred between bacteria by horizontal gene transfer [97]. T3SS gene clusters are widespread in *Providencia* bacteria [44].

The *P. vermicola* DSM_17385 genome contains a single T3SS island encoding an injectisome of the Inv-Spa family (Fig. 7). This type of T3SS, termed “T3SS-1”, is generally associated with host cell invasion, i.e. paradigmatically bacterial uptake by nonphagocytic cells triggered by induced actin reorganization, or intracellular survival as, e.g., in the case of the tsetse fly endosymbiont *Sodalis glossinidius* [98, 99] or the primary endosymbiont (SZPE) of the Maize weevil, *Sitophilus zeamais* [100]. The T3SS-1 gene cluster is almost ubiquitously distributed across genomes of *Providencia* and related *Proteus* bacteria [44] and has likely been acquired prior to speciation. In particular, a similar T3SS-1 gene cluster has been identified in insect-derived Dmel strains of the species *P. rettgeri, P. alcalifaciens* and *P. sneebia*, but not in *P. burhodogranariea* [45]. Moreover, *P. sneebia* and *P. burhodogranariea* genomes carry a T3SS-2 injectisome of the Ysc family that is generally associated with the extracellular localization of pathogens and appears to be absent from other *Providencia* genomes including those of strains *P. rettgeri* Dmel1*, P. alcalifaciens* Dmel1 and the *P. vermicola* strain under study. As T3SS-1 should likely be non-functional in *P. sneebia* due to disruption of the Inv-type ATPase ORF by a premature stop codon [45], it appears that insect-associated *P. sneebia* and *P. burhodogranariea* operate a T3SS-2 for extracellular, insect-associated *P. vermicola, P. rettgeri* and *P. alcalifaciens* a T3SS-1 for intracellular localization. As Inv-Spa-type injectisomes are widespread across the genus *Providencia*, but Ysc-type injectisomes appear to date limited to two closely related *Providencia* species, it is most parsimonious to assume that a common ancestor of *P. sneebia* and *P. burhodogranariea* has acquired a T3SS-2 island by, for instance, horizontal gene transfer, with subsequent mutational inactivation and – in *P. burhodogranariea* – loss of the original T3SS-1 gene cluster. Interestingly, a Ysc-type injectisome (T3SS-2) is present in the nematode-associated entomopathogenic bacterium *Photorhabdus luminescens* where it is involved in bacterial survival in the insect hemocoel and resistance to phagocytosis by macrophages [101].

## Conclusions

The genome of the nomenclatural type strain DSM_17385 of the nematode-associated insect-pathogenic enterobacterial species *P. vermicola* has been sequenced and analyzed. The sequence reported represents the first well-supported published genome for the taxonomic species *P. vermicola* to be used as reference in further comparative genomics studies on *Providencia* bacteria. Genomic analysis has confirmed a closer phylogenetic relationship of *P. vermicola* to the *P. rettgeri* species complex including the recently proposed species *P. huaxiensis* and *P. entomophila* than to further *Providencia* species. The genome shows a high degree of synteny when compared to *P. rettgeri* strain Dmel1 isolated from *D. melanogaster* with 78% of the identified genes being present in both genomes. As most *Providencia* strains sequenced to date, *P. vermicola* DSM_17385 carries a type III secretion system (T3SS-1) with probable function in host cell invasion or intracellular survival and might therefore differ fundamentally in its mechanism of pathogenesis from insect-pathogenic *P. sneebia* or *P. burhodogranariea* bacteria carrying a different type of injectísome. Potentially antibiotic resistance-associated genes comprising numerous efflux pumps and point-mutated house-keeping genes, have been identified across the *P. vermicola* DSM_17385 genome. However, no antibiotic resistance gene carrying plasmids or mobile genetic elements as those causing MDR phenomena in clinical *Providencia* strains have been found. The only identified plasmid, pPVER1, is derived from a fluoroquinolone resistance plasmid family, but has lost the *qnrD* resistance gene by recombination from within the plasmid-encoded *mic* element. We conclude that the invertebrate pathogen *P. vermicola* is in its natural environment efficiently excluded from the propagation routes of MDR carrying genetic elements operating between human pathogens.

## Methods

### Bacterial cultivation and DNA extraction

The nomenclatural type strain *P. vermicola* DSM_17385 (= CIP_108829 = OP1) has been received from the German Collection of Microorganisms and Cell Cultures (DSMZ; https://www.dsmz.de). The strain had originally been isolated from surface-sterilized infective juveniles of entomoparasitic *Steinernema thermophilum* nematodes extracted from larvae of the greater wax moth (*Galleria mellonella*) [24]. For DNA extraction, bacteria were grown to late log phase in LB medium (10 g/l Tryptone, 5 g/l Yeast Extract, 5 g/l sodium chloride, pH = 7.0) containing 50 μg/ml tetracycline. DNA was extracted using the DNeasy Blood & Tissue kit protocol for Gram negative bacteria as provided by the manufacturer (Qiagen). Genomic DNA was eluted in 10 mM TrisCl (pH 8.5). DNA quality and quantity were controlled electrophoretically and using a NanoDrop NT-1000 UV spectrophotometer.

### Genomic sequencing, assembly and gene annotation

The whole genome sequencing was performed (SEQ-IT, Kaiserslautern, Germany) on the Illumina MiSeq platform (Illumina, Inc., San Diego, CA), producing 2 × 250-bp end-paired reads, generating a total of 1,530,356 reads with ~ 90× coverage. The Trimmomatic algorithm (version 0.36) [102] was used to trim all the generated reads and their quality evaluated with in-house scripts using FastQC (version 0.11.9) [103], BedTools (version 2.25.0) [104], and SAMtools (version 1.3.1) [105] algorithms. High-quality filtered reads were subsequently de novo assembled using the SPADES assembler (version 3.7.1) [106].

GLIMMER (version 3) prokaryotic genome automatic annotation software was used to annotate this genome [107]. The size, GC content, number of contigs, N50, L50, average coverage, as well as the number of RNAs, tRNAs, and protein-coding sequences obtained for our isolate, can be found in Table 1. The structural RNA encoding genes were identified using tRNAscan-SE version 2.0.7 [108] and RNAmmer version 1.2 [109]. Finally, the circular multi-track plot was carried out using the ‘RCircos’ R software package [110].

An in-house pipeline was developed to annotate *P. vermicola* antibiotic resistance genes using the BLASTp algorithm (E-value < 1e-10 and %identity ≥50%). The queried DSM_17385 genes were identified by mapping protein sequences to the CARD database [111] and tBlastN similarity searches across annotated *Providencia* genomes. Blast2GO was used to provide automatic high-throughput annotation, gene ontology mapping and functional categorization of *P. vermicola* ORFs identified by GLIMMER. Finally, orthologous genes were evaluated using clusters of orthologous genes (COGs) and eggNOG [112, 113].

The complete plasmid assembly of *pPVER1* was performed utilizing plasmiSPAdes (SPAdes v3.7.1) software with minor manual curation [109].

### Prediction of *Providencia* orthologs and digital DNA-DNA hybridization analysis

Orthology analysis was conducted on proteomes of *P. vermicola* DSM_17385 and *P. rettgeri* Dmel1 using Inparanoid software with default parameters [114]. We used a confidence score threshold = 1 to directly estimate orthology relationships between the identified protein-encoding genes.

Orthologous Average Nucleotide Identity Tool (OAT) software v0.9.31 (https://www.ezbiocloud.net/tools/orthoani) [49] was used for calculation of pair-wise OrthoANI values for a set of published genomes comprising the four *Providencia* strains previously assigned to the species *P. vermicola,* the nomenclatural type strains representing all currently recognized taxonomic *Providencia* species (with the notable exception of the still insufficiently assembled genome of the *P. stuartii* type strain NCTC_11800), and four strains selected on the basis of the hMLST based phylogenetic analysis shown in Suppl. Figure 2 as the most closely related well annotated genomes with respect to the *P. stuartii* type strain (i.e. surrogate type genome of *P. stuartii* strain FDA-ARGOS 645) and to the three presumed *P. vermicola* strains G1, P8358 and LLDRA6.

### Whole-genome alignment

The genomic DNA sequences of *P. vermicola* DSM_17385 and *P. rettgeri* Dmel1 (NZ_CM001774) were aligned using LASTZ (Large-Scale Genome Alignment Tool) (version 1.02.00) with default parameters [115]. Syntenic chromosomal regions were identified using the MAUVE (Multiple Alignment of Conserved Genomic Sequence with Rearrangements) software package. To determine a reasonable value for the Min Locally Collinear Blocks (LCBs), we performed an initial alignment at the default value and then used the LCB weight slider in the MAUVE graphical user interface (GUI) to fix parameters empirically eliminating all spurious rearrangements. Sequences were then realigned using the manually determined weight value. The T3SS-1 sequences of *P. vermicola* DSM_17385 and *P. rettgeri* Dmel1 (NZ_CM001774) were aligned using ClustalW (version 2.1) with default settings [116].

### Multilocus sequence typing and phylogenetic reconstruction

The 16S rRNA, hMLST and rMLST marker genes identified in the annotated *P. vermicola* DSM_17385 genome (Additional files 1 and 2) were used as query in separate BlastN searches [117] across completed genome and whole genome shotgun entries of the Genbank database assigned to the genus *Providencia* (taxid 586). Orthologous genes from the type strain DSM_4479 (= ATCC_29906) of the related enterobacterium *Proteus mirabilis* were concomitantly identified to serve as outgroup for phylogenetic reconstruction. A whole genome shotgun sequence assigned to `*Candidatus* Providencia siddallii´ was not considered for sequence typing as preliminary analysis revealed that sequences were too highly divergent to be relevant for the problem under study. For each hMLST and rMLST marker, a set of orthologs from reference genomes representing the 10 recognized *Providencia species* was generated, each species being represented by its nomenclatural type strain and – if available - further strains spanning the known range of diversity included under this taxon.

Marker alignment and phylogenetic reconstruction were performed using the MEGA software tool [118] at the level of hMLST and rMLST meta-genes comprising concatenations of all respective single marker sequences. Phylogenies were reconstructed using a p-distance matrix-based Neighbor Joining (NJ) method as implemented in MEGA. Tree topology confidence limits were explored in non-parametric bootstrap analyses over 1,000 pseudo-replicates.

For rMLST, *P. vermicola* DSM_17385 genome data and the three published genomes assigned to this species were compared to the PubMLST database [47]. The rMLST typing tool compares each *rps, rpl* and *rpm* gene sequence of the query genome to an allele-specific reference database, identifies the closest ribosomal sequence type or types (rST) in the database, and translates this rST similarity into a taxonomic assignment.

## Supplementary Information

**Additional file 1. **Sequences of *Providencia vermicola* DSM_17385 16S rRNA and hMLST marker genes.

**Additional file 2. **Sequences of *Providencia vermicola* DSM_17385 rMLST marker genes.

**Additional file 3. **Sequences of *Providencia vermicola* DSM_17385 putative antibiotic.resistance-associated proteins

**Additional file 4: Suppl. Figure S1.** Neighbor Joining (NJ) phylogeny of *Providencia* bacteria as reconstructed from complete 16S ribosomal RNA gene sequences. Terminal branches are labelled by genus, species and strain designations as well as GenBank accession numbers; “TYPE” indicates nomenclatural type strains of the respective taxonomic species. Bacterial strains that have been assigned to the species *P. vermicola* are in bold type. The phylogram representation of the tree has been expanded into a cladogram for better resolution and easier bootstrap support indication. Numbers on branches of the cladogram indicate bootstrap support percentages. The size bar corresponds to 0.5% sequence divergence along phylogram branches. The 16S rRNA encoding sequence from the closely related bacterium *Proteus mirabilis* has been used as outgroup.

**Additional file 5: Suppl. Figure S2.** Neighbor Joining (NJ) phylogeny of *Providencia* bacteria as reconstructed from concatenated complete *fusA, gyrB, ileS, lepA* and *leuS* gene sequences. Terminal branches are labelled by GenBank accession numbers followed by genus, species and strain designations. *Providencia* isolates provisionally assigned to the species *P. vermicola* and nomenclatural type strains representing the currently recognized *Providencia* species are presented in bold type; type strains are indicated by the word “TYPE” following the species designation. Numbers on branches indicate bootstrap support percentages superior to 50%. The size bar corresponds to 2% sequence divergence. The concatenation of orthologous sequences from the closely related bacterium *Proteus mirabilis* has been used as outgroup.

**Additional file 6: Suppl. Table S1.** Pair-wise sequence similarity (in %) matrices for hMLST (upper right) and rMLST (lower left) data sets across the genus *Providencia*.

**Additional file 7: Suppl. Table S2.** Putative antibiotic resistance-associated factors of *P. vermicola* DSM_17385 as identified in the CARD database.

## Data Availability

The *P. vermicola* genome sequence has been deposited in the GenBank database under the accession number JAGSPI 000000000. The version described in this paper is version JAGSPI 010000000. The corresponding BioProject accession number is PRJNA723267 (https://www.ncbi.nlm.nih.gov/bioproject/PRJNA723267). The *P. vermicola* type strain is available from both the German Collection of Microorganisms and Cell Cultures (DSMZ; https://www.dsmz.de) under the accession number DSM_17385 and the Biological Resources Centre of Institut Pasteur (CRBIP; https://catalogue-crbip.pasteur.fr) under the accession number CIP_108829T.

## Abbreviations

ABC: ATP-Binding Cassette antibiotic efflux pump
cAMP: cyclic adenosine monophophate
CARD: Comprehensive Antibiotic Resistance Database
COG: Cluster of Orthologous Genes
GLIMMER: Gene Locator and Interpolated Markov ModelER
GO: Gene Ontology
GUI: Graphical User Interface
hMLST: Multilocus Sequence Typing using housekeeping genes
InvA: Largest inner membrane export protein in T3SS of *Salmonella* spp
Inv-Spa: Highly conserved inner membrane proteins in T3SS of *Salmonella* spp
IRL/IRR: Left−/Right-hand Inverted Repeat
LASTZ: Large-Scale Genome Alignment Tool
LCBs: Locally Collinear Blocks
MAUVE: Multiple Alignment of Conserved Genomic Sequence with Rearrangements
MDR: Multi Drug Resistance
MEGA: Molecular Evolutionary Genetics Analysis software
MFS: Major Facilitator Superfamily antibiotic efflux pump
*mic*: mobile insertion cassette
NJ: Neighbor Joining algorithm for phylogenetic reconstruction
ORF: Open Reading Frame
PubMLST: Public databases for molecular typing and microbial genome diversity
*qnrD*: quinolone resistance gene D
rMLST: Multilocus Sequence Typing using ribosomal protein encoding genes
RND: Resistance-Nodulation-cell Division antibiotic efflux pump
rST: ribosomal Sequence Type
SMR: Small Multidrug Resistance antibiotic efflux pump
SNP: Single Nucleotide Polymorphism
SPAdes: St. Petersburg genome assembler
SRT-2: Beta-lactamase conferring resistance to cefotaxime in *Serratia marcescens*
SXT element: Integrating Conjugative Element, termed for sulfamethoxazole-trimethoprim resistance
T3SS: Type III Secretion System
Ysc: Highly conserved inner membrane proteins in T3SS of *Yersinia* spp

## References

[1] Parte AC, Sardà Carbasse J, Meier-Kolthoff JP, Reimer LC, Göker M (2020). List of prokaryotic names with standing in nomenclature (LPSN) moves to the DSMZ. Int J Syst Evol Microbiol.

[2] Mohr O'Hara C, Steigerwalt AG, Green D, McDowell M, Hill BC, Brenner DJ, Miller JM (1999). Isolation of Providencia heimbachae from human feces. J Clin Microbiol.

[3] O'Hara CM, Brenner FW, Miller JM (2000). Classification, identification, and clinical significance of Proteus, Providencia, and Morganella. Clin Microbiol Rev.

[4] Hu Y, Feng Y, Zhang X, Zong Z (2019). Providencia huaxiensis sp. nov., recovered from a human rectal swab. Int J Syst Evol Microbiol.

[5] Yoh M, Matsuyama J, Ohnishi M, Takagi K, Miyagi H, Mori K, Park KS, Ono T, Honda T (2005). Importance of Providencia species as a major cause of travellers' diarrhoea. J Med Microbiol.

[6] Shima A, Hinenoya A, Asakura M, Nagita A, Yamasaki S (2012). Prevalence of Providencia strains among children with diarrhea in Japan. Jpn J Infect Dis.

[7] Shah MM, Odoyo E, Ichinose Y (2019). Epidemiology and pathogenesis of Providencia alcalifaciens infections. Am J Trop Med Hyg.

[8] Linhares I, Raposo T, Rodrigues A, Almeida A (2013). Frequency and antimicrobial resistance patterns of bacteria implicated in community urinary tract infections: a ten-year surveillance study (2000-2009). BMC Infect Dis.

[9] Stock I, Wiedemann B (1998). Natural antibiotic susceptibility of Providencia stuartii, P. rettgeri, P. alcalifaciens and P. rustigianii strains. J Med Microbiol.

[10] Lee HW, Kang HY, Shin KS, Kim J (2007). Multidrug-resistant Providencia isolates carrying blaPER-1, blaVIM-2, and armA. J Microbiol.

[11] Shin S, Jeong SH, Lee H, Hong JS, Park MJ, Song W (2018). Emergence of multidrug-resistant Providencia rettgeri isolates co-producing NDM-1 carbapenemase and PER-1 extended-spectrum β-lactamase causing a first outbreak in Korea. Ann Clin Microbiol Antimicrob.

[12] Liu J, Wang R, Fang M (2020). Clinical and drug resistance characteristics of Providencia stuartii infections in 76 patients. J Int Med Res.

[13] Ksentini I, Gharsallah H, Sahnoun M, Schuster C, Hamli Amri S, Gargouri R, Triki MA, Ksantini M, Leclerque A (2019). *Providencia entomophila sp. nov.,* a new bacterial species associated with major olive pests in Tunisia. PLoS One.

[14] Khan KA, Ansari MJ, Al-Ghamdi A, Nuru A, Harakeh S, Iqbal J (2017). Investigation of gut microbial communities associated with indigenous honey bee (Apis mellifera jemenitica) from two different eco-regions of Saudi Arabia. Saudi J Biol Sci.

[15] Gupta AK, Nayduch D, Verma P, Shah B, Ghate HV, Patole MS, Shouche YS (2012). Phylogenetic characterization of bacteria in the gut of house flies (Musca domestica L.). FEMS Microbiol Ecol.

[16] Maleki-Ravasan N, Ahmadi N, Soroushzadeh Z, Raz AA, Zakeri S, Dinparast DN (2020). New insights into Culturable and Unculturable Bacteria across the life history of medicinal maggots Lucilia sericata (Meigen) (Diptera: Calliphoridae). Front Microbiol.

[17] Duan R, Xu H, Gao S, Gao Z, Wang N (2020). Effects of different hosts on bacterial communities of parasitic wasp Nasonia vitripennis. Front Microbiol.

[18] Kuzina LV, Peloquin JJ, Vacek DC, Miller TA (2001). Isolation and identification of bacteria associated with adult laboratory Mexican fruit flies, Anastrepha ludens (Diptera: Tephritidae). Curr Microbiol.

[19] Juneja P, Lazzaro BP (2009). Providencia sneebia sp. nov. and Providencia burhodogranariea sp. nov., isolated from wild Drosophila melanogaster. Int J Syst Evol Microbiol.

[20] Galac MR, Lazzaro BP (2011). Comparative pathology of bacteria in the genus Providencia to a natural host, *Drosophila melanogaster*. Microbes Infect.

[21] Msaad Guerfali M, Djobbi W, Charaabi K, Hamden H, Fadhl S, Marzouki W, Dhaouedi F, Chevrier C (2018). Evaluation of Providencia rettgeri pathogenicity against laboratory Mediterranean fruit fly strain (Ceratitis capitata). PLoS One.

[22] Hadapad AB, Shettigar SKG, Hire RS (2019). Bacterial communities in the gut of wild and mass-reared Zeugodacus cucurbitae and Bactrocera dorsalis revealed by metagenomic sequencing. BMC Microbiol.

[23] De Cock M, Virgilio M, Vandamme P, Bourtzis K, De Meyer M, Willems A (2020). Comparative Microbiomics of Tephritid frugivorous pests (Diptera: Tephritidae) from the field: a tale of high variability across and within species. Front Microbiol.

[24] Somvanshi VS, Lang E, Sträubler B, Spröer C, Schumann P, Ganguly S, Saxena AK, Stackebrandt E (2006). Providencia vermicola sp. nov., isolated from infective juveniles of the entomopathogenic nematode Steinernema thermophilum. Int J Syst Evol Microbiol.

[25] Yi YK, Park HW, Shrestha S, Seo J, Kim YO, Shin CS, Kim Y (2007). Identification of two entomopathogenic bacteria from a nematode pathogenic to the oriental beetle, Blitopertha orientalis. J Microbiol Biotechnol.

[26] Park HW, Kim YO, Ha JS, Youn SH, Kim HH, Bilgrami AL, Shin CS (2011). Effects of associated bacteria on the pathogenicity and reproduction of the insect-parasitic nematode Rhabditis blumi (Nematoda: Rhabditida). Can J Microbiol.

[27] Sangeetha BG, Jayaprakas CA, Siji JV, Rajitha M, Shyni B, Mohandas C (2016). Molecular characterization and amplified ribosomal DNA restriction analysis of entomopathogenic bacteria associated with Rhabditis (Oscheius) spp. 3 Biotech.

[28] Jackson TJ, Wang H, Nugent MJ, Griffin CT, Burnell AM, Dowds BCA (1995). Isolation of insect pathogenic bacteria, *Providencia rettgeri*, from Heterorhabditis spp. J Appl Bacteriol.

[29] Gegner T, Carrau T, Vilcinskas A, Lee KZ (2018). The infection of Harmonia axyridis by a parasitic nematode is mediated by entomopathogenic bacteria and triggers sex-specific host immune responses. Sci Rep.

[30] Goodrich-Blair H, Clarke DJ (2007). Mutualism and pathogenesis in Xenorhabdus and Photorhabdus: two roads to the same destination. Mol Microbiol.

[31] Chaston JM, Suen G, Tucker SL, Andersen AW, Bhasin A, Bode E, Bode HB, Brachmann AO, Cowles CE, Cowles KN, Darby C, de Léon L, Drace K, du Z, Givaudan A, Herbert Tran EE, Jewell KA, Knack JJ, Krasomil-Osterfeld KC, Kukor R, Lanois A, Latreille P, Leimgruber NK, Lipke CM, Liu R, Lu X, Martens EC, Marri PR, Médigue C, Menard ML, Miller NM, Morales-Soto N, Norton S, Ogier JC, Orchard SS, Park D, Park Y, Qurollo BA, Sugar DR, Richards GR, Rouy Z, Slominski B, Slominski K, Snyder H, Tjaden BC, van der Hoeven R, Welch RD, Wheeler C, Xiang B, Barbazuk B, Gaudriault S, Goodner B, Slater SC, Forst S, Goldman BS, Goodrich-Blair H (2011). The entomopathogenic bacterial endosymbionts Xenorhabdus and Photorhabdus: convergent lifestyles from divergent genomes. PLoS One.

[32] Balasubramani G, Deepak P, Sowmiya R, Ramkumar R, Perumal P (2015). Antigonon leptopus: a potent biological source for extermination of fish bacterial pathogens Providencia and Aeromonas. Nat Prod Res.

[33] Boumerdassi H, Djouadi LN, Ouar-Korichi M, Ouzari H-I, Nateche F. Isolation, Characterization of ichtyopathogenic bacterial strain in a lake ecosystem in Algeria. In: Proceedings of the 17th International Days of Biotechnology, 20.-23.12.2018. Sousse: ATBT Press; 2018:72.

[34] Preena PG, Dharmaratnam A, Raj NS, Raja SA, Nair RR, Swaminathan TR (2020). Antibiotic-resistant Enterobacteriaceae from diseased freshwater goldfish. Arch Microbiol.

[35] Rajpara N, Kutar BM, Sinha R, Nag D, Koley H, Ramamurthy T, Bhardwaj AK (2015). Role of integrons, plasmids and SXT elements in multidrug resistance of vibrio cholerae and Providencia vermicola obtained from a clinical isolate of diarrhea. Front Microbiol.

[36] Liu Y, Chang H, Li Z, Feng Y, Cheng D, Xue J (2017). Biodegradation of gentamicin by bacterial consortia AMQD4 in synthetic medium and raw gentamicin sewage. Sci Rep.

[37] Islam F, Yasmeen T, Ali Q, Mubin M, Ali S, Arif MS, Hussain S, Riaz M, Abbas F (2016). Copper-resistant bacteria reduces oxidative stress and uptake of copper in lentil plants: potential for bacterial bioremediation. Environ Sci Pollut Res Int.

[38] Sharma J, Shamim K, Dubey SK, Meena RM (2017). Metallothionein assisted periplasmic lead sequestration as lead sulfite by Providencia vermicola strain SJ2A. Sci Total Environ.

[39] Tan L, Wu H, Cui H, Xu H, Xu M, Xiao Y, Qiu G, Liu X, Dong H, Xie J (2020). Selective adsorption of palladium and platinum from secondary wastewater using Escherichia coli BL21 and Providencia vermicola. Bioprocess Biosyst Eng.

[40] Shukla A, Parmar P, Goswami D, Patel B, Saraf M (2021). Exemplifying an archetypal thorium-EPS complexation by novel thoriotolerant Providencia thoriotolerans AM3. Sci Rep.

[41] Piza-Buitrago A, Rincón V, Donato J, Saavedra SY, Duarte C, Morero J, Falquet L, Reguero MT, Barreto-Hernández E (2020). Genome-based characterization of two Colombian clinical Providencia rettgeri isolates co-harboring NDM-1, VIM-2, and other β-lactamases. BMC Microbiol.

[42] Abdallah M, Balshi A (2018). First literature review of carbapenem-resistant Providencia. New Microbes New Infect.

[43] Li D, Li R, Ding Z, Ruan X, Luo J, Chen J, Zheng J, Tang J (2020). Discovery of a novel native bacterium of Providencia sp with high biosorption and oxidation ability of manganese for bioleaching of heavy metal contaminated soils. Chemosphere.

[44] Yuan C, Wei Y, Zhang S, Cheng J, Cheng X, Qian C, Wang Y, Zhang Y, Yin Z, Chen H (2020). Comparative genomic analysis reveals genetic mechanisms of the variety of pathogenicity, antibiotic resistance, and environmental adaptation of Providencia genus. Front Microbiol.

[45] Galac MR, Lazzaro BP (2012). Comparative genomics of bacteria in the genus Providencia isolated from wild Drosophila melanogaster. BMC Genomics.

[46] Tatusov RL, Natale DA, Garkavtsev IV, Tatusova TA, Shankavaram UT, Rao BS, Kiryutin B, Galperin MY, Fedorova ND, Koonin EV (2001). The COG database: new developments in phylogenetic classification of proteins from complete genomes. Nucleic Acids Res.

[47] Jolley KA, Bliss CM, Bennett JS, Bratcher HB, Brehony C, Colles FM, Wimalarathna H, Harrison OB, Sheppard SK, Cody AJ, Maiden MCJ (2012). Ribosomal multilocus sequence typing: universal characterization of bacteria from domain to strain. Microbiology.

[48] Lalucat J, Mulet M, Gomila M, García-Valdés E (2020). Genomics in bacterial taxonomy: impact on the genus *Pseudomonas*. Genes (Basel).

[49] Lee I, Ouk Kim Y, Park SC, Chun J (2016). OrthoANI: an improved algorithm and software for calculating average nucleotide identity. Int J Syst Evol Microbiol.

[50] Miró E, Grünbaum F, Gómez L, Rivera A, Mirelis B, Coll P, Navarro F (2013). Characterization of aminoglycoside-modifying enzymes in enterobacteriaceae clinical strains and characterization of the plasmids implicated in their diffusion. Microb Drug Resist.

[51] Guillard T, Cambau E, Neuwirth C, Nenninger T, Mbadi A, Brasme L, Vernet-Garnier V, Bajolet O, de Champs C (2012). Description of a 2,683-base-pair plasmid containing qnrD in two Providencia rettgeri isolates. Antimicrob Agents Chemother.

[52] Schuldiner S (2009). EmrE, a model for studying evolution and mechanism of ion-coupled transporters. Biochim Biophys Acta.

[53] Bengoechea JA, Skurnik M (2000). Temperature-regulated efflux pump/potassium antiporter system mediates resistance to cationic antimicrobial peptides in Yersinia. Mol Microbiol.

[54] Fàbrega A, Martin RG, Rosner JL, Tavio MM, Vila J (2010). Constitutive SoxS expression in a fluoroquinolone-resistant strain with a truncated SoxR protein and identification of a new member of the marA-soxS-rob regulon, mdtG. Antimicrob Agents Chemother.

[55] Srinivasan VB, Rajamohan G (2013). KpnEF, a new member of the Klebsiella pneumoniae cell envelope stress response regulon, is an SMR-type efflux pump involved in broad-spectrum antimicrobial resistance. Antimicrob Agents Chemother.

[56] Lomovskaya O, Lewis K (1992). Emr, an Escherichia coli locus for multidrug resistance. Proc Natl Acad Sci U S A.

[57] Srinivasan VB, Singh BB, Priyadarshi N, Chauhan NK, Rajamohan G (2014). Role of novel multidrug efflux pump involved in drug resistance in Klebsiella pneumoniae. PLoS One.

[58] Kobayashi N, Nishino K, Hirata T, Yamaguchi A (2003). Membrane topology of ABC-type macrolide antibiotic exporter MacB in Escherichia coli. FEBS Lett.

[59] Xu Y, Sim SH, Song S, Piao S, Kim HM, Jin XL, Lee K, Ha NC (2010). The tip region of the MacA alpha-hairpin is important for the binding to TolC to the Escherichia coli MacAB-TolC pump. Biochem Biophys Res Commun.

[60] Srinivasan VB, Rajamohan G, Gebreyes WA (2009). Role of AbeS, a novel efflux pump of the SMR family of transporters, in resistance to antimicrobial agents in Acinetobacter baumannii. Antimicrob Agents Chemother.

[61] Leclercq SO, Wang C, Zhu Y, Wu H, Du X, Liu Z, Feng J (2016). Diversity of the tetracycline Mobilome within a Chinese pig manure sample. Appl Environ Microbiol.

[62] Nagakubo S, Nishino K, Hirata T, Yamaguchi A (2002). The putative response regulator BaeR stimulates multidrug resistance of Escherichia coli via a novel multidrug exporter system, MdtABC. J Bacteriol.

[63] Du D, Wang Z, James NR, Voss JE, Klimont E, Ohene-Agyei T (2014). Structure of the AcrAB-TolC multidrug efflux pump. Nature.

[64] Lomovskaya O, Lewis K, Matin A (1995). EmrR is a negative regulator of the Escherichia coli multidrug resistance pump EmrAB. J Bacteriol.

[65] Aendekerk S, Diggle SP, Song Z, Høiby N, Cornelis P, Williams P, Cámara M (2005). The MexGHI-OpmD multidrug efflux pump controls growth, antibiotic susceptibility and virulence in Pseudomonas aeruginosa via 4-quinolone-dependent cell-to-cell communication. Microbiology.

[66] Hirakawa H, Inazumi Y, Masaki T, Hirata T, Yamaguchi A (2005). Indole induces the expression of multidrug exporter genes in Escherichia coli. Mol Microbiol.

[67] Fonseca EL, Marin MA, Encinas F, Vicente AC (2015). Full characterization of the integrative and conjugative element carrying the metallo-β-lactamase Bla SPM-1 and bicyclomycin bcr1 resistance genes found in the pandemic Pseudomonas aeruginosa clone SP/ST277. J Antimicrob Chemother.

[68] Schneiders T, Amyes SG, Levy SB (2003). Role of AcrR and ramA in fluoroquinolone resistance in clinical Klebsiella pneumoniae isolates from Singapore. Antimicrob Agents Chemother.

[69] Webber MA, Talukder A, Piddock LJ (2005). Contribution of mutation at amino acid 45 of AcrR to acrB expression and ciprofloxacin resistance in clinical and veterinary Escherichia coli isolates. Antimicrob Agents Chemother.

[70] Pessi G, Williams F, Hindle Z, Heurlier K, Holden MT, Cámara M (2001). The global posttranscriptional regulator RsmA modulates production of virulence determinants and N-acylhomoserine lactones in *Pseudomonas aeruginosa*. J Bacteriol.

[71] Mulcahy H, O'Callaghan J, O'Grady EP, Adams C, O'Gara F (2006). The posttranscriptional regulator RsmA plays a role in the interaction between Pseudomonas aeruginosa and human airway epithelial cells by positively regulating the type III secretion system. Infect Immun.

[72] Nishino K, Senda Y, Yamaguchi A (2008). CRP regulator modulates multidrug resistance of Escherichia coli by repressing the mdtEF multidrug efflux genes. J Antibiot.

[73] Köhler T, Epp SF, Curty LK, Pechère JC (1999). Characterization of MexT, the regulator of the MexE-MexF-OprN multidrug efflux system of Pseudomonas aeruginosa. J Bacteriol.

[74] Nishino K, Yamaguchi A (2002). EvgA of the two-component signal transduction system modulates production of the yhiUV multidrug transporter in Escherichia coli. J Bacteriol.

[75] Nishino K, Yamaguchi A (2004). Role of histone-like protein H-NS in multidrug resistance of Escherichia coli. J Bacteriol.

[76] Doménech-Sánchez A, Hernández-Allés S, Martínez-Martínez L, Benedí VJ, Albertí S (1999). Identification and characterization of a new porin gene of Klebsiella pneumoniae: its role in beta-lactam antibiotic resistance. J Bacteriol.

[77] Wu LT, Tsou MF, Wu HJ, Chen HE, Chuang YC, Yu WL (2004). Survey of CTX-M-3 extended-spectrum beta-lactamase (ESBL) among cefotaxime-resistant Serratia marcescens at a medical center in middle Taiwan. Diagn Microbiol Infect Dis.

[78] Gunn JS, Lim KB, Krueger J, Kim K, Guo L, Hackett M, Miller SI (1998). PmrA-PmrB-regulated genes necessary for 4-aminoarabinose lipid a modification and polymyxin resistance. Mol Microbiol.

[79] Naas T, Nordmann P (1994). Analysis of a carbapenem-hydrolyzing class a beta-lactamase from Enterobacter cloacae and of its LysR-type regulatory protein. Proc Natl Acad Sci U S A.

[80] Murray IA, Hawkins AR, Keyte JW, Shaw WV (1988). Nucleotide sequence analysis and overexpression of the gene encoding a type III chloramphenicol acetyltransferase. Biochem J.

[81] Rather PN, Orosz E, Shaw KJ, Hare R, Miller G (1993). Characterization and transcriptional regulation of the 2′-N-acetyltransferase gene from Providencia stuartii. J Bacteriol.

[82] Rehman MA, Yin X, Persaud-Lachhman MG, Diarra MS (2017). First detection of a Fosfomycin resistance gene, fosA7, in Salmonella enterica Serovar Heidelberg isolated from broiler chickens. Antimicrob Agents Chemother.

[83] Milner KA, Bay DC, Alexander D, Walkty A, Karlowsky JA, Mulvey MR, Sharma MK, Zhanel GG (2020). Identification and characterization of a novel FosA7 member from Fosfomycin-resistant Escherichia coli clinical isolates from Canadian hospitals. Antimicrob Agents Chemother.

[84] Leclercq R, Courvalin P (1991). Bacterial resistance to macrolide, lincosamide, and streptogramin antibiotics by target modification. Antimicrob Agents Chemother.

[85] Wachino J, Arakawa Y (2012). Exogenously acquired 16S rRNA methyltransferases found in aminoglycoside-resistant pathogenic gram-negative bacteria: an update. Drug Resist Updat.

[86] McKessar SJ, Berry AM, Bell JM, Turnidge JD, Paton JC (2000). Genetic characterization of vanG, a novel vancomycin resistance locus of enterococcus faecalis. Antimicrob Agents Chemother.

[87] Courvalin P (2006). Vancomycin resistance in gram-positive cocci. Clin Infect Dis.

[88] Spigaglia P, Barbanti F, Mastrantonio P, Brazier JS, Barbut F, Delmée M, Kuijper E, R. Poxton I, on behalf of the European Study Group on (ESGCD) (2008). On behalf of the European study group on Esgcd. Fluoroquinolone resistance in Clostridium difficile isolates from a prospective study of C. difficile infections in Europe. J Med Microbiol.

[89] Walkty A, Boyd DA, Gravel D, Hutchinson J, McGeer A, Moore D, Simor A, Suh K, Taylor G, Miller M, Mulvey MR, Canadian Nosocomial Infection Surveillance Program (2010). Canadian nosocomial infection surveillance program. Molecular characterization of moxifloxacin resistance from Canadian Clostridium difficile clinical isolates. Diagn Microbiol Infect Dis.

[90] Mariam DH, Mengistu Y, Hoffner SE, Andersson DI (2004). Effect of rpoB mutations conferring rifampin resistance on fitness of mycobacterium tuberculosis. Antimicrob Agents Chemother.

[91] Zeef LA, Bosch L, Anborgh PH, Cetin R, Parmeggiani A, Hilgenfeld R (1994). Pulvomycin-resistant mutants of E.coli elongation factor Tu. EMBO J.

[92] Fu Z, Ma Y, Chen C, Guo Y, Hu F, Liu Y, Xu X, Wang M (2016). Prevalence of Fosfomycin resistance and mutations in murA, glpT, and uhpT in methicillin-resistant Staphylococcus aureus strains isolated from blood and cerebrospinal fluid samples. Front Microbiol.

[93] Yanat B, Rodríguez-Martínez JM, Touati A (2017). Plasmid-mediated quinolone resistance in Enterobacteriaceae: a systematic review with a focus on Mediterranean countries. Eur J Clin Microbiol Infect Dis.

[94] Yassine I, Rafei R, Osman M, Mallat H, Dabboussi F, Hamze M (2019). Plasmid-mediated quinolone resistance: mechanisms, detection, and epidemiology in the Arab countries. Infect Genet Evol.

[95] Pham TDM, Ziora ZM, Blaskovich MAT (2019). Quinolone antibiotics. Medchemcomm.

[96] Guillard T, Grillon A, de Champs C, Cartier C, Madoux J, Berçot B, Lebreil AL, Lozniewski A, Riahi J, Vernet-Garnier V, Cambau E (2014). Mobile insertion cassette elements found in small non-transmissible plasmids in Proteeae may explain qnrD mobilization. PLoS One.

[97] Troisfontaines P, Cornelis GR (2005). Type III secretion: more systems than you think. Physiology (Bethesda).

[98] Dale C, Young SA, Haydon DT, Welburn SC (2001). The insect endosymbiont Sodalis glossinidius utilizes a type III secretion system for cell invasion. Proc Natl Acad Sci U S A.

[99] Dale C, Jones T, Pontes M (2005). Degenerative evolution and functional diversification of type-III secretion systems in the insect endosymbiont *Sodalis glossinidius*. Mol Biol Evol.

[100] Dale C, Plague GR, Wang B, Ochman H, Moran NA (2002). Type III secretion systems and the evolution of mutualistic endosymbiosis. Proc Natl Acad Sci U S A.

[101] Brugirard-Ricaud K, Duchaud E, Givaudan A, Girard PA, Kunst F, Boemare N, Brehelin M, Zumbihl R (2005). Site-specific antiphagocytic function of the Photorhabdus luminescens type III secretion system during insect colonization. Cell Microbiol.

[102] Bolger AM, Lohse M, Usadel B (2014). Trimmomatic: a flexible trimmer for Illumina sequence data. Bioinformatics.

[103] Andrews S (2010). FastQC: a quality control tool for high throughput sequence data.

[104] Quinlan AR, Hall IM (2010). BEDTools: a flexible suite of utilities for comparing genomic features. Bioinformatics.

[105] Li H, Handsaker B, Wysoker A, Fennell T, Ruan J, Homer N, Marth G, Abecasis G, Durbin R, 1000 Genome Project Data Processing Subgroup (2009). 1000 genome project data processing subgroup. The sequence alignment/map format and SAMtools. Bioinformatics.

[106] Bankevich A, Nurk S, Antipov D, Gurevich AA, Dvorkin M, Kulikov AS, Lesin VM, Nikolenko SI, Pham S, Prjibelski AD, Pyshkin AV, Sirotkin AV, Vyahhi N, Tesler G, Alekseyev MA, Pevzner PA (2012). SPAdes: a new genome assembly algorithm and its applications to single-cell sequencing. J Comput Biol.

[107] Delcher AL, Bratke KA, Powers EC, Salzberg SL (2007). Identifying bacterial genes and endosymbiont DNA with Glimmer. Bioinformatics.

[108] Lowe TM, Eddy SR (1997). tRNAscan-SE: a program for improved detection of transfer RNA genes in genomic sequence. Nucleic Acids Res.

[109] Lagesen K, Hallin P, Rødland EA, Staerfeldt HH, Rognes T, Ussery DW (2007). RNAmmer: consistent and rapid annotation of ribosomal RNA genes. Nucleic Acids Res.

[110] Zhang H, Meltzer P, Davis S (2013). RCircos: an R package for Circos 2D track plots. BMC Bioinformatics.

[111] Alcock BP, Raphenya AR, Lau TTY, Tsang KK, Bouchard M, Edalatmand A, Huynh W, Nguyen ALV, Cheng AA, Liu S, Min SY, Miroshnichenko A, Tran HK, Werfalli RE, Nasir JA, Oloni M, Speicher DJ, Florescu A, Singh B, Faltyn M, Hernandez-Koutoucheva A, Sharma AN, Bordeleau E, Pawlowski AC, Zubyk HL, Dooley D, Griffiths E, Maguire F, Winsor GL, Beiko RG, Brinkman FSL, Hsiao WWL, Domselaar GV, McArthur AG (2020). CARD 2020: antibiotic resistome surveillance with the comprehensive antibiotic resistance database. Nucleic Acids Res.

[112] Galperin MY, Makarova KS, Wolf YI, Koonin EV (2015). Expanded microbial genome coverage and improved protein family annotation in the COG database. Nucleic Acids Res.

[113] Huerta-Cepas J, Szklarczyk D, Forslund K, Cook H, Heller D, Walter MC, Rattei T, Mende DR, Sunagawa S, Kuhn M, Jensen LJ, von Mering C, Bork P (2016). eggNOG 4.5: a hierarchical orthology framework with improved functional annotations for eukaryotic, prokaryotic and viral sequences. Nucleic Acids Res.

[114] Remm M, Storm CE, Sonnhammer EL (2001). Automatic clustering of orthologs and in-paralogs from pairwise species comparisons. J Mol Biol.

[115] Harris RS (2007). Improved pairwise alignment of genomic DNA.

[116] Larkin MA, Blackshields G, Brown NP, Chenna R, McGettigan PA, McWilliam H (2007). Clustal W and Clustal X version 2.0. Bioinformatics.

[117] Zhang Z, Schwartz S, Wagner L, Miller W (2000). A greedy algorithm for aligning DNA sequences. J Comput Biol.

[118] Tamura K, Stecher G, Peterson D, Filipski A, Kumar S (2013). MEGA6: molecular evolutionary genetics analysis version 6.0. Mol Biol Evol.

