# Supplementary material for: Genomic analysis of the nomenclatural type strain of the nematode-associated entomopathogenic bacterium Providencia vermicola
Source: BMC Genomics. 2021 Oct 2;22:708. doi: 10.1186/s12864-021-08027-w (PMC8487129; doi:10.1186/s12864-021-08027-w)
Supplement: Supplementary file 1 — Additional file 1. Sequences of Providencia vermicola DSM_17385 16S rRNA and hMLST marker genes. [file 12864_2021_8027_MOESM1_ESM.docx]

**Additional File 1.** Sequences of *Providencia vermicola* DSM_17385 16S rRNA and hMLST marker genes

>*Providencia_vermicola*_DSM_17385_*rrs*_gene_complete_coding_sequence

AGAGTTTGATCATGGCTCAGATTGAACGCTGGCGGCAGGCCTAACACATGCAAGTCGAGCGGTAACAGGGGAAGCTTGCTTCCCGCTGACGAGCGGCGGACGGGTGAGTAATGTATGGGGATCTGCCCGATAGAGGGGGATAACCACTGGAAACGGTGGCTAATACCGCATAATCTCTTAGGAGCAAAGCAGGGGAACTTCGGTCCTTGCGCTATCGGATGAACCCATATGGGATTAGCTAGTAGGTGGGGTAATGGCTCACCTAGGCGACGATCCCTAGCTGGTCTGAGAGGATGATCAGCCACACTGGGACTGAGACACGGCCCAGACTCCTACGGGAGGCAGCAGTGGGGAATATTGCACAATGGGCGCAAGCCTGATGCAGCCATGCCGCGTGTATGAAGAAGGCCCTAGGGTTGTAAAGTACTTTCAGTCGGGAGGAAGGCGTTGATGCTAATATCATCAACGATTGACGTTACCGACAGAAGAAGCACCGGCTAACTCCGTGCCAGCAGCCGCGGTAATACGGAGGGTGCAAGCGTTAATCGGAATTACTGGGCGTAAAGCGCACGCAGGCGGTTGATTAAGTTAGATGTGAAATCCCCGGGCTTAACCTGGGAATGGCATCTAAGACTGGTCAGCTAGAGTCTTGTAGAGGGGGGTAGAATTCCATGTGTAGCGGTGAAATGCGTAGAGATGTGGAGGAATACCGGTGGCGAAGGCGGCCCCCTGGACAAAGACTGACGCTCAGGTGCGAAAGCGTGGGGAGCAAACAGGATTAGATACCCTGGTAGTCCACGCTGTAAACGATGTCGATTTGAAGGTTGTTCCCTTGAGGAGTGGCTTTCGGAGCTAACGCGTTAAATCGACCGCCTGGGGAGTACGGCCGCAAGGTTAAAACTCAAATGAATTGACGGGGGCCCGCACAAGCGGTGGAGCATGTGGTTTAATTCGATGCAACGCGAAGAACCTTACCTACTCTTGACATCCAGAGAATTTAGCAGAGATGCTTTAGTGCCTTCGGGAACTCTGAGACAGGTGCTGCATGGCTGTCGTCAGCTCGTGTTGTGAAATGTTGGGTTAAGTCCCGCAACGAGCGCAACCCTTATCCTTTGTTGCCAGCGATTCGGTCGGGAACTCAAAGGAGACTGCCGGTGATAAACCGGAGGAAGGTGGGGATGACGTCAAGTCATCATGGCCCTTACGAGTAGGGCTACACACGTGCTACAATGGCGTATACAAAGAGAAGCGACCTCGCGAGAGCAAGCGGAACTCATAAAGTACGTCGTAGTCCGGATTGGAGTCTGCAACTCGACTCCATGAAGTCGGAATCGCTAGTAATCGTAGATCAGAATGCTACGGTGAATACGTTCCCGGGCCTTGTACACACCGCCCGTCACACCATGGGAGTGGGTTGCAAAAGAAGTAGGTAGCTTAACCTTCGGGAGGGCGCTTACCACTTTGTGATTCATGACTGGGGTGAAGTCGTAACAAGGTAACCGTAGGGGAACCTGCGGTTGGATCACCT

>*Providencia_vermicola*_DSM_17385_*fusA*_gene_complete_coding_sequence

ATGGCCCGTCAAACGCCCATAGCACGTTATCGTAATATCGGTATCAGTGCACACATCGACGCCGGTAAAACCACAACTTCTGAACGTATTCTGTTCTATACTGGTGTAAACCATAAAATTGGTGAAACTCACGAAGGTTCTGCAACAATGGACTGGATGGAGCAGGAGCAAGAGCGTGGTATCACTATCACATCTGCTGCGACTACTGCATTCTGGTCTGGTATGGCAAAACAGTATGAGCCACACCGTGTAAACATCATCGACACCCCGGGACACGTTGACTTCACAATCGAAGTAGAACGTTCTATGCGTGTTCTTGATGGCGCAGTAATGGTTTACTGTGCGGTTGGTGGTGTTCAGCCACAGTCTGAAACTGTATGGCGTCAGGCTAACAAATATAAAGTTCCACGTATTGCGTTCGTTAATAAAATGGACCGTATGGGTGCGAACTTCTTACGTGTTGTTGAGCAATTAAAAACACGTTTAGCAGCTAACGCAGTTCCACTGCAATTACCAGTCGGCGCAGAAGAGTCGTTCACTGGTGTTGTTGACTTGCTGAAAATGAAAGCAATCAAGTGGAGCGATGAAGACCAAGGCGTTACCTTCGAATACGAAGATATCCCTGCGAACATGCAAGAAGCAGCTGAAGAGTGGCACAACAACCTGATCGAAACCGCAGCAGAAGCATCAGAAGAACTGATGGAAAAATATCTGGGCGGTGAAGAACTGACTGAAGCAGAAATTAAAGCTGCATTACGTCAACGTGTTCTTGCAAGCGAAATTATCCTGGTTACCTGTGGTTCTGCATTTAAGAACAAAGGTGTTCAGGCGATGCTGGATGCAGTGATTGATTACTTACCTGCGCCAACAGATGTACCTGCAATTAATGGTATTCTGGATGACGGTAAAGACACTCCTGCAGAGCGTCACGCAAGTGATGATGAGCCATTCTCATCATTAGCATTTAAAATTGCAACCGACCCATTCGTTGGTAACTTAACGTTCTTCCGTGTTTACTCTGGTGTTGTTAACTCAGGTGACACAGTGCTGAACGCAGTTAAAGCGAAAAAAGAACGTTTTGGCCGTATCGTACAGATGCACGCTAACAAACGTGAAGAGATCAAAGAAGTTCGCGCTGGTGACATCGCGGCGGCAATCGGTCTGAAAGACGTAACGACAGGTGATACTTTATGTGCAGTTGATGCACCAATCATCCTAGAGCGTATGGAATTCCCAGAGCCAGTAATCTCTGTTGCAATTGAACCAAAAACGAAAGCTGACCAAGAAAAAATGGGTATCGCATTAGGCCGTCTGGCTCAAGAAGACCCATCATTCCGCGTATCAAGTGATGAAGAGACTAATCAGACTATCATCGCTGGTATGGGTGAATTGCACTTGGACGTTCTGGTTGACCGTATGCGTCGTGAATTTAAAGTTGAAGCGAACGTTGGTAAACCTCAAGTTGCTTACCGTGAAGCAATCACTGCTAAAGTGACTGACATCGAAGGTAAACACGCGAAACAGTCTGGTGGTCGTGGTCAGTACGGTCATGTCGTTATCGATATGTTCCCACTGAACAAAAACGATAAAGACGGTCTGCCAATGGACTACGAATTTGTCAACGAAATCAAGGGTGGTGTAATTCCAACTGAATACATCCCTGCGGTTGATAAAGGTATCCAAGAGCAGCTGAAATCTGGCCCATTAGCTGGCTACCCTGTTGTTAACATGGGTGTTCGTCTGCATTTCGGTTCTTACCATGATGTTGACTCCTCTGAACTGGCGTTTAAACTTGCGGCTTCAATCGCGTTTAAAGATGGCTTCAAAAAAGCTAAACCTGTTCTGCTTGAGCCAATCATGAAAGTCGAAGTGGAAACACCAGAAGACTACATGGGTGATGTTATTGGTGACCTGAACCGTCGTCGTGGTATGATTGAAGGTATGGATGACCTGCCTACTGGTAAAGTCGTTCGTGCACAAGTACCATTGTCCGAAATGTTCGGTTATGCTACTGACCTGCGTTCTCAGACACAAGGTCGTGCTTCATACTCTATGGAGTTCCTGAAGTACAATGAAGCACCAAACAACGTTGCACAGGCTGTTATCGAAGCTCGTAACGCTAAATAA

>*Providencia_vermicola*_DSM_17385_*gyrB*_gene_complete_coding_sequence

ATGTATATCGGTGATACAGACGATGGAACAGGTCTTCATCACATGGTTTTCGAGGTTGTCGACAATGCTATCGACGAAGCCCTCGCAGGCTTTTGTGACGATATCGTTATCACTATCCATGCCGATAACTCTATCTCCGTACAGGATGATGGACGTGGTATCCCAACAGGTATCCACGAAGAAGAAGGTGTCTCTGCCGCGGAAGTCATCATGACTGTTCTGCACGCAGGGGGGAAATTCGATGATAACTCCTATAAAGTTTCCGGCGGATTGCACGGTGTAGGGGTATCAGTTGTTAACGCCTTATCTGAAAAACTGGAATTAGTCATCAAACGTGATGGCAAAGTTCACGAACAAATCTACAAACACGGTGAACCACAAGGCCCTCTGTCCGTTGTTGGTGAAACTGACCAAACAGGGACTCGCGTCCGCTTTTGGCCAAGTATGGATACCTTTAAAGGCGTCACTGAGTTCGAGTATGACGTATTAGCGAAGCGTTTACGTGAGTTATCATTCTTAAACTCGGGCGTTTCAATCAAACTCATCGATAAGCGAGATGGAAAAGAAGACCATTTCCATTATGAAGGTGGTATCAAAGCGTTCGTTGAGTATTTAAGCCGTAACAAAACCCCAATTCACCCATCCGTATTCTATTTTTCAACCGAAAAAGACGGTATTGGCGTTGAAGTGTCAATGCAGTGGAATGATGGTTTCCAAGAAAACGTGTACTGCTTTACCAACAACATTCCACAACGTGACGGTGGTACACACTTAGCGGGTTTCCGTGCCGCAATGACACGTACACTGAACAACTACATGGAAAAAGAAGGTTATCAGAAGAAAAGCAAAGTCAACGCAACGGGTGACGATGCGCGTGAAGGTCTGATCGCCGTGATTTCTGTGAAAGTACCCGATCCGAAATTCTCATCACAAACCAAAGAAAAACTGGTGTCATCCGAAGTGAAAACCGCGGTTGAAACGATGATGAATGAAAAACTAGTTGAGTATTTACTGGAAAACCCAAATGACGCCAAAATCGTCGTTGGGAAAATCATTGATGCCGCGCGTGCCCGTGAAGCTGCACGTAAAGCCCGTGAAATGACGCGACGTAAAGGTGCATTAGACTTAGCAGGCTTGCCAGGTAAACTGGCTGACTGCCAAGAACGCGACCCTGCGTTATCTGAACTGTACCTTGTGGAAGGGGACTCTGCGGGCGGCTCTGCAAAACAAGGCCGTAACCGTAAAAACCAGGCTATCTTGCCACTGAAAGGTAAAATTCTTAACGTTGAAAAAGCGCGTTTTGATAAAATGCTTTCTTCTCAAGAAGTTGCAACCCTTATTACGGCCTTAGGTTGTGGTATTGGTCGCGATGAATATAACCCAGACAAACTGCGTTATCACAGCATCATTATCATGACGGATGCCGACGTCGATGGTTCGCATATTCGTACGTTACTGTTGACCTTCTTTTATCGTCAAATGCCTGAAATTGTTGAGCGCGGCCATATCTTTATTGCTCAGCCACCACTGTACAAAGTGAAACGTGGTAAACAAGAACAGTACATTAAAGATGACGATGCAATGGACGATTACCTGATTTCTATCGCCCTTGATGGTGCAGAGCTGCATTTAAGTGCAGATGCCCCAGCCATGAAAGGTGAGGAATTAGAAAAGCTGGTTGTCGAATACAACGCGGCTCATCGTATTATTCGACGTCTTGAGCGCCTTTACCCACAAGCTTTACTCAATAGTTTGGTTTATCAGTCTACCCTGACTGAAGATGATCTAAAAACCAAAGAAAAAGTAGAAGAGTGGGCAAAAACTCTTGTTCAGCGCTTAACGGATAACGAACAATTCGGTAGCACCTACAGCTACACCATCCATGAAAACCGTGAACGTCAATTATTTGAACCCACTATCCGTATTCGCACCCACGGTGTTGATACTGATTACAACCTCGATTTTGACTTTGTTCATGGTAGTGAATACCGCCGTATCACACACTTAGGTGATATTATTGGTGGGCTGATTGAAGAAGGTGCTTACATTCAACGTGGTGAACGCCGTCAAGACATTAACAACTTCGAAGAAGCACTGGCTTGGTTAACACGTGAATCACGTCGTGGCCTATATGTACAGCGCTATAAAGGCCTTGGTGAAATGAACCCAGAACAACTTTGGGAAACCACCATGAACCCTGAAACACGTCGTATGATGCGTGTGACGGTGAAAGACGCAATCGCAACAGATTTACTCTTCACCACATTAATGGGTGATGCAGTTGAACCGCGTCGTGCCTTTATTGAAGAAAATGCATTAAAAGCTGCAAATATCGATATTTAA

>*Providencia_vermicola*_DSM_17385_*ileS*_gene_complete_coding_sequence

ATGCGTGGCGATCTCGCTAAACGCGAACCACAGATGTTAGAGCGCTGGTACAAAGAAGGTTTGTATCAGGCAATCCGTAAAGCAAAATCGGGCAAGAAAACTTTTATTCTGCACGATGGCCCTCCATATGCTAACGGCAGTATTCATATTGGTCACTCAGTTAACAAAATTCTCAAAGATATTATTATTAAATCCAAAGGGTTAGCGGGTTATGATTCCCCGTATATTCCTGGTTGGGATTGCCATGGCTTACCGATTGAACACAAAGTTGAACAAATCGTGGGTAAACCAGGTGAAAAAGTTTCCGCCGCTGAATTTCGTGCACAATGCCGTCAATATGCAAAAGAGCAAATTGAAGGCCAAAAAGCTGACTTTATGCGTCTCGGTGTGTTAGGTGAGTGGGATAAACCTTACTTAACCATGGACTTCAAAACCGAAGCCCACATCATCAGAGCGTTAGCAAAAACCATTGCAAACGGCCATTTAGTGAAAGGCGCTAAGCCTGTTCATTGGTGTACAGCATGTGGTTCTTCATTAGCTGAAGCTGAAGTTGAGTATTATGACAAAACTTCCCCATCTATCTACGTTCGTTTCCCAGCAGTAGATAGCAAAGCGGTGTGTGAAAAATTCGGGGTTACGAGTGATAAAACGCCATCACTGGTTATCTGGACAACAACACCGTGGACATTGCCTGCTAACCGCGCAATCTCGTTGAATCCTGAATTTAAATACAATTTGGTACAAGCGAATGGCGAATTAGTCATTTTAGCGGCTGACCTTGTTGAAGATGTAATGAAAACTGTTGGTATCGACGAATGGGCAGTTTTAGGCGAATGTGAAGGCGCTGCGCTTGAACTGTTGCGCTTTGAGCACCCATTTATGGGCTTTGATGTTCCTGCAATCTTAGGTGACCATGTTACATTAGATGCAGGTACAGGTGCTGTTCATACTGCGCCAGGCCATGGTCCCGAGGACTATGTGGTTGGCCAAAAATACGGTTTAGAGACAGCAAACCCTGTTGGGCCAGACGGTTGTTTCTTAGCCAATACCTATCCAACATTGGATGGTGTGTTCATTTTTAAAGCTAACGATTTGATTGTTGAGCTGTTAAATGAAAAAGACGCGTTGCTGTATAAAAAAGCAATCACCCACAGCTATCCATGTTGCTGGCGTCATAAAACCCCCGTTATTTTCCGTGCAACACCACAATGGTTTATTGGCATGGATAAAAATGGCTTACGCGAACAATCATTGAAAGAAATTGATATCGTTCAGTGGATCCCTGGTTGGGGGCGTGCACGTATTGAATCAATGGTTGAAAACCGCCCTGACTGGTGTATCTCTCGCCAGCGTACTTGGGGGACGCCGATGTCTTTGTTTGTTCATAAAGATACCGAAGAGCTGCATCCTCGTACATTAGAATTAATGGAAGAAGTCGCTAAACGCGTTGAAGTTGACGGTATTCAAGCATGGTGGGATCTCGATCCAGCTGAATTACTGGGTGATGAAGCTGAAACCTACCGTAAAGTGCCAGATACACTAGACGTCTGGTTTGACTCAGGGTCTACACACTTTGCCGTGGTTGATGCACGTCCTGAATTCCATGGTAATTCAGCTGACATGTACTTGGAAGGCTCTGATCAACACCGTGGCTGGTTCATGTCCTCATTGATGTTATCAACAGCAATGAAAGGTAAAGCGCCATACCGCCAAGTATTAACGCATGGTTTCACCGTTGATGGCCAAGGCCGTAAAATGTCTAAATCATTAGGCAATACGGTAAGTCCACAAGATGTCATGAATAAACTGGGTGCAGATATTCTGCGTCTATGGGTTGCTTCTACGGATTATACTGGCGAAATCGCGGTTTCTGATGAAATCTTAAAACGTGCAGCGGATTCATATCGCCGTATTCGTAATACTGCCCGCTTCTTATTGGCGAACCTTAACGGTTTCAATCCAGAAACAGACATGGTGAAACCTGAAGATATGGTGGTGTTAGACCGTTGGGCGGTTAGCCGTGCGCTTGAAGCACAACAAGAAATCACCAAAGCGTATGATGAATATGATTTCCTTTCCGTTATTCAGCGTTTAATGCATTTCTGCTCTATCGAAATGGGTTCGTTTTATCTGGATATCATTAAAGACCGTCAGTACACAGCGAAAGGCGATAGCTTAGCGCGTCGTAGCTGCCAAACAGCTCTGTTCCATATCGTTGAAGCATTAGTACGTTGGATAGCCCCAGTGCTTTCTTTCACTTCAGACGAAATCTGGAATGAATTGCCAGGAAAACGTGCTCAGTTCGTTCTAACCGAAGAATGGTATAACGGCTTATTTGGCTTAGATGAGTCAAATGAAATGAATAACAGCTTCTGGTCTGAACTGCTGGCGGTTCGTGGTGAAGTGAATAAAGTGTTAGAGCAAGCGCGTACAGATAAACACATTGGTGGCTCACTAGAGGCTGCGGTGACGTTATATGCAGATAAAGACTTAGCAAACAAACTGCAAAGTTTAGGTGATGAACTGCGTTTCGTTCTGCTGACTTCTCAAGCGACTGTCGCGGATATTGCAACGGCGTCTGCTGATGCGCAAGAGAGCGAGCTAAAAGGCCTTAAAATTGCCTTTAGTAAAGCGGAAGGCGAAAAATGCCCACGTTGCTGGCATTATGCGAGCGATATCGGCTCATCAAGCGAGCAACCAGAAATTTGTGGTCGCTGTGTGACGAACGTAGCCGGTGACGGCGAATTGCGTAAGTTTGCTTAA

>*Providencia_vermicola*_DSM_17385_*lepA*_gene_complete_coding_sequence

TTGAAAATCAATAATATAAGAAACTTTTCTATCATTGCTCATATCGACCATGGCAAATCCACGTTATCTGATCGTATTATTCAGATTTGTGGTGGTTTAACGGATCGCGAAATGGCAGCGCAGGTTTTAGATTCAATGGATCTGGAACGTGAGCGTGGAATTACCATCAAAGCACAGAGCGTTACGCTCGATTATAAAGCCTCTGACGGTGAAACCTACCAATTAAACTTTATCGACACACCAGGCCACGTTGACTTCTCTTATGAAGTTTCGCGTTCTCTTGCGGCGTGTGAAGGGGCATTGTTAGTTGTCGATGCAGGGCAAGGTGTTGAAGCGCAAACATTAGCTAACTGCTATACAGCGATTGAAATGGATTTAGAAGTCGTTCCTGTTTTAAACAAGATTGACTTGCCAGCGGCAGACCCAGAGCGCGTTGCTGATGAAATTGAAGACATTGTAGGCCTTGATGCCCACGATGCCGTTCGCTGTTCTGCAAAAACGGGTATTGGTGTTCAAGATGTGATTGAACGCCTTGTTAAGGAGATCCCACCCCCAGTTGGCGATGCGGATGCCCCACTACAAGCACTGATTATTGACTCTTGGTTTGATAACTATTTAGGCGTTGTTTCGTTGATTCGTATTAAAAACGGGTCACTGAAAAAAGGCGATAAAATTAAAGTCATGAGTACAGGACAGGTCTATAACGCTGATCGCCTTGGTATTTTCACACCTAAACAAATCGACCGTGATGTACTTAGTTGTGGCGAAGTAGGCTGGTTAGTCTGCGCAATTAAAGACATCACTGGTGCGCCAGTAGGGGATACCTTAACGGGCGCTCGTAATCCTGCGGAAAAAGCACTACCAGGCTTTAAAAAAGTAAAACCACAGGTTTATGCAGGTCTATTCCCTGTTAGTTCTGATGATTATGAAGGTTTCCGTGATGCGCTAGGTAAGCTTAGCTTAAATGATGCCTCTTTATTCTATGAGCCTGAAACATCGACTGCTTTAGGTTTCGGTTTCCGTTGTGGTTTCCTTGGCTTATTGCATATGGAAATCATTCAAGAACGTTTAGAACGTGAATACGATCTTGACCTGATCACTACGGCACCAACGGTTGTATATGAAGTTCAACAAACCAACGGTGAGATTGTTTATGTGGATAGCCCATCAAAGCTACCAGCATTAAACAATATTGAAGAACTGCGTGAGCCAATTGCAGAATGTCACATGTTAATGCCTAAAGAATACTTAGGTAACGTGATTACACTTTGTATTGAGAAACGTGGCGTTCAAACCAATATGGTCTATCATGGTAATCAAGTTTCCTTGACATATGACATTCCTATGTCTGAAGTCGTACTGGATTTCTTCGACCGTTTGAAATCAACATCTCGTGGTTATGCGTCATTGGATTATGCATTTAAGCGTTTTCAGCCATCAGATATGGTTAGGGTTGATGTGCTTATCAACAACGAACGTGTTGATGCATTAGCATTGATTACACACCGCGCAAACTCACAATATCGTGGTCGTGAATTGGTCGAGAAAATGAAAGAACTGATCCCTCGCCAGCAGTTTGATATTGCTATCCAGGCCGCGATTGGTAACCATATTATTGCTCGTTCTACAGTTAAACAGTTGCGTAAAAACGTACTTGCCAAATGTTATGGCGGTGACGTTAGCCGTAAGAAGAAACTGTTGCAGAAGCAAAAGGATGGTAAGAAACGTATGAAACAAGTGGGTAACGTTGAGCTACCACAAGAAGCGTTCCTTGCTATATTGCATGTTGGTAAAGACAACTAA

>*Providencia_vermicola*_DSM_17385_*leuS*_gene_complete_coding_sequence

ATGCAAGAACAATATCGTCCAGAAGATATAGAGCCTAAAGTACAGCGTCACTGGGATGAAAAAGCAACTTTCAAAGTGACTGAAGACAACAGCAAAGAAAAATACTACTGCCTGTCCATGCTACCTTACCCTTCTGGTCGACTACACATGGGCCACGTACGTAACTACACCATGGGTGACGTTATCTCTCGTTACCAACGTTTGTTGGGGAAAAACGTTCTCCAACCAATTGGTTGGGATGCGTTTGGCCTACCTGCTGAAGGTGCTGCGGTTAAAAATAACACGGCTCCTGCGCCATGGACTTATGCCAACATTGATTACATGAAAAGCCAGCTTAAAATGCTCGGCTTCGGTTACGATTGGGATCGCGAAGTTACGACTTGTACCCCTGAATATTACCGTTGGGAACAATGGTTCTTCACTAAGCTGTATGAAAAGGGTTTAGTGTATAAAAAAACCTCAGCGGTCAACTGGTGTCCACATGACTTAACCGTTCTGGCTAATGAGCAAGTCATTGATGGGTGCTGCTGGCGTTGTGATACCCCAGTTGAACGCAAAGAAATCCCACAATGGTTCATCAAGATTACTGACTACGCAGAAGAGTTGCTCAACGATCTGAACAAACTGGAAGATTGGCCTGAACAAGTTAAAACGATGCAACGCAACTGGATCGGTCGCTCCGAGGGCACTGAGATCACCTTTAATGTTGCTGATCGCGATGAAACCTTAACGGTTTATACCACTCGCCCTGACACTTTTATGGGTGCGACTTATGTTGCCGTTGCCGCAGGTCACCCTTTAGCAAAAGAAGCCGCTACGAATAATCCTGAATTAGCGCAATTTATTGATGAATGCCGTAACACCAAAACCGCAGAAGCGGACATGGCAACGATGGATAAAAAAGGGATGGCAACAGGCTTATTCGTTGTACATCCATTGACTCAAGAAAAACTGCCAATTTGGGTCGCAAACTTTGTATTAATGGAATACGGTACTGGCGCAGTTATGGCTGTTCCTGCTCACGACCAACGTGACTGGGAGTTTGCTCATAAATACAATCTGCCAATTAAAGCCGTCATTGCTGATGCAGAAGGCAACGAGCCTGATTTATCCCAAGAAGCCATGACGGAGAAAAACTCACTGATTAATTCAGGTGAATTCAGTGGGTTAGACCATCAGGCTGGTTTCAATGCGATTTCAGACAAACTTGTTGCACTGGGTGCAGGCCAACGAAAAGTCAACTATCGCTTACGTGACTGGGGTGTTTCACGTCAACGTTACTGGGGCGCGCCAATCCCAATGGCAACATTGGAAGACGGCACTGTCGTTCCTGTTCCAGAAGATCAACTGCCTGTGATTTTGCCTGAAGATGTTGTAATGAACGGCATTACCAGCCCGATTAAAGCAGATCCTGAGTGGGCGAAAATGACCATTAATGGTCAACCTGCACTACGTGAAACAGATACCTTCGATACCTTTATGGAATCTTCTTGGTACTACGCACGTTATACTTGCCCTCAATATGATGACGGTATGTTAGACCCAGCTGCAGCTAACTACTGGTTACCTGTAGACCAATATATCGGCGGTATCGAACACGCCATCATGCACTTAATGTATTTCCGTTTCTTCCACAAATTGATGCGTGATGCAGGGCTGGTCAACTCCGATGAACCTGCAAAACGTCTGCTGTGCCAAGGCATGGTACTTGCTGATGCATTCTATTATACAGGAAGCGATGGACAACGTGTTTGGGTTTCCCCTGCTGATGCTATTGTTGAGCGTGATGACAAAAACCGTATTACTAAAGCTGTAGACAACGAAGGCCATGAACTGGTTTATACTGGTATGAGCAAAATGTCTAAGTCTAAAAATAACGGTATTGACCCACAATTAATGGTCGAAAAATACGGCGCAGATACGGTTCGTTTATTCATGATGTTCGCAGCACCGCCTGAGCTAACCCTTGAATGGCAAGAGTCTAGCGTTGAAGGTGCCAACCGCTTCGTTCGCCGTGTTTGGCGTTTAGTGCATGAACATTCTCAAAAAGGCGCAACGTCTCCTTTAGATATCAGCGCATTAACAACAGAGCAAAAAGACTTACGCCGTGACCTACACAAAACAATCGCTAAGGTCTCTGATGATTTTAGTCGCCGTTATGCATTTAACACCGCAATTGCCGCGATCATGGAGTTCTTGAACAAATTAGTTCGTGCTCCACAAGAAACCGAACAAGACCGCGCATTAGTCCAAGAGTCTCTCGAAGCCATTACACTGATGCTGTCACCCATTATTCCACATGCTTGCTTTGAAATGTGGAAAGCTTTGGGCCATCAAACAGACATCGATTTCGCACAATGGCCTGTTGCTGATGAAAAAGCCATGATTGATGACACTAAGTTGGTTGTCGTTCAAGTCAATGGTAAGGTTCGTGGTCGTATTACCGTTCCAGCCGATGCGACACAAGAATTTGTCTTAGAAATGGCACAACAAGAGCCTAGCGTGTCGAAATATCTTGAAGAGGTAAGTATACGCAAAGTAATCTATGTTCCAGGCAAATTGCTTAACCTTGTTGTAGGTTGA
