# Supplementary material for: Genomic analysis of the nomenclatural type strain of the nematode-associated entomopathogenic bacterium Providencia vermicola
Source: BMC Genomics. 2021 Oct 2;22:708. doi: 10.1186/s12864-021-08027-w (PMC8487129; doi:10.1186/s12864-021-08027-w)
Supplement: Supplementary file 2 — Additional file 2. Sequences of Providencia vermicola DSM_17385 rMLST marker genes. [file 12864_2021_8027_MOESM2_ESM.docx]

**Additional File 2.** Sequences of *Providencia vermicola* DSM_17385 rMLST marker genes

>*Providencia_vermicola*_DSM_17385_*rpl*A_gene_complete_coding_sequence

ATGGCTAAACTGACTAAGCGCATGCGCAATATCCGTGAAAAAGTTGATGCTACTAAACAGTATGACATCGCTGAAGCCGTTGCACTGCTGAAAGAATTAGCGACAGCTAAATTCGTTGAAAGCGTTGACGTTGCTGTTAACCTGGGCATCGATGCTCGTAAATCTGACCAGAACGTTCGCGGTGCAACTGTACTGCCACACGGTACTGGCCGTTCAGTTCGCGTTGCTGTGTTCGCACAAGGCGCAAACGCTGAAGCTGCTAAAGCTGCTGGCGCTGAACTGGTTGGTATGGAAGATCTGGCTGATAAAATCAAAGCTGGCGAAATGGACTTTGACGTTGTTATTGCTTCTCCAGATGCAATGCGCGTTGTTGGCCAATTAGGTCAAGTTTTAGGCCCACGTGGTCTGATGCCAAACCCGAAAGTAGGTACTGTAACACCTAACGTTGCTGAAGCGGTTAATAACGCTAAAGCAGGTCAGGTTCGTTACCGTAATGACAAAAACGGAATCATCCACACCACTATCGGTAAAGTTGATTTCGACGCTGACAAACTGAAAGAAAACTTAGAAGCACTGCTGATCGCGCTGAAAAAAGCAAAACCAGCTTCTGCTAAAGGTGTTTTCATCAAGAAAGTTAGCCTGTCTACCACTATGGGTGCAGGTGTTGCTGTTGACCAAGCTGGTCTGTCAGCGACAGTTTAA

>*Providencia_vermicola*_DSM_17385_*rpl*B_gene_complete_coding_sequence

*ATGGCAGTTGTTAAATGTAAACCTACGTCTCCGGGCCGTCGCCACGTAGTTAAAGTGGTTAACCCTGAGCTGCATAAGGGTAAACCTTATGCTCCGCTGTTAGAAAAAAACAGCAAATCCGGTGGTCGTAACAACAATGGCCGTATCACTACCCGTCACATCGGTGGTGGCCATAAGCAGCATTATCGTTTAGTTGACTTTAAACGCAACAAAGATGGTATCCCTGCTGTTGTTGAGCGTTTGGAATATGATCCAAACCGTTCTGCGAACATCGCACTGGTTCTATATAAAGACGGTGAACGTCGTTATATTCTTGCACCAAAAGGCCTGAAAGCTGGTGACCAGATTCAATCTGGTGTTGATTCAGCAATCAAAGCTGGTAATGCAATGCCAATGCGCAACATCCCTGTTGGTTCTACAGTTCACAACATTGAACTGAAACCAGGTAAAGGTGGTCAGTTAGCGCGTTCAGCAGGTACTTACGCTCAAATCGTTGCTCGTGATGGTGCTTATGTAACATTACGTCTGCGTTCTGGTGAAATGCGTAAAGTATTAGCAGATTGCCGTGCAACCTTAGGTGAAGTTGGTAACGCTGAACATATGTTACGTGTTCTCGGTAAAGCTGGTGCTAGCCGCTGGCGTGGTATTCGTCCTACCGTTCGCGGTACTGCGATGAACCCAGTAGACCATCCACATGGTGGTGGTGAAGGCCGTAACTTTGGTAAACACCCAGTAACACCATGGGGCGTTCAGACCAAAGGTAAGAAAACTCGTAGCAACAAGCGTACTGATCAATTCATCGTACGTCATCGTTCTAAAAAATAA*

>*Providencia_vermicola*_DSM_17385_*rpl*C_gene_complete_coding_sequence

ATGATTGGTTTAGTCGGTAAGAAAGTGGGAATGACACGTATCTTCACTGAAGATGGTGTTTCTATCCCTGTAACTGTTATCGAAATTGAAAATAACCGCGTTACTCAGGTTAAAACTGCAGAAACTGACGGTTACAATGCAATTCAGGTTACTACTGGTAGCAAAAAAGCTAACCGTGTAACTAAACCTGAAGCAGGTCATTTCGCTAAAGCTGGCGTTGAAGCTGGCCGTATTCTGCGCGAATTCCGTACTGAAGAAGGTGCTGAATTTACTGCAGGTCAAAGCATTAGCGTTGAAATTTTCGCTGACGTTAAAAAAGTCGACGTTACTGGTACATCTAAAGGTAAAGGTTTTGCTGGCACAGTTAAACGCTGGAATTTCCGTACTCAAGATGCTACTCACGGTAACTCCTTGTCACACCGTGTTCCGGGTTCTATCGGTCAGAACCAGACTCCGGGCAAAGTGTTCAAAGGCAAGAAAATGGCAGGCCAACTGGGTAATGAACGTGTAACTGTTCAAAGCTTAGATGTAGTACGTGTTGACGCTGAGCGCAACCTGCTGCTGGTCAAAGGTGCTGTTCCAGGAGCAACTGGTAGCAACCTGATCGTAAAACCGGCTGTCAAGGCTTAA

>*Providencia_vermicola*_DSM_17385_*rpl*D_gene_complete_coding_sequence

ATGGAATTGGTAATGAAAGACGCGCAAAGCGCGCTGACTGTTTCCGAAACTACCTTCGGGCGTGATTTCAATGAAGCGCTTGTTCACCAAGTAGTTGTTGCGTATGCAGCTGGTGCTCGTCAAGGTACTCGTGCTCAGAAAACTCGTGCTGAAGTTTCTGGTTCAGGTAAAAAACCATGGCGTCAAAAAGGCACAGGCCGTGCACGTTCTGGTTCAATCAAGAGCCCAATCTGGCGCTCTGGTGGTGTTAGCTTCGCAGCTAAACCACAGGACCACAGTCAAAAAGTTAATAAAAAGATGTACCGTGGTGCTTTAAAAAGCATCCTCTCTGAGTTGGTACGTCAAGATCGTCTGATCGTTGTCGAACAGTTCTCTGTTGAAGCACCTAAAACTAAGTTGCTGGCACAGAAATTGAAAGATATGGCTCTGCAAGATGTTCTGATCATCACTGCTGAATTAGATGAAAATCTGTTCTTAGCAGCACGTAACCTGTACAAGGTTGACGTTCGTGATGCAGCAGGTATCGACCCAGTTAGCCTGATTGCTTTCGACAAAGTGGTTATGACTGCTGATGCTGTGAAGCAAGTTGAGGAGATGCTGGCATGA

>*Providencia_vermicola*_DSM_17385_*rpl*E_gene_complete_coding_sequence

ATGGCGAAACTGCATGATTACTATAAAGACGAAGTTGTTAATAAACTCATGACTGAGTTTAGTTACTCTTCTGTCATGCAAGTCCCTCGGGTCGAGAAGATCACCCTGAATATGGGTGTTGGTGAAGCGATTGCTGATAAAAAACTGCTGGATAACGCAGCAGCTGATTTAACAGCTATCTCTGGTCAAAAACCATTGATCACCAAAGCACGCAAATCTGTTGCAGGCTTCAAAATCCGTCAGGGCTATCCAATCGGCTGTAAAGTAACCCTGCGTGGCGAACGCATGTGGGAGTTCCTTGAGCGTCTGATTTCTATTGCTGTACCACGTATTCGTGACTTCCGTGGCTTGTCCGCTAAGTCTTTCGATGGTCGCGGTAACTACAGCATGGGTGTTCGTGAACAAATCATCTTCCCTGAAATCGATTACGATAAAGTGGATCGCGTACGTGGTTTAGATATTACTATCACCACTACTGCGAAATCAGATGACGAAGGTCGCGCACTGTTAGCAGCGTTCAACTTCCCGTTCCGCAAGTAA

>*Providencia_vermicola*_DSM_17385_*rpl*F_gene_complete_coding_sequence

ATGTCTCGTGTGGCAAAAGCACCCGTCGTCATTCCTGCCGGCGTAGAGGTAAAACTCAACGGTCAGGTTATTTCGATTAAGGGTAAAAACGGCGAGCTTACTCGTACTATCCATAATGCAGTTGAAGTTAAACATGAAGATGGCCATTTAACTTTCGGACCACGTGATGGTTTTGAAGATGCATGGGCACAAGCGGGTACTACTCGTTCACTGTGCAATGCAATGGTTGTTGGTGTTACCGATGGCTTCACTAAAAAGCTTCAACTGGTTGGTGTTGGTTACCGTGCAGCAATCAAAGGCAATGCTGTTAGCTTGTCTTTAGGTTTCTCGCATCCGGTTGAACACGCACTGCCAGCAGGCATTACTGCTGAATGCCCAACACAAACTGAAATCGTACTGAAAGGTGCGGATAAGCAAGTGATTGGTCAAGTTGCAGCAGAACTGCGTGCTTACCGTCGTCCTGAGCCTTACAAAGGTAAAGGTGTTCGTTACGCCGATGAAATCGTGCGTACCAAAGAGGCTAAGAAGAAGTAA

>*Providencia_vermicola*_DSM_17385_*rpl*I_gene_complete_coding_sequence

ATGCAAGTTATTCTGCTTGATAAAGTAGCTAACCTAGGTAGCCTGGGTGATCAGGTTAACGTTAAATCGGGCTATGCTCGTAACTTCTTAGTTCCACAGGGCAAAGCTGTTCCTGCAACTAAGAAAAACATCGAATTCTTCGAAGCTCGCCGCGCTGAACTGGAAGCTAAATTAGCTGACGTTCTGGCAGCAGCACAGGCTCGTGCAGCAGCTGTTACTGCACTGGGTTCTGTTACTTTAGCTTCTAAAGCTGGTGACGAAGGTAAACTGTTCGGTTCAATCGGTACTCGTGACATCGCTGATGCAGTGACTGCAGCTGGCGTTAAAATCGCGAAAAGCGAAGTTCGCCTGCCAAACGGCGTTCTGCGTACTACTGGTGACCACGAAGTTCACTTCCAGTTACACAGCGATGTTTTCGCAGAGCTGAACGTTATCATCGTTGCTGAGTAA

>*Providencia_vermicola*_DSM_17385_*rpl*J_gene_complete_coding_sequence

ATGGCACTAAATCTTCAAGACAAACAAGCGATTGTTGCTGAAGTCAGCGAAGTAGCCAAAGGCGCGCTTTCTGCAGTTGTTGCTGATTCACGCGGCGTAACTGTAGCAAAAATGACTGAACTGCGTAAAGCAGGTCGCGAAGCTGGCGTTTATATCCGTGTTGTTCGTAACACACTGATTCGCCGTGCTGTTGAAGGTACTTCTTATGATGTACTGAAAGACGCGTTTGTTGGTCCAACCTTAATTGCTTTCTCTAACGAACACCCGGGCGCTGGCGCTCGTCTGTTCAAAGAGTTCGCGAAAGCGAATCCAGCATTTGAGATTAAAGCTGCGGCCTTTGAAGGTGAGTTAATCCAAGCGAAAGATATCGATCGTCTGGCAACTCTCCCAACTTACGATGAAGCAATCGCACGCCTGATGGCAACCATGAAAGAAGCCGCTGCCGGCAAATTGGTTCGTACTCTGGCTGCTCTGCGCGATCAGAAAGAAGCTGCATAA

>*Providencia_vermicola*_DSM_17385_*rpl*K_gene_complete_coding_sequence

ATGGCTAAGAAAGTTCAAGCCTACGTTAAGCTGCAAGTTGCGGCTGGTATGGCGAACCCAAGTCCACCAGTTGGTCCAGCTCTGGGTCAACAAGGTGTTAACATCATGGAATTCTGTAAAGCGTTCAACGCAAAAACAGACAGCCTGGAAAAAGGTTTACCAATTCCTGTTGTTATTACTGTTTACGCAGACCGTTCTTTCACTTTCGTTACCAAAACTCCACCTGCAGCAGTTCTGCTGAAAAAAGCAGCTGGCGTTAAGTCTGGTTCAGGCAAGCCTAACAAAGACAAAGTTGGTAAAGTGACCTCTGCTCAGATTCGCGAAATCGCTGAAACTAAAGCTGCGGACATGACTGGTGCTGACGTTGACGCAATGATGCGTTCAATCGAAGGTACTGCTCGTTCCATGGGCCTGGTTGTGGAGGACTAA

>*Providencia_vermicola*_DSM_17385_*rpl*L_gene_complete_coding_sequence

ATGTCTATCACTAAAGACCAAATCCTTGATGCAGTTGCAGAAATGTCTGTAATGGACGTTGTTGAACTGATTACTATGATGGAAGAAAAATTCGGCGTTTCTGCTGCTGCAGCTGTTGCTGTTGCTCCAGGTGCTGCTGCTGAAGCTGCTGAAGAAAAAACTGAATTCGACGTTGTTCTGTCTGCTATCGGCGGTAACAAAGTTGCAGTTATCAAAGCAGTTCGTGGCGCAACTGGTCTTGGCCTGAAAGAAGCTAAAGACATGGTAGAATCAGCTCCAGCGACCATCAAAGAAGGCGCAAGCAAAGACGAAGCTGAAACTCTGAAGAAAGCTCTTGAAGAAGCAGGCGCTTCTGTTGAACTTAAATAA

>*Providencia_vermicola*_DSM_17385_*rpl*M_gene_complete_coding_sequence

ATGAAAACTTTTACAGCTAAACCAGAGACCGTAAAACGCGACTGGTACGTTGTTGATGCAGATGGCAAAACTTTAGGCCGTCTTGCAACAGAAATTGCTAGCCGTCTGCGCGGTAAGCATAAAGCGGAATACACTCCGCACGTGGATACTGGTGATTACATCATCGTTCTGAATGCAGAAAAAATTGCTGTTACCGGCAAAAAACGCGAAGACAAAATCTACTATCGCCACACTGGCCATGTAGGTGGAATCAAGCAAGCAACTTTCGAAGAAATGATTGCTCGCAATCCTGAGCGTGTGCTTGAAATCGCGGTTAAAGGCATGTTGCCAAAAGGACCTCTGGGTCGTGCAATGTACCGTAAACTGAAAGTTTACGCAGGTAATGAGCACAACCACGCGGCTCAACAACCGCAAGTTCTTGACATTTAAT

>*Providencia_vermicola*_DSM_17385_*rpl*N_gene_complete_coding_sequence

ATGATCCAAGAACAGACTATGCTGAACGTGGCCGACAACTCCGGTGCACGTCGCGTAATGTGTATCAAGGTTCTAGGTGGCTCGCACCGTCGCTATGCACATGTAGGCGACATCATTAAAATTACTGTTAAAGAAGCAATTCCACGCGGTAAAGTTAAAAAGGGTGATGTCCTGAAAGCGGTAGTGGTGCGCACCAAGAAGGGTGTACGTCGCCCTGACGGTTCTGTCATTCGCTTCGATGGTAATGCTTGTGTGTTATTAAACAATAACAGCGAGCAAGTAATCGGTACGCGTATTTTTGGGCCGGTAACTCGTGAACTTCGTAATGAGAAGTTTATGAAAATTATCTCTCTGGCACCTGAAGTACTCTAA

>*Providencia_vermicola*_DSM_17385_*rpl*O_gene_complete_coding_sequence

ATGCGTTTAAATACTCTGTCTCCGGCTGAAGGTGCCAAGCACGCGCCTAAACGCGTAGGTCGTGGTATCGGTTCTGGTCTGGGTAAAACTGGCGGACGTGGTCACAAAGGTCAGAAGTCTCGTTCTGGCGGTGGCGTACGTCGTGGTTTCGAAGGTGGCCAGATGCCTTTATACCGTCGTTTGCCGAAATTCGGCTTTACTTCACGCAAAGCAATGATCACTGCAGAGATTCGTCTGTCTGATTTTGCAGCTGTTGAAGGCGATGTTATTGATCTGAACGCACTGAAAGCCGCGAACGTTGTTGGCATCCAGATTGAATTTGCGAAAGTTATTCTGTCTGGCGAAGTGAACCGTGCAGTTACTGTACGTGGCCTTCGTGTTACTAAAGGTGCTCGCGCTGCAATCGAAGCTGCCGGCGGTAAAATTGAGGAATAA

>*Providencia_vermicola*_DSM_17385_*rpl*P_gene_complete_coding_sequence

ATGTTACAACCAAAGCGTACAAAATTCCGTAAGGTGCACAAGGGCCGCAACCGTGGTCTAGCGCAAGGTACGGATGTTAGCTTCGGCACTTTCGGTCTTAAAGCTGTTGGCCGTGGTCGTCTGACTGCACGTCAGATCGAAGCGGCACGTCGTGCTATGACCCGTGCTATTAAGCGTCAAGGTAAAATCTGGATCCGTGTGTTCCCAGACAAACCAATCACTGAGAAACCACTCGAAGTTCGTATGGGTAAAGGTAAAGGTAACGTAGAATATTGGGTTGCCTTAATCCAGCCTGGAAAAGTCCTGTACGAAATGGACGGTGTGCCTGAAGAGCTGGCTCGTGAGGCATTCTCTCTGGCAGCAGCGAAACTGCCTATCAAAACCACCTTTGTAACTAAGACGGTGATGTAA

>*Providencia_vermicola*_DSM_17385_*rpl*Q_gene_complete_coding_sequence

ATGCGCCATCGTAAGAGTGGTCGTCAATTGAACCGCAACAGCAGCCACCGCCAAGCTATGTTCCGTAACATGGCTGGTTCTTTAGTTCGTCATGAGATCATCAAGACGACTCTGCCTAAAGCGAAAGAACTGCGTCGCGTCGTTGAGCCGCTGATTACTCTTGCCAAGACCGACAGCGTAGCTAATCGTCGTCTGGCATTCGCACGTACTCGTGATAACGAGATCGTTGCAAAATTATTTAATGAACTGGGACCTCGTTTCGCAGCTCGTGCTGGTGGTTACACTCGTATTCTTAAGTGTGGCTTCCGTACAGGCGACAACGCTCCGATGGCTTACATCGAGCTTGTTGACCGTGCTGTTGAGTCTCAAGAAGCAGCTGCAGAGTAA

>*Providencia_vermicola*_DSM_17385_*rpl*R_gene_complete_coding_sequence

ATGGATAAGAAAGCAGCTCGTATCCGTCGTGCGACCCGCGCACGCCGTAAGATCCAAGAATTGGGTGCGACTCGCCTGGTGGTACATCGTACTCCACGCCATATTTATGCGCAGGTTATCGCACCAAACGGTTCTGAAACATTGGTTGCAGCTTCTACTACAGAAAAAGCTATCAATGAACAAGTTAAGTTTACTGGAAACAAAGACGCAGCAGCAGCAGTTGGTAAATTAGTTGCTGAACGCGCACTGGAAAAAGGCATCACTGTTGTTGCTTTTGACCGTTCTGGTTTCCAATATCATGGTAGAGTCCAGGCACTGGCAGATGCTGCCCGTGAAGCTGGCCTTCAGTTCTAA

>*Providencia_vermicola*_DSM_17385_*rpl*S_gene_complete_coding_sequence

ATGAGCAACATTATTAAACAAATTGAACAAGAGCAAATGAAGCAGGACGTACCTTCATTCCGTCAGGGTGACACCGTGGAAGTTAAGGTATGGGTCGTAGAAGGCTCTAAAAAACGTCTGCAGGCATTCGAGGGCGTGGTTATCGCTATTCGTAACCGCGGTCTGCACTCTGCATTCACTGTTCGTAAAATTTCTAACGGCGAAGGTGTTGAGCGTGTCTTCCAAACTCACTCACCAGTCGTAGATAGCATTGCTGTTAAACGCCGTGGTGCTGTTCGCAGAGCTAAACTGTACTACCTGCGTGAGCGTTCTGGTAAGGCAGCTCGTATCAAAGAGCGTCTGAACGCCAAGTAA

>*Providencia_vermicola*_DSM_17385_*rpl*T_gene_complete_coding_sequence

ATGGCTCGCGCAAAACGTGGTGTAATCGCACGTGCACGTCACAAAAAAATTCTGAAACAGGCGAAAGGTTACTATGGTGCACGTTCACGTGTTTACCGCGTAGCGTTTCAAGCGGTAATCAAAGCAGGTCAATATGCTTACCGTGACCGCCGTCAACGTAAACGTCAGTTCCGTCAACTGTGGATCGCGCGTATCAACGCAGCGGCTCGTCAGAACGGTCTGTCTTACAGCCGTTTCATCAATGGCTTGAAAAAAGCTTCAATTGAAATCGACCGTAAGATCTTAGCAGACATCGCAGTTTTCGATAAAGCAGCATTCACTGCTTTAGTTGAAAAAGCGAAAGGCGCTTTAGCTTAA

>*Providencia_vermicola*_DSM_17385_*rpl*U_gene_complete_coding_sequence

ATGTACGCGGTTTTCCAAAGTGGTGGTAAACAACACCGAGTTAGCGAAGGTCAAACTGTCCGCCTAGAAAAGCTGGACATCGCAACAGGTGAAACTGTTGAATTTGATCAAGTTTTAATGGTTGCTAATGGCGATGAGATCCAAATCGGCGCTCCTGTCGTTGAAGGCGTTAAAGTGAAAGCGGAAGTGGTTGCACACGGTCGTGGCGAGAAAGTAAAAATCGTCAAATTCCGTCGTCGTAAACATAGCCGTAAACAACAGGGTCATCGTCAGTGGTTCACTGATGTTAAGATCACTGTCATCGCTTAA

>*Providencia_vermicola*_DSM_17385_*rpl*V_gene_complete_coding_sequence

ATGGAAACTTTAGCTATACATCGCCACGCTCGTTCTTCTGCCCAGAAGGTTCGCTTAGTCGCCGACCTGATTCGCGGTAAGAAAGTGTCGCAAGCTCTGGAAATTCTGACCTATACCAACAAGAAAGCTGCTGGTTTAGTGAAGAAAGTACTTGAATCTGCAATTGCTAACGCAGAGCAAAACGATGGCGCTGACATTGATGACCTGAAAGTTGCGAAAATTTTCGTAGACGATGGTCCAACTATGAAACGCATCATGCCGCGTGCGAAAGGCCGTGCAGATCGTATTCTTAAGCGCACCAGCCACATTACTGTGGTTGTGTCCGATCGCTGA

>*Providencia_vermicola*_DSM_17385_*rpl*W_gene_complete_coding_sequence

ATGATCCGTGAAGAACGTCTGCTGAAAGTACTGCGCGCGCCGCATGTATCTGAAAAAGCTTCTACAGCGATGGAAAAAAGCAATACCATCGTTCTCAAAGTTGCAAAAGATGCAACTAAAGCAGAAATCAAAGCTGCTGTACAGAAACTGTTTGAAGTTGAAGTTGAAGGTGTTAACACTTTGCTGGTTAAAGGCAAAACTAAACGCCACGGTCAGCGTACTGGTCGTCGTAGCGACTGGAAAAAAGCTTACGTTACGCTGAAAGAAGGCCAGAATCTGGACTTCATTAGCGGCGCAGAGTAA

>*Providencia_vermicola*_DSM_17385_*rpl*X_gene_complete_coding_sequence

ATGGCAGCGAAAATCCGTCGTGATGACGAAGTTATCGTGCTAACTGGTAAAGATAAAGGTAAGCGCGGTAAAGTAAAACAGGTTCTTTCTTCTGGTAAAGTTATCGTTGAAGGTATCAATCTGGTTAAAAAACATCAGAAGCCAGTTCCGGCTCTGAATCAACCAGGTGGCATCGTTGAAAAAGAAACGGCTATTCAAGTTTCTAACGTTGCAATCTTTAACGCGGCAACTGGTAAGGCAGACCGTGTAGGCTTTAGATTCGAAGACGGCAAAAAAGTCCGTTTCTTCAAATCTAACAGTGAAACTATCAAGTAA

>*Providencia_vermicola*_DSM_17385_*rpm*A_gene_complete_coding_sequence

ATGGCACACAAAAAGGCTGGTGGTTCGACTCGTAACGGTCGTGACTCAGAAGCAAAACGTTTAGGTGTTAAGCGTTTTGGTGGTGAAGCTGTATTAGCAGGTAGCATCATCGTTCGTCAACGTGGTACTAAGTTCCACGCAGGTAACAACGTAGGTTGTGGCCGTGACCACACTCTGTTCGCATTAGCGGATGGTAAAGTGAAATTTGAAGTTAAAGGTCCTAACAATCGTAAATTTATCAGCATCGAAGCTGAATAA

>*Providencia_vermicola*_DSM_17385_*rpm*B_gene_complete_coding_sequence

ATGTCACGAGTCTGCCAAGTTACTGGCAAGCGTCCAATGAGCGGTAACAACCGTTCACACGCATTAAACGCGACCAAACGTCGTTTTTTGCCAAACCTGCACTCTCACCGTTTCTGGGTTGAGTCTGAGAAACGTTTCGTAACACTGCGTGTATCTGCTAAAGGTATGCGTGTTATCGATAAAAAGGGTATTGATGCTGTTCTTGCTGAACTGCGTACCCGTGGTGAGAAGTACTAA

>*Providencia_vermicola*_DSM_17385_*rpm*C_gene_complete_coding_sequence

ATGAAAGCACAAGAGCTGCGCGTAAAAAGCGTTGAAGAGCTGAATGCTGAACTGCTGAATCTGCTGCGTGAACAATTTAATTTACGTATGCAAGCTGCAAGCGGTCAGCTGCAACAGTCTCATCTGTTGAAACAAGTGCGTCGCGATATCGCACGTGTGAAGACTTTACTGACTGAGAAGGCAGGTGCGTAA

>*Providencia_vermicola*_DSM_17385_*rpm*D_gene_complete_coding_sequence

ATGGCTAAGACTATTAAAATTACACAAGTTCGCAGTTCAATCGGTCGTCTGCCTAAACATAAGGCAACACTGGTCGGTTTAGGTCTGCGTCGCATTGGTCATACAGTAGAGCGTGAGGATACTCCTGCCGTTCGTGGTATGGTCAACTTGGTTTCCTACATGGTTAAAGTTGAGGAGTAAG

>*Providencia_vermicola*_DSM_17385_*rpm*E_gene_complete_coding_sequence

ATGATCCGCCGCCTTTGGTACTTAATTTTACCAATACGTAAATTCAATCAACACGACGTGTGGTGTCCGGCGTTAGGGCTGGAATGGCGACACGGCCTTAATAGAGGTTCTCCCATGAAAAAAGGTATTCACCCTAAATACGAAGAAGTTACTGCAACTTGTTCTTGCGGTAATGTAATGAAAATTAATTCAACTGCTGGTCATTCGCTGAACCTGGACGTTTGTGGTAACTGCCACCCGTTCTATACTGGTAAACAGCGTGATGTTGCGACTGGTGGTCGTGTTGATCGCTTCAACCAACGTTTCAGCATCCCAGGTTCTAAAAAATAA

>*Providencia_vermicola*_DSM_17385_*rpm*F_gene_complete_coding_sequence

ATGGCCGTACAACAGAATAAACCAACTCGTTCAAAACGCGGTATGCGTCGTTCACATGATGCACTGACTGCTACCCTAGTTTCTGTAGATAAAACTTCAGGTGAAACTCACCTGCGTCACCATGTGACTGCAGACGGTTTCTACCGTGGCCGTAAGGTTATCAGCAAGTAA

>*Providencia_vermicola*_DSM_17385_*rpm*G_gene_complete_coding_sequence

ATGGCTAAAGGTATTCGCGAGAAAATCAAGCTGGTTTCTTCTGAAGGTACAGGTCACTTCTATACCACTACGAAGAACAAGCGCACAATGCCAGAAAAACTGGAACTGAAAAAATTTGATCCAGTCGTTCGTAAGCATGTGATCTACAAAGAAGCAAAAATCAAATAA

>*Providencia_vermicola*_DSM_17385_*rpm*H_gene_complete_coding_sequence

ATGAAACGCACTTTTCAACCGTCCGTACTGAAGCGCAACCGTTCACACGGTTTCCGTGCTCGTATGGCCACTAAAAATGGTCGTCAGGTTCTGGCTCGCCGTCGTGCTAAAGGCCGCGCTCGTCTGACCGTTTCATCTAAGTAA

>*Providencia_vermicola*_DSM_17385_*rpm*I_gene_complete_coding_sequence

ATGCCAAAGATTAAAACTGTACGTGGCGCAGCAAAGCGCTTTAAAAAAACTGCAGGCGGTGGCTTTAAGCGTAAGCATGCAAACCTTCGTCATATCCTGACTAAAAAGTCAACTAAGCGTAAACGCCATTTACGTCCGAAAGGAATGGTATCTAAAGGCGATCTGGGTCTGGTAGTTGCTTGCCTGCCGTACGCATAA

>*Providencia_vermicola*_DSM_17385_*rpm*J2_gene_complete_coding_sequence

ATGAAAGTTCGTGCTTCCGTCAAGAAATTATGCCGTAACTGTAAAGTTATCCGTCGCCATGGTAGCGTTCGTGTAATTTGCAGTGTTGAACCAAGACATAAACAACGTCAAGGTTAA

>*Providencia_vermicola*_DSM_17385_*rps*A_gene_complete_coding_sequence

ATGACTGAATCTTTTGCTCAACTCTTTGAAGAATCCCTGCAGAATATTGAAACTCGTCCTGGTTCTATCGTCCGTGGTACTGTTGTTGCCATCGACAAAGACGTAGTCCTGGTTGACGCAGGTCTTAAATCAGAATCTGCTATCCCAGTAGAACAGTTCAAAAATGCTCAGGGTGAGCTAGAAATCCAAGTTGGCGACGAAATCGATGTGGCTCTGGATGCAGTAGAAGATGGTTTCGGTGAAACTATCCTGTCTCGTGAGAAAGCGAAACGTCATGAAGCATGGCTGATGCTGGAAAAAGCTTACGAAGAAGCTGAAACTGTAACTGGTGTTATCAACGGTAAAGTTAAAGGTGGTTTCACTGTTGAACTGAACGGCATTCGTGCGTTTTTACCAGGTTCACTGGTAGATGTACGCCCAGTTCGTGATACTACTCACTTGGAAGGCAAAGAGCTTGAGTTCAAAGTAATCAAGCTAGATCAGAAACGCAACAACGTTGTTGTTTCTCGTCGTGCTGTAATTGAATCTGAAAGCAGCGCTGAGCGCGATCAACTGCTGGAAAATCTGCAAGAAGGCATGGAAGTTAAAGGTATCGTTAAGAACCTTACTGACTACGGTGCATTCGTTGATCTGGGCGGTGTTGACGGCCTGCTGCACATCACTGACATGGCTTGGAAACGTGTTAAACACCCAAGCGAAATCGTCAACGTTGGTGATGAAATCAATGTTAAAGTTCTGAAATTCGACCGTGAGCGCACTCGTGTTTCTCTGGGTCTGAAACAACTGGGCGAAGATCCTTGGGTCGCAATCGCTAAACGTTACCCAGAAGGTACTAAACTGACTGGCCGCGTTACTAACCTGACTGACTATGGTTGCTTCGTTGAAATCGAAGAAGGCGTTGAAGGTCTGGTTCACGTTTCAGAAATGGATTGGACTAACAAAAACATCCACCCATCTAAAGTTGTTAACGTTGGTGATGTTGTTGAAGTTATGGTTCTGGATATCGATGAAGAACGTCGTCGTATCTCACTGGGCCTGAAACAATGCAAATCTAACCCATGGCAGCAATTTGCAGAAACTCACAACAAAGGCGACCGCGTTGAAGGTAAAATCAAGTCTATTACTGACTTCGGTATCTTCATCGGACTGGACGGCGGCATCGATGGCCTGGTTCACCTGTCTGACATCTCCTGGAACGTTGCAGGCGAAGAAGCAGTTCGTGAATACAAAAAAGGTGACGAAATCGCAGCTGTTGTTCTGCAAGTCGACGCAGAGCGTGAGCGTATTTCTTTAGGTGTTAAACAGTTAGCTGAAGATCCATTCAATAACTACTTAGCAGCAACTAAGAAAGGCGCAATCGTAACTGGTAAAGTTATTGCGGTTGATGCTAAAGGTGCAACTGTTGAGCTGACTCTGGGCGTTGAAGGTTACCTGCGTGCATCAGAAGCTTCACGTGACCGTGTTGAAGATGCAACACTGGTTCTGAACGTTGGTGATGATGTTGAAGCTAAATACACTGGTGTTGATCGTAAAAACCGTGTAATCAACCTGTCTGTTCGCGCTAAAGACGAAGCTGATGAGAAAGACGCTGTTGCAGCTGTGAACAAGCAAGAAGATACAGCATTCGGCAACAACGCTATGGCTGAAGCTTTCAAAGCAGCTAAAGGCGAATAA

>*Providencia_vermicola*_DSM_17385_*rps*B_gene_complete_coding_sequence

ATGGCAACTGTTTCCATGCGCGATATGTTACAAGCGGGCGTTCACTTTGGTCACCAGACTCGTTACTGGAACCCAAAAATGAAACCTTTCATTTTCGGCGCGCGTAATAAAGTTCATATCATCAACCTGGAAAAAACTGTTCCAATGTTCAACGAAGCTCTGGCTGAGTTGACTAAAATTGCTTCTCGTAAAGGCAAAATTCTGTTTGTTGGTACTAAACGTGCTGCAAGCGAAGCCGTTCAAGAAGCTGCTAACAGCTGTGACCAATATTTCGTTAACCACCGTTGGTTAGGCGGAATGTTGACTAACTGGAAAACTGTTCGTCAGTCAATCAAACGTCTGAAAGATTTAGAAGTTCAATCACAAGACGGTACTTTCGACAAACTGACCAAGAAAGAAGCGCTGATGCGTACTCGTGAACTTGGTAAGTTAGAAAACAGCTTAGGCGGTATCAAAGATATGGGCGGTTTACCTGACGCTCTGTTCGTTATCGATGCAGACCACGAACACATTGCTATCAAAGAAGCAAACAACCTGGGTATCCCAGTATTTGCTATCGTTGATACTAACTCTGATCCAGATGGTATTGATTTCATTATCCCTGGTAACGACGATGCAATCCGTGCAATCAAACTGTATCTGGGCGCTGTAGCAAGCACCGTTCGTGAAGGTCGTTCGCAAGATCTGGCTGTTCAAGCAGAAGAAAGCTTAGTAGAAGCTGAATAA

>*Providencia_vermicola*_DSM_17385_*rps*C_gene_complete_coding_sequence

ATGGGTCAGAAAGTACATCCTAATGGTATTCGCCTGGGTATTGTCAAACCTTGGAACTCTACTTGGTATGCGAATACTAATGAATTCGCTGACAACCTAGACAGCGATTTTAAAGTACGTCAGTACTTGAATAAAGAACTGGCAAAAGCGTCAATCTCTCGTATCGTTATCGAACGTCCTGCGAAAAGCATCCGTGTGACTATTCACACTGCTCGCCCAGGTATCGTTATCGGTAAGAAAGGTGAAGACGTTGAAAAACTGCGTAAAACAGTAGCGGATATCGCTGGTGTTCCTGCGCAAATCAATATCGCCGAAGTTCGTAAACCAGAACTAGACGCAAAATTAGTTGCTGACAGCATCTCTTCACAGCTGGAACGTCGTGTTATGTTCCGTCGTGCTATGAAGCGTGCAGTACAGAACGCAATGCGTCTGGGCGCTAAAGGTATTAAAGTTGAAGTCAGTGGCCGTTTAGGCGGTGCTGAAATCGCTCGTACCGAGTGGTATCGTGAAGGCCGTGTGCCACTGCACACATTACGTGCTGATATCGATTACAACACTTCAGAAGCGCACACCACATATGGTGTTCTCGGCGTTAAGGTATGGATCTTCAAAGGTGAGATTCTGGGTGGTATGGCTGCTGTTGAACAAGCTGAGAAACCGGCTGCTCAACCTAAAAAGCAGCAGCGTAAAGGCCGCAAGTAA

>*Providencia_vermicola*_DSM_17385_*rps*D_gene_complete_coding_sequence

ATGGCTAGATATTTGGGTCCTAAGCTCAAGCTGAGCCGTCGCGAAGGAACAGACCTCTTTCTGAAGTCTGGTGTTCGCGCGATTGACACCAAGTGTAAATTAGAACAGGCACCAGGCCAGCACGGAGCGCGTAAACCGCGTCTGTCTGACTACGGTGTTCAGTTACGTGAAAAACAAAAAGTTCGTCGTATCTACGGTGTTCTGGAACGTCAATTCCGTAACTATTACAAAGAAGCAACACGTCTAAAAGGCAACACAGGTGAAAACCTGCTGACTCTGCTGGAAGGTCGTCTTGATAACGTCGTTTATCGTATGGGCTTTGGCGCAACTCGCGCAGAAGCACGTCAAATGGTTAGCCATAAAGCTATCATGGTAAATGGTCGTGTTGTTAATATCGCTTCTTATCAGGTTTCCCCGAATGACGTTATCAGCGTTCGTGAGAAAGCTAAAAAACAGTCTCGTATTAAGGCTGCTTTAGAGCTGGCTGAACAGCGTGAGAAGCCAACTTGGCTGGAAGTTGATGCTGCTAAAATGGAAGGTGTGTTCAAACGTATTCCTGAACGTACTGACTTGTCTGCGGACATTAACGAACACCTGATCGTCGAGCTTTACTCCAAGTAA

>*Providencia_vermicola*_DSM_17385_*rps*E_gene_complete_coding_sequence

ATGTCTCACATCGAAAAACAAGCTGGCGAACTGCAGGAAAAGCTGATCGCGGTAAACCGCGTAGCAAAAACCGTTAAAGGTGGCCGTATTTTCAGCTTTACCGCACTGACTGTAGTTGGTGATGGTAACGGTCGCGTTGGTTTTGGCTACGGCAAAGCGCGCGAAGTTCCGGCAGCAATCCAGAAAGCGATGGAAAAAGCCCGTCGCAGTATGAAAACCGTTGCTCTTAACAACGGCACATTATTCCACCCAGTGAAAGGTACACACACCGGTTCTCGCGTGTTTATGCAGCCTGCTCACGAAGGTACTGGTATTATTGCCGGTGGTGCAATGCGTGCAGTGTTAGAAGTAGCTGGAGTTCGCAACGTACTGGCTAAAACCTACGGTTCCACAAACCCAATCAACGTGGTTCGTGCAACACTGGATGCTTTAGACAGCATGAAGTCTCCAGAAATGGTCGCAGCTAAGCGTGGAAAATCCGTCGAAGAAATTCTGGGGTAA

>*Providencia_vermicola*_DSM_17385_*rps*F_gene_complete_coding_sequence

ATGCGTCATTACGAAATCGTTTTTATGGTCCATCCTGACCAAAGCGAACAGGTTCCGGGCATGATCGAGCGTTACAGTGCAGTAATCACTAACGCACAAGGTCAGATTCACCGTCTGGAAGACTGGGGCCGCCGTCAACTGGCTTACCCAATCAACAAACTGCACAAAGCTCACTATGTTCTGCTGAACGTAGAAGCACCGCAGGAAGCGATTGATGAGCTGGAAACTAACTTCCGCTTCAACGATGCCGTTATCCGCAGCATGGTTATGCGCTTAAAACACGCAGTAACAGAAGCTTCTCCAATGGTTAAAGCTAAAGACGAACGTCGTGGCCGTGACCTTTCTGATGAATATCAGGATGAAGAAGTAGAAGAAACTGGGGATTCTGAAGAGTAA

>*Providencia_vermicola*_DSM_17385_*rps*G_gene_complete_coding_sequence

ATGCCACGTCGTCGCGTAATAGGTCAACGTAAAATTCTTCCAGATCCTAAGTTCGGATCAGAATTACTGGCCAAGTTTGTAAATATCCTGATGGTAGACGGGAAAAAATCTACTGCTGAAGCAATCGTATATAACGCACTTGAAACCCTTGCTCAGCGTTCTGGTAAAACTGAACTTGATGCATTCGAATTAGCACTGGATAACGTGCGTCCGACTGTGGAAGTTAAATCCCGCCGTGTTGGTGGTTCAACTTACCAAGTTCCAGTTGAAGTTCGCCCGGTTCGTCGTAATGCCCTGGCAATGCGTTGGATCGTTGATGCTGCTCGTAAACGCGGTGATAAATCTATGGCACTTCGCCTGGCAAATGAATTATCAGACGCTGCTGAGAACAAAGGTTCCGCTGTTAAGAAACGTGAAGACGTTCACCGTATGGCAGAAGCTAACAAGGCGTTCGCACACTACCGTTGGTAA

>*Providencia_vermicola*_DSM_17385_*rps*H_gene_complete_coding_sequence

ATGAGCATGCAAGATCCCATCGCGGATATGCTGACCCGTATCCGTAACGGTCAGGCCGCGAACAAAGTTGCGGTCACCATGCCTTCCTCCAAGCTGAAAGTGGCGATTGCCAAAGTGCTGAAGGAAGAAGGTTATATTGAAGATTTTAAAATTGAAGGCGACATCAAGCCAGAACTGGAACTGACTTTACGTTATTTCCAAGGTAAGGCTGTTGTAGAAAGCATTCAGCGTGTAAGCCGCCCAAGTCTGCGCATCTATAAGAAAAAAGATGAGCTGCCACAAGTTATGGCTGGCCTAGGTATCGCTGTTGTTTCTACCTCTAAAGGTGTCATGACTGATCGTGCAGCTCGCCAAGCTGGTCTTGGTGGCGAGATTCTCTGCTACGTAGCTTAA

>*Providencia_vermicola*_DSM_17385_*rps*I_gene_complete_coding_sequence

ATGGCTGAAAATCAATACTACGGCACTGGTCGCCGCAAAAGCTCATCTGCTCGTGTCTTTATTAAGCCGGGTAGCGGTAACATCACAATCAATCAGCGTACGCTGGAACAGTACTTTGGTCGCGAAACTGCGCGCATGGTCGTTCGTCAGCCGTTAGAATTGGTTGAAATGTTGGAAAAACTGGATCTGTACATCACTGTTAAAGGTGGTGGTATTTCAGGTCAAGCAGGCGCAATCCGTCACGGTATTACTCGTGCACTGATGGCTTATGATGAGACTCTTCGTTCTGATCTGCGTAAAGCTGGTTTCGTTACCCGTGATGCGCGTTCTGTTGAACGTAAGAAAGTGGGTCTGCGTAAAGCTCGTCGTCGTCCACAGTTCTCCAAACGTTAA

>*Providencia_vermicola*_DSM_17385_*rps*J_gene_complete_coding_sequence

ATGCAGAACCAAAGAATCCGTATCCGCCTTAAAGCTTTTGATCATCGTTTAATCGATCAATCAACTGCGGAAATCGTAGAGACTGCTAAGCGCACTGGTGCGCAGGTACGTGGTCCTATCCCGCTGCCGACACGTAAAGAGCGTTTTACCGTTCTGATTTCTCCGCACGTTAATAAAGATGCGCGTGATCAGTACGAAATTCGCACTCACAAACGTCTGGTTGACATCGTTGAGCCAACCGAGAAGACCGTTGATGCTCTGATGCGTCTGGACCTGGCTGCCGGCGTTGACGTGCAGATCAGCCTGGGTTAA

>*Providencia_vermicola*_DSM_17385_*rps*K_gene_complete_coding_sequence

ATGGCAAAGGCACCAGTTCGTGCACGTAAGCGTGTAAGAAAACAAGTCTCAGACGGTGTGGCTCATATCCATGCTTCTTTCAACAACACAATCGTTACAATTACTGACCGTCAGGGTAACGCATTAGGTTGGGCAACTGCCGGTGGTTCCGGTTTCCGTGGTTCTCGCAAATCCACTCCGTTCGCAGCTCAGGTTGCGGCAGAGCGTTGCGCAGAAGCTGTGAAAGAATACGGAATCAAAAACCTGGAAGTTATGGTTAAGGGACCGGGTCCGGGTCGCGAATCAACAATTCGTGCTCTGAACGCCGCTGGTTTCCGCATCACTAATATTACTGATGTGACTCCTATCCCTCATAACGGTTGTCGCCCACCGAAAAAACGTCGCGTTTAA

>*Providencia_vermicola*_DSM_17385_*rps*L_gene_complete_coding_sequence

ATGGCAACTATTAATCAGCTGGTACGCAAATCTCGTAGCTCGAAAGTTGTGAAAAGCAACGTTCCAGCACTGGAAGCTTGCCCGCAAAAACGTGGCGTATGTACTCGTGTATATACTACCACTCCTAAAAAACCAAACTCAGCATTACGTAAAGTATGCCGTGTTCGTTTAACTAATGGTTTCGAAGTATCTTCCTACATCGGTGGTGAAGGCCACAACTTGCAGGAACACTCCGTAATCCTTATCCGTGGCGGTCGTGTTAAAGACTTGCCAGGTGTGCGTTATCACACCGTTCGCGGCGCACTGGACTGTTCTGGTGTTAAAGACCGTAAGCAAGCTCGTTCTAAATACGGCGCGAAAAAACCTAAGGCTTAA

>*Providencia_vermicola*_DSM_17385_*rps*M_gene_complete_coding_sequence

GTGGCCCGTATAGCAGGCATTAACATTCCTGATCATAAACATACCGTAATCGCTTTAACATCGATTTTCGGAATCGGCAAAACTCGTTCACAGGCTATCTGTGAAGCAACTGGTATTGCTGAAAATGTTAAGATCAGTGAGCTGTCTGAAGAACAAATCGACAAGCTGCGTGACGAAGTTGCCAAGTATGTGGTAGAAGGTGACCTACGTCGTGAAATTACCCTGAGCATCAAGCGTCTGATGGACCTTGGATGTTACCGTGGTTTACGTCATCGTCGTGGTCTTCCTGTGCGCGGACAGCGTACTAAGACTAACGCTCGTACCCGCAAGGGTCCGCGTAAGCCGATCAAGAAATAA

>*Providencia_vermicola*_DSM_17385_*rps*N_gene_complete_coding_sequence

ATGGCTAAGAAATCTATGAAAGCGCGTGATGTTAAACGTGCTAAATTAGCTGAGAAGTTCTTCGCAAAACGCGTTGAATTGAAAGCTATCATCTCTGATGTTAAAGCATCTGATGAAGATCGCTGGGACGCTGTTCTCAAGCTGCAAACACTGCCACGTGATTCAAGTCCTTCTCGTCAGCGTAACCGCTGCCGTCAAACAGGGCGTCCGCACGGTTTCCTGCGGAAGTTTGGTCTGAGCCGTATTAAAGTCCGTGAAGCCGCAATGCGCGGTGAAATCCCGGGCCTTAAAAAGGCAAGTTGGTAA

>*Providencia_vermicola*_DSM_17385_*rps*O_gene_complete_coding_sequence

ATGTCTCTAAGTAATGAAGCGAAAGCTAAGATCGTTGCTGAGTTCGGTCGCGGTGAAAATGACACTGGTTCAAGCGAAGTTCAAATCGCTCTTCTGACTGCACAAATCAACCATCTGCAAAGCCACTTTTCAGAGCACAAAAAAGATCACCACAGCCGTCGTGGTCTGCTGCGTATGGTTGCACAACGCCGTCGCTTACAAGCTTACTTGAAAGGTAAAGATATCGCACGCTACACAGCTCTGATCGAGCGTTTAGGCCTGCGTCGCTAG

>*Providencia_vermicola*_DSM_17385_*rps*P_gene_complete_coding_sequence

ATGGTAACAATTCGTTTATCTCGTGGCGGCGCTAAAAAACGTCCGTTCTACCAAATCGTTGTAACCGATAGCCGCAATGCGCGTGACGGTCGTTTCATTGAGCGTATCGGTTTCTTCAACCCGATCGCTTCAGGTCAAGCAGAAGAACTGCGTTTAGACTTGGACCGTGTTGAGCATTGGGTTGGCTTAGGCGCAACTATTTCTGACCGTGTTGCACAACTGGTCAAACAAGCTAAGAAAGCAGCTTAA

>*Providencia_vermicola*_DSM_17385_*rps*Q_gene_complete_coding_sequence

ATGAGCGATAAAATCCGTACTCTGCAAGGTCGTGTAGTTAGCGATAAAATGGAGAAATCTATTGTTGTTGCTATCGAGCGTATGGTGAAACACCCTCTGTATGGTAAATTCATCCGTCGTACGACTAAATTGCACGTACATGACGAGAACAATGAGTGTGGAATTGGTGACGTGGTAGAAATCCGCGAAACTCGTCCACTGTCTAAGACTAAGTCTTGGGCCTTAGTTCGCGTTGTAGAAAAAGCTGTTCTGTAA

>*Providencia_vermicola*_DSM_17385_*rps*R_gene_complete_coding_sequence

ATGGCACGTTATTTCCGTCGTCGCAAGTTCTGCCGTTTCACAGCGGAAGGCGTTCAAGAGATCGACTATAAAGATATCGCAACGCTGAAAAACTATATCACTGAAAGTGGTAAAATTGTACCAAGCCGTATCACCGGTACTCGTGCAAAATATCAGCGTCAGCTCGCTCGTGCTATCAAGCGCGCTCGCTACCTGTCTCTGTTACCATATACTGATCGTCATCAGTAA

>*Providencia_vermicola*_DSM_17385_*rps*S_gene_complete_coding_sequence

ATGCCACGTTCTCTCAAGAAAGGTCCTTTCATTGACCTGCACTTGCTGAAGAAGGTAGAGAAAGCGGTGGAAAGCGGAGACAAGAAGCCTCTCAAGACTTGGTCCCGTCGTTCAACGATCTTTCCTAATATGATCGGATTGACCATCGCTGTCCATAATGGTCGTCAGCATGTTCCAGTTTTCGTAACCGACGAAATGGTTGGTCACAAACTGGGTGAATTCGCACCGACTCGCACTTATCGCGGCCATGCGGCAGATAAGAAAGCTAAGAAACGTTAA

>*Providencia_vermicola*_DSM_17385_*rps*T_gene_complete_coding_sequence

TTGGCTAATATCAAATCAGCTAAGAAACGTGCCGTTCAGTCAGAGAAACGTCGCCAGCACAACGCTAGCCGTCGCTCTATGGTACGTACTTTTATCAAGAAAGTTTACCTTGCTATCGCTGCAGGCGATAAAGAAGCTGCTCAGAAAGCATTCAATGACATGCAACCTATTGTTGATCGTCACGCCGCTAAAGGCCTGATCCACAAAAATAAAGCAGCACGTCATAAAGCTAACTTAGTTGCTCAAATTAAAGCAATGTAA

>*Providencia_vermicola*_DSM_17385_*rps*U_gene_complete_coding_sequence

ATGCCGGTAATTAAAGTACGTGAAAACGAGCCATTCGACGTTGCACTGCGTCGTTTCAAACGTTCCTGTGAAAAAGCAGGTGTTTTAGCTGAAGTTCGTCGTCGTGAATTCTATGAAAAACCAACGACTGAACGTAAACGCGCTAAAGCATCAGCTGTTAAGCGTCACGCTAAGAAACTGGCTCGCGAAAACGCACGCCGTACTCGTCTGTACTAA
