# Supplementary material for: Genomic analysis of the nomenclatural type strain of the nematode-associated entomopathogenic bacterium Providencia vermicola
Source: BMC Genomics. 2021 Oct 2;22:708. doi: 10.1186/s12864-021-08027-w (PMC8487129; doi:10.1186/s12864-021-08027-w)
Supplement: Supplementary file 3 — Additional file 3. Sequences of Providencia vermicola DSM_17385 putative antibiotic.resistance-associated proteins [file 12864_2021_8027_MOESM3_ESM.docx]

**Additional File 3.** Sequences of *Providencia vermicola* DSM_17385 putative antibiotic resistance-associated proteins

>*Providencia_vermicola*_DSM_17385_ARO:3004122_OmpK37

MKRNILAMVIPALLAAGAANAAEVYNKDGNKLDVYGKVDVRHYFADGKGSGKTSSEDGDDSRVRLGLKGDTQITDQLTGFGRFEWETKTNKAEDSNENKNRLAYAGLKFADFGSIDYGRNYGVIYDTNAWTDVMPLWGGDTMAQSDNFMTSRNRNLLTYRNNNAFGYVDGLSFALQYQGKNGDDNKSSSGNPRKDNGDGYGFSTAYDLGWGVTLGGGYSNSSRTPDQRNKTTAGGQKAQAWNVGGKFDANNVYLAAMYGQTLNTTRYGSGNLVANKTENVELVAQYLFDFGLKPSLGYNQSKGKDLGGYGNKDLVKYISVGSYYYFNKNMSAVVDYKINLLKDNTFTDQVGINTDNVVGLGLIYQF

>*Providencia_vermicola*_DSM_17385_ARO:3001329_MdtG

VLNMTGNMTGKTEYWKRNLYVVWFGCFLTGAAFSLIMPFLPLYIEELGVKDHASLNLWTGAVFSITFLFSAIAAPFWGRLSDRKGRKLMLLRSALGMAIVMVLIGFAQNIWQLLALRALLGLLGGFVPNANALIATQIPVKKSGWAMGVLATGAVSGALIGPLIGGFLADQYGLRPVFFITATVLFICFFVTLFYVRERFTPVSRKDALTSKQVFASLRNKNLVISLFFTTMIIQAAMGSINPVITLYVRELSDSLENLAFVSGVIASIPGVAALISAPMLGKLSDRIGPEKVLLAVLGTSIFVLFPMGLVSSYWELGFLRFLLGALNAALLPAVQTLIIYNISHQVTGRIFSYNQALRDVGNVTGPLMGSFVAATYGFRAVFFFTAALVLFNLIYSWLIIRRQSDGLRASPTEQ

>*Providencia_vermicola*_DSM_17385_ARO:3000002_VanW

MRRPLSSYHPILYWLRVTQKRLARRLHWAFSSKYYSKAKSSERLEYRYNKHTSKLIRKLGDSDLRLQHNKVINLRIAVQAMNGVTIYPGEYFSFCRLVGKPTARRGFVEGMELSFGEARSGIGGGICQLSNLIHWMILHSPLQVVERANHSFDPFPDEGRVLPFGSGAAIFYNYIDLVFYNPTQSAFQLVFNIAEHQLEGELLCSEPRDVKYHIYQKNHKFTREKNIVYRHNEIWQKVITKGQEPVTLQDNCLYRNKVVVKYAIDDSQIMAS

>*Providencia_vermicola*_DSM_17385_ARO:3005162_FosA7.5

MLNGINHLTLAVRDITKSIYFYQSLLGMKLHAKWKHGAYISCGDLWLCLSVDISRQHLAPNETDYTHYAFSISESNFALLVNKLKENEVVVWKENKSEGDSFYFLDPDGHKLELHVGDLTQRLKSCQCSPYDGMEFY

>*Providencia_vermicola*_DSM_17385_ARO:3002685_CatIII

MSYTRFNVEAWQRKEHYHVYNEQVNCGFSLTVKIDITSLYRFISLRDYRFYPTMIYLLSSIVNKHDEFKLSKKNNELILWDKVHPSFTIFHNQTETFSSLWCEYSDNIHIFMDNYHHQLALYKDDNKLAPQPAQAENIFYISSLPWVSFDSFNLNIANIASNFTPIFTMGKFYREGDKILLPLSIQVHHAVCDGFHVGRFVNELQMLCNQLAD

>*Providencia_vermicola*_DSM_17385_ARO:3001216_MdtH

MAQVSRARSLGKYFLLLDNMLVVLGFFVVFPLISIRFVDQLGWAAVVVGFALGLRQFVQQGLGIFGGAIADRFGAKPMIVIGMLLRASGFALMAIAYDPWVLWLSCVLSALGGTLFDPPRTALVIKLTRPYERGRFYSLLLMQDSAGAVIGALIGSWLLQYDFHYVCWAGAVVFIIAAICNAWLLPAYRISTVRTPIKEGMSRVLKDKRFVTYVVTLAGYFMLSVQVMLMFPIVVNELAGTPTAVKWMYAIEAMISLTLLYPIARWSEKHFRLEQRLMAGLFLMSLSMFPIGMTTSLNMLFGLICLFYLGTVTADPARETLSASLADPRARGSYMGFSRLGLALGGAVGYTGGGWMYDIGHQWNMPQLPWFLLGIIGFITLWALHKQFNRKKIETVMLSGQ

>*Providencia_vermicola*_DSM_17385_ARO:3000164_ErmX

MNNRVHQGHFARKRFGQNFLTDQFIIDSIVDAMNPLPGQSIVEIGPGLGALTEPVGSRIEKMTVVELDRDLAARLHVHPQLKDKLTIIQQDAMTVDFGELAKQAGQPLRVFGNLPYNISTPLMFHLFTFTNSIADMNFMLQKEVVNRLVAGPGSKAFGRLSVMAQYYCHVVPVLEVPPTAFTPAPKVDSAIVRLIPHRENPYPVKDIKFLSRITTQAFNQRRKTIRNSLGDLFSVEELTELGIDLSTRAENISVEQYCKMANYLSNRSE

>*Providencia_vermicola*_DSM_17385_ARO:3000237_TolC

MVRFRQQAQLNLLFNQGIKMKKLLPLLIAMSFVGLSTNSYADDLLQVYQKSKESNPELRKSLAERNQAFEKINEARSPLLPQLGLGAGASYGSGYRDANNTESTGLNASLKLTQTVFDMSKWRQLTIQEKTAGISDVTYQTSQQQLILDTATAYFNVLKAIDALSYIEANKEAVYRQLDQVTQRFNVGLVAITDVQNARANYDSVIAQEVAGRNDLENAIEKLRQVSGVYYNQLASLNIERFKTTELGDINAILKEAEERNLSLLSARLAQDVSRENIRLAETGHMPTVSLDASTAVTNTYNHGSGYNNSLTGKGASNSYNGQNSIGLNLSIPLYTGGATSSQVEQAQYGFQGASEQLESVYRNVVQLVRSSFNNVSSSISSINAYKQVVVSAQSSLDAMEAGYQVGTRTIVDVLTSTTALYQAKQNLSNARYDYMINQLNIEFARGTLNEDDIARLNANLGKEISTEPSSIIRNTNAPQIK

>*Providencia_vermicola*_DSM_17385_ARO:3003776_MurA*

MDKFLVKGPTRLSGEVTISGAKNAALPILFAALLAEEPVEIQNVPHLRDIDTTIKLLNQLGTKVKRNGSVFVDASTVSTYCAPYDLVKTMRASIWALAPLVARFGHGEVSLPGGCAIGARPVDLHISGLEQLGANIVLEDGYVKATVDGRLKGACIVMDKVSVGATVSIMTAATLAEGTTVIENAAREPEIEDTANFLNTLGAKITGAGTDRIVIEGVERLGGGTYQVLPDRIETGTFLIAAAVSRGKVICRNARPDTLDAVLAKLREAGATIEIGDDWISLDMEGKRPKAVTIRTAPHPGFPTDMQAQFSLLNLVAEGAGLITETIFENRFMHIPELIRMGAHAEIESNTVLCHGVEKLTSAQVMATDLRASASLVIAGCIAEGTTTVDRIYHIDRGYEHIEDKLRGLGADIQRVHSDD

>*Providencia_vermicola*_DSM_17385_ARO:3004441_Tet(59)

MNKSAITALTITSLDAIGIGLIMPVLPALLREYVSAENLAHHYGILLALYAIMQVFFAPILGKLSDKLGRRPVLLLSLAGAAVDYTLLALSSTLWMLYLGRLISGITGATGAVAASIIADSTEPHERTKWFGRLGAAFGVGLIAGPMIGGLAGEYSPHLPFIIAAILNTGAFIGVSLIFKDRKIKCKQEDTSKSPVVSISFMQVIKPITLLIFVFFMAQLIGQIPATTWVLFTENRFQWGSMEVGLSLAGLGVMHALFQAFVAGAIAKKFNEKTTILVGFVVDGSAFLILSFISQGWMIYPTLILLAGGSIALPALQGLMSTQVNQANQGKLQGVLVSLTNTTGVIGPLLFSFIFGQTLTVWDGSVWLIGAILYLLLIMVYPYCQFKINKRSNQTKTYSY

>*Providencia_vermicola*_DSM_17385_ARO:3000768_AbeS

MSPKAKSWLWMLAVIISETSATSTLKMFDNSEGSTKSLLLALIVVLYVVCYYSLSRAVKYIPVGLAYATWSGTGILMVSTLGMLFYGQHPDTAAMIGMAVIASGIVIMNLFSKMGADEEDTEEPNKETKESLLSTNNKAIH

>*Providencia_vermicola*_DSM_17385_ARO:3004039_EmrE2

MKGLSFLLMSILAEVIATTTLKASEGFSRFWPSLIVIIGYGVSFWGLSQVVKVMPLGIAYAIWSGLGIVLVSVAAIFIYHQKLDLPAIIGMLLIIIGVLVINLFSKSSTH

>*Providencia_vermicola*_DSM_17385_ARO:3000792_MdtA

MNKQNRRKSFTRSAIFVAIIVAAGATWYFYNQEKTADTPNSSAIAKPNSQRSAMGRPPRVLAPVQFAVAEQKVVPRFLSGLGTVQAANVVTVTSRVEGQLMKLYFTEGQYVNAGDLLAQIDPRPFEVQLAQAEGQLAKDKATLANAQLDLARYQKLAGTKVISQQELDNQRALVLQAQGSIKVDQAAVDSAKLQLTYSKITAPISGRVGLKQVDVGNFVSSGTSTPIVVITQTQPADVLFALPEGDIPAIQQAQASSKNVLIEAWDRNNIALIARGELLSTDNQIDAATGTLKIKARFTNEEQKLFPNQFVNVKMQVETLQDAVVIPTAALQMGNEGHYVWILSEDNKVSKHTVTVGMQDSQQVVIESGLAANTKVITDGVDKLTDGTTVEVVTPESLAKKDKEPRKSKPSSAEKA

>*Providencia_vermicola*_DSM_17385_ ARO:3000793_MdtB

MLKGGGPSRLFILRPVATTLFMVAILLAGIIGYRFLPVSALPEVDYPTIQVVTLYPGASPDVMTSAVTAPLETQFGQMSGLKQMSSQSSGGASVITLMFQLSLPLEVAEQEVQAAINSASSLLPSDLPYPPIYNKVNPADPPVLTLAVTSDAMPLTQVQDIIETRIAQKISQVNGVGLVTLAGGQRPAVRVNLNPQAMAAKGLDSETIRVAINNANVNSAKGSFDGPTRAVTLSANDQMKSLDDYRKLIVAFQNGAPVRLGDIATIKEGAENAYLGAWANNEQAIVINVQRQPGANVIETTDTIRTLLPELIESLPKSVNVDILTDRTSTIRASVNDVQFELMLAIALVVMVIYLFLRNGIATLIPGIAVPLSLVGTFAVMYFCGFSVNNLTLMALTIATGFVVDDAIVVIENISRYLEQGDKPLVAALKGAGEIGFTIISLTFSLVAVLIPLLFMGDIVGRLFREFAITLAVAILISAVVSLTLTPMMCARLLKPESQHKHNRFELACERFFDRMVAGYAIWLKRILNHQWLTLSVALGTMVLTVLLYMWIPKGFFPLQDSGIIQGSIESRQSISFSAMAQKQQEVADKLLADPAVDNVTTYVGVDGTNATLNNARLQITLKPLDEREDRVVAIIARLQQSADNIAGVNLYLQPMQDLTIDTQVARTQYQFTLQASTLDELSTWVPKLMSELEQQPELTDVSSDWQNKGLVAYVNVNRDTASRFGITMSSIDNALYNAFGQRMISTIYTQSNQYRVILEHDTQTRDGMDALNDIRLKGTDGAIVPLSTLVNIQEGYGPLAINHLDQFPAVTFSFNVADGASLESAVNAVKHAESQISMPKSITTQFQGATLAFEAALSSTIWLIAAAIVAMYIVLGILYESFIHPITILSTLPTAGVGALLALMMAGKDLDVIAVIGIILLIGIVKKNAIMMIDFALAAEREQGMSPYDAIYQACLLRFRPILMTTMAALLGALPLMLSTGVGAELRQPLGICMVGGLIMSQILTLFTTPVIYLLFDRLSHYLKARRQINKAQVS

>*Providencia_vermicola*_DSM_17385_ARO:3000794_MdtC

MKRFFALFISRPVATTLISVAITLCGALSFLFLPVAPLPQVDYPVINVTASLPGASPETMASSVATPLERSLGSIAGISEMTSSSALGRTTITLEFNLDKDINNAAREVQAAINAAQTLLPSGMPSRPRYYKSNPSDAPIMILTMTSQTLGTGEIYDIASTRLAQRIAQIEGVSEVSVGGGSLPAVRVELNPTALFNQGVSLDAVRSAISSANVRQPQGYINDDEKRYQVQTNDELKKAADYRPIIVHYKAGNAVKLSDVANVKDSIENVRAAGMADGKPAILIVIRREAGANIIETVNRIRAEIPEFRDMIPAAIDLKVAQDRTPTIRASLAEVERALLIAVALVILVVLLFLRSGRATLIPAIAVPVSLIGTFTAMYLCGFSLNNLSLMALTVATGFVVDDAIVVLENISRHIENGMKPFMAAVKGVHEVGFTVVSMSISLVAVFIPLLLMDGLVGRLFREFAITLATAIGISLIVSLTLTPMMCAYLLKKRGKNSKAKHRGFGRILLWVQERYGIALNWVLNHRRSVLAILFATIGLNVYLYITVPKAFFPEQDTGRVLGFVRADQSISFQSMKEKMTRFMEQVKEDPAIDNVTGFTGGSRVNSGFMYISLKPLGERTENSATVINRLRVKLANEPGANLFMMPVQDIRVGGRQANASYQFTLLADELNALRDWEPAIRKALGELPQLTDVNSDKEDKGAEMAITYDRDLMAQLGIDVREANSLLNNAFGQRQVSTIYEAMNQYKVVMEVAPEYTQDVSALDNMFVINKTGQAIPLSYFARWQPANAPLSVNHQGLSAASTIGFNVADGYTLNDAMVEIEKTMTALGVPSTVRGTFAGTAQVFQETLKSQLFLILAAIVTVYLVLGILYESYIHPLTILSTLPSAGVGALLALELFDTPFSLIALIGIMLLIGIVKKNAIIMVDFAIQAQRTAGLTAREAIFRASLLRFRPILMTTLAAIFGALPLMLGSGDGAELRQPLGITIVGGLIMSQLLTLFTTPVVYLSFDSLRQRWASRKAASRLKMEKHHEA

>*Providencia_vermicola*_DSM_17385_ARO:3003801_Bcr2/3

VQQQRSSYLGLILILGLLSMLMPLAIDMYLPSFPTMMQYFNVDEGRIQMTLNSYIFGFAIGQLFYGPMADSIGRKPVILGGVIVFAIASAACAVTESIDSLIWLRFLHGFAAAAASVVINALMRDMFTRDEFSRSMSFVVLVMTIAPLLAPILGGELMRWFSWHAIFWSIAIAAVIAVILVSFFVRETLPVTKRQKFHIGTTLRQFATLFRAKQVLFYILASSFSFAGMFSFLNAGAFVYIDLNGVSPQHFGYYFGINIIFLFIMTTINSRYVRRFGAEKMLYFGIIIQFIMGIWLLGTTAFSLDFWTLVIGVAIYVSGIAMITSNAMAVILDNYPHIAGTVSSLAGTIRFSIGALVGTLLSLIPAKNAWPMVGSMVACVALSMLFVLLAKKAK

>*Providencia_vermicola*_DSM_17385_ARO:3003995__GyrA*

MRDSGSMSEIAREITPVNIEEELKSSYLDYAMSVIVGRALPDVRDGLKPVHRRVLFAMNVLGNDWNKPYKKSARIVGDVIGKYHPHGDSAVYETIVRLAQPFSMRYMLVDGQGNFGSVDGDSAAAMRYTEIRMAKIAHELLADLEKETVDFVPNYDGTEQIPEVMPTRIPNLLVNGSSGIAVGMATNIPPHNLGEVINGCLAYIEDEDISIEGLMEHIPGPDFPTAAIINGRRGIIDAYKTGRGKVYIRASAEVEVDEKSGRETIIVSEIPYQVNKARLIEKIAELVKEKRVEGISALRDESDKDGMRIVIEIKRDAVGEVVLNNLYSLTQLQVSFGINMVALHQGQPKILNLKDIIAAFVRHRREVVTRRTIFELRKARDRAHILEALAIALANIDPIIELIRKAPTPAEAKAGLIARSWDLGNVAAMLERAGDDAARPEWLEEQFGVHDGQYFLTEQQAQAILDLRLQKLTGLEHEKLLEEYRELLVQIEALLFILRSPERLMEVIREELEIIRDTYNDPRRTEITENTADINIEDLINQEDVVVTLSHQGYVKYQPLSDYEAQRRGGKGKSAARTKDEDFIERLLVANTHDTILCFSSRGRLYWMKVYQLPEASRGARGRPIVNLLPLEQDERITAILPVREYEEGYTVFMATASGTVKKTPLQDFSRPRSAGIIAVNLNEGDELIGVDLTNGSNEVMLFSAQGKVVRFSEDAVRAMGRTATGVRGIKLMDDDKVVSLIIPRGEGHILTVTENGYGKRTEEAEYPTKSRATQGVISIKVSERNGNVVGAIQVDDTDQIMMITDAGTLVRTRVSEVSIVGRNTQGVTLIRTAENEKVVGLQRVAETEDDDNADESTDENGADDANINDQE

>*Providencia_vermicola*_DSM_17385_ARO:3004580_KpnE

MIYWIFLVLAIVTEVIGTLSMKHASVSGDFTGMVVMYVMIATSYILLAIAVKKVALGVAYALWEGIGILFITTFSVMWFGETLSPMKIGGLVLLITGIGLIKSGTKKATVRGSAQKVKQATQHAVNAAKSSALVGREAKSEA

>*Providencia_vermicola*_DSM_17385_ARO:3004583_KpnF

MASHFEWWHAAFLILAVVLEIIANILLKMSNGFKRYWLGILSLLAVLGAFSSLAQAVKGIELSIAYALWGAFGIIATVAAGWILFNQRLNYKGWGGIVLLLLGMVLIKMS

>*Providencia_vermicola*_DSM_17385_ARO:3000216_AcrB

MLAGLLAIIQLPVSQYPTIAPPAVSISANYPGADAETVQNTVTQVIEQNMNGIDNLVYMSSSSDSSGSASITLTFEAGTDGDIAQVQVQNKLQLAMPLLPQEVQQQGISVDKSTSSFLMVAGFVSNDGSMGQYDIADYVGSNIKDPLSRVNGVGETQLFGTQYAMRIWLKPEQLVKYNMTTTDVINAIKVQNNQVAAGQLGGSPSIANQRLNVSIIAQTRLNNAEEFSNILLRVNQDGSQVRIKDVADVQLGAENYSTVARFNGKPAAGIGIKLATGANALDTSNNVRAALEEMKPFFPQGLEIVYPYDTTPFVKISINEVVKTLVEAIMLVVVVMYLFLQNIRATLIPTIAVPVVLLGTFAILSAFGYSINTLTMFAMVLAIGLLVDDAIVVVENVERVMQEEGLSPKEATKKSMGQIQGALVGIAMVLSAVFIPMAFFGGSTGAIYRQFSITIVSSMILSVLVAMILTPALCATMLKPIEKGSHGSQKGFFGWFNRTFEKQAHHYTDSVSRMLNGTGRYLVIYLILVAGMAFMFVRLPSSFLPAEDQGVFLSMVQLPPGSTQEQTQAVLDEVNNYYHTDEKDNVESVFTVGGFSFAGQGQNMGLAFVVLKNWSERKGDENHVDAIVQRANIAMSKKQEALIYAFNLPAIVELGTADGFDFELVDKGNLGHDKLMQARNQLLGLAAQHPEMLQGVRPNGQDDTSQYRIYIDQQKAQAQGVAISDINATLSSVFGGSYVNDFIDRGRVKKVYVQGDAESRMLPSDISNLYVRNNQGKMVPFSAFLDESKDPWKFGSPRLERYNGVPAMNIQGQAAPGQSTGDAMLLMEKLTTENLPEGIGYEWTGMSYQERLAGNQAPALYAISLIVVFLCLAALYESWSVPFSVMLVVPLGIIGALLFTSVRGLDNDVYFKVGLLTTIGLSAKNAILIVEFAKDLMEKEGKGLVEATLNAVKMRLRPILMTSLAFMLGVIPLVFSNGAGSASQNSVGTGVLGGMFAATSLAIFFVPVFFVVIRRRFSKKSEDIEHSHPPAAH

>*Providencia_vermicola*_DSM_17385_ARO:3000207_AcrA

VLPLALLVLSGGLALSGCNDEQQGGGERPAPEVGIVTLKAEPLTLKTELPGRTSAFRVAEVRPQVSGIILKRNYKEGSDVEAGTSLYQIDPAPFQATYDSAKAELAKAQANANIAALTVKRYKPLLGTNYISQQEYDQATSTYAQALAAVKAAEAAVETARINLNYTKVTAPISGRTGKSNVTEGALVSTGQATELMRVQQLDPIYVDVTQSSDDFLRLKNEIAQGAVQKESGQAPVSLINNNGQEYAQKGVLEFSDVTVDETTGSITMRAVFPNPNKELLPGMFVRAILEDGVVENAILVPQQGLSRTAQGTSQVMVVGAENKIEVRTVKAGQAVGNKWLIKDGLKAGDRVVVIGLQKIKPGIVVNPKEANLETQSIDNQAKPEEKSK

>*Providencia_vermicola*_DSM_17385_ARO:3003373_AcrR

MARKTKQQAEETRQEILDAAIKTFSERGVSATSLADIAKAAGVTRGAIYWHFKNKVDLFYQACEFGDNQIIQAEEYYRSKYTNDPLSILRELLVFILTDFIDNPKNRALMEIFFLKCELVGEMAELVDFKRANYVASQCRIVDNLQDCIDAGQLPADLDIECAAIMIRSLMSGLLENWLLQPENFNINQHTITLVETLLETLKCNPAIRLKSTQIK

>*Providencia_vermicola*_DSM_17385_ARO:3003049_RosB MEHSTPLITTIVGGLALAYLLGMIAQRLKISPLVGYLAAGVLAGPFTPGFVADAALAPELAEIGVILLMFGVGLHFSLKDLLAVKAIAIPGAIAQIAVATLLGLGLSMLFGWGIFTGIVFGLCLSTASTVVLLRALEERQLIESQRGQIAIGWLIVEDLAMVLTLVLLPAAAAIMNTDDASFSELALGLAWTIGKVVLFIFIMIVVGRKVIPWILSRTASTGSRELFTLAVLALALGIAYAAVAIFDASFALGAFFAGMVLNESELSHRAAQDTLPLRDAFAVLFFVSVGMLFDPMVLIQQPLGILAVLAIIIIGKSAAALVLVRMFGHSRRTALTISVSLAQIGEFAFILAGMGLTLGVMDKDAQNLVLAGAIVSIMLNPVLFSLLDKYLERTETIEEQLLEETLEEETQVPVDICGHAIIVGYGRVGGMLADKLRRREIPVVVVEDTRARFEELAENGFSAILGNGANKETISLARIECAKTLLLTIPNGYEAGEIVATAKEMNPDVTVIVRAHYDDEVSFIKERGADHIIIGEHEIAKSMATLMCNDVEEFGCSIDDFIDNDNKKVEGKNLDEYLKPSH

>*Providencia_vermicola*_DSM_17385_ARO:3003048_RosA

MSEQTTTPNNANPSKPVNKTGKTVFSILTAISVSHLLNDMIQSLILAIYPLLQSDFSLSFAQIGMITLTYQITASLLQPVIGYYTDKHPQPYSLPIGMSFTLTGLILLAMAETFPMILFAAALVGTGSSVFHPESSRVARMASGGRHGLAQSFFQVGGNLGASLGPLLAALIIEPYGKGNLGWFSLAALLAIVILLQVSNWYKQQHRAASKKPIITGDIKVLPRKAVIGSLAVLLVLIFSKYFYLASISSYYTFYLIDKFGVSVQNAQIHLFVFLFAVAAGTMIGGPIGDKIGRKYVIWFSILGVAPFTLILPYASLYWTGVLTVIIGMILASAFSAILVYAQELIPGKTGMVSGLFFGLAFGMGGVGAAVLGHIADQTSIEQVYHYCAFLPLLGIFTVLLPNIGHK

>*Providencia_vermicola*_DSM_17385_ARO:3002523_AAC(2')-Ia

MLFVGFEGDFTRDDFEHALGGMHVLAYDQHRIVGHVAIIQRHMAINDKPISVGYVEAMAVLEKHRRQGIGRELMSMTNTIIGNCYQLGLLSASDEGFHLYQSLGWKVWKGTLFELCQGAYVRSEGEEGGVMGWSRDNSVEFTESLYCDFRGGDQW

>*Providencia_vermicola*_DSM_17385_ARO:3000535_MacB

MATLIELQGITRRYGEGENEVTVLKNVSLQINAGEMVAIIGASGSGKSTLMNIIGCLDKPSSGEYHIDGQNMALLDNDQLAELRREHFGFIFQRYHLLNHLSAEQNVEIPAVYAGSGKAERRERATQLLQRLGLGERVNYRPSQLSGGQQQRVSIARALMNGGQVILADEPTGALDSHSGEEVMKILKDLCAQGHTVIIVTHDPSVAQQAERIVEIKDGEIIRDSGSKNSKLAKLLSKAPTRKLSLGQVYGRFSEALLMAWRAMVVNKMRTLLTMLGIIIGIASVVSIMVIGDAAQGMVLNDIKSIGTNTISIYPGTDFGSDDAQNRQSLKATDIDALAKQPYVHAISGQLNGSARLRKGNLDASAQLTGIGRDYFNVYASKFSEGMNFTQDMADRRAQVVIIDENTKRRFFPKQANVVGETLLIGNMPATVIGVLEDQKSVFGSSKSLSVWLPDTTMNSKILNRPYYDSITVRVKDGYDAKNVEQQLTRLLTLRHGKKDVFTYNLDTFVKTAEKTTQTMQLFLTLVAVISLVVGGIGVMNIMLVSVTERTREIGIRMAVGARTSDVMQQFLIEAVLVCLMGGLMGIALSYGISLLAQMALPGWTFAFDPVALISAFVCSTAIGVIFGFLPARSAARLNPIDALARE

>*Providencia_vermicola*_DSM_17385_ARO:3000533_MacA

MKVKPKKKWPIYLVILIVAVGAAVFLFKGEKEEVFQTVDVTRGDLDKQVLATGKLDAVRKVDVGAQVSGQLQTLYVKEGDYVKKGDLLAVIDPKKAQNDVTESEATTRELEANLSLAQAELKLAQLTYKRHLSLAKLQAVAQQELDKARTDVDIKKAQVATYQAQIKRNQATLDTAKTNLKYTQITAPMDGIVTFIKTLQGQTVIAAQEAPTILTLADLDTMLVKAEVSEADVIYLEPGQEASFTVLGAPDKKFSGQLKDILPTPEKINDAIFYYARFEVPNPQHLLKLQMTAQVKIELDSRKDVLILPLSALGDEVSPQVYDVEVLEGQKAEKKQIEIGSRNDVSVEVIKGLNEGDKVIIGHSGSES

>*Providencia_vermicola*_DSM_17385_ARO:3004588_KpnG

MSSPEEMQQTQAPQRNKKRQRKNALMFLTFIFIAIGVGWGVYWYLVLRHYESTDNAYVAGNQVQIQSQVSGSVMTVNVDNTDFVQSGTVLVELDPRDAELALDKAKTELANSVRQTRQHIINSRQLQANIDVKRSELGRLQNDLKRREVLGSSNLIGKEELQHARESVVSAKAALDMAVEQYNANQAIVLNSPIEKQPAVEQAATQVRNAWLALQRTKIVSPVDGYVSRRSVQIGSQITPSTPLMAIVPSSGMWIDANFKETQLADMRIGQPAKVTADFYGKDVVFNGTVVGLDMGTGSAFSLLPAQNASGNWIKVVQRLPVRIALDPAQLEERPLRIGLSTEVTIDTLNKDGKVLSKGMRDAPAYHTSALAVDMSPADKIVTEIINNNAGNK

>*Providencia_vermicola*_DSM_17385_ARO:3004597_KpnH

MTAPLTGSKLAWMTIALSLATFMQVLDSTIANVAIPTIAGNLGASNSQGTWVITSFGVANAISIPVTGWLARRIGEVRLFLWSTGLFALTSWLCGISGSLEMLILFRVLQGLVAGPLIPLSQSLLLNNYPPAKRSMALALWSMTIVVAPICGPILGGYISDNYHWGWIFFINVPFSVAIIFAIMRTLKGRETKISIQRIDTIGLVLLVVGIGALQIMLDQGKELDWFNSTEIIVLTVIAVVAIAFLIVWELTDDHPVIDLSLFKERNFTIGCLSLSLAYMLYFGTIVLLPQLLQEVYGYTATWAGLASAPVGLLPLLITPIIGKFGNRIDMRYLVTFSFIIYAVCYYWRAYTFEPGMGFAAAAWPQFVQGLAIACFFMPLTTITLSGLPPEKMASASSLSNFTRTLAGAIGTSITTTMWTQRESMHHENLAEFVNPYNPNAQHMYSELAQIGMNEQQSAAYLAKTITDQGLILSANEIFWLSAGIFILLMVIVWFAKPPFGAGSKDGGGAH

>*Providencia_vermicola*_DSM_17385_ARO:3000027_EmrA

MSQSTHSETNNQKTKNEKRRTIWMTIATIIIILLFVAYGVYWFLVLRFQEYTDDAYVSGLQIPIIAQTTGNVTQVNFENTDLVKAGDVLVVLDKTNAQLAFEQAKHDLATTVRKTKELYINGDEYQAQIQKNRISLAQAQKDYQRRAALGRSGTISKEDLQHSQEAVQLAQAALDISIQQYNANRALLRNTALRKQPAIQQAADSVRSAWINLQRTEIKSPMTGYVSRRNVQVGSQVSPQSSLMAIVPVQPVWVDANFKETQLEKVRIGQPVTLNSDFYGDDIVYNGTVVGLDMGTGSAFSLLPAQNATGNWIKVVQRLPVRVELDPEQVAKHPLRIGLSMNVTIDIKDQNGPVLAEVQRTTPAFESDVLVLKLADVDHIIDGIISDNAD

>*Providencia_vermicola*_DSM_17385_ARO:3000074_EmrB

VIVADIQPLKGAKLVWATIALSLATFMQVLDSTIANVAIPTIAGNLGVSVSQGTWVITSFGVSNAISIAISGYLAKRFGEIRVFLWATALFTLFSLLCGFSDSLGMLILYRVLQGAVAGPVIPLSQSLIMRCYPPKMQNMALAFWSMTIILAPVFGPIFGGYISDNFHWGWIFFMNVPLGIFVIIVGSIILKGMESTVVKVPFNVIGLALLSVGVGCLQVLLDKGKELGWLASNEIVILAVVSAIALVFLVIWELTDKNPIVELSLFKSRNFTIGTISVSLAYMAYFGAIVLLPQLLQEVYGYTATWAGLALAPIGLLPVLFSAPVAKLSDFLDIRWIVTFSFVFYAICFFWRAYTFEPSMGFSAVVWPQLVQGMAVACFFMPLTTLTLSGLPPEKLASASSLANFFRTLAGSIGTSITTTMWSDRESVHHSQLTEFITDYNPESLAMYQGMAAHGLSTEQTSGFIAQQITSQGLIIAANEIFWLCGWVFIALIITVWFAKPPFGNNAK

>*Providencia_vermicola*_DSM_17385_ARO:3002494_SRT-2

MRNLFRQGRIFIALSLAFTAISANALNQQDVDSIIKPLMKQQGIPGMSVAISMEGKRYIYHYGVQSKQTQEPVNNNTLFEIGSLSKTFTATLAAYAQVQGKLDFSQTVSHYLPELKGSAFDHISVMNLATHTSGLSLFVPETITNSMELTRYYQNWIPEKAIGQFRSYSNLGVGLLGIVAAKQLKMPFEQAMEKLMLPSLGLKHTYIHVPKNQQKNYAQGYNKKDQPVRVTPQILDAEAYGLKSNAKDLIRFLEINMQTVKVAKSWQEAVEDTHTGLYLTDSFVQDMMWESYPWPVSLSQLQQGNRDEMALQPQKVEAIKPAMPPETRAFYNKTGSTNGFATYAVFIPEEQVAVVILANKWYPIPDRINAAYQLIEKIDNQN

>*Providencia_vermicola*_DSM_17385_ARO:3003665_NmcR

MSGNYRHRLPLNALRAFEASARHLSFTRAGLELNVTQAAVSQQVRLLEEQLGLELFIRLSRGLALTDEGLALLPVLSRSFDQIESLLLQFEDGHYHEVLSISVVGTFAVGWLLPRLSKFSDLYPYIDLRIMTHNNVVNLAAEGVDFAIRFGEGLWPLVENMPLFTTSHTVLCSEKVATGLKSPVDLKEQKLLRSYRKDEWEKWFLAAQIDPWRVKGPVFDSSRLMVEAAILTEGVALVPSCMFEHEISAGTLVQPFDIGVTLGGYWLSRLKSKPMTSAMMIFQQWILEEAQFS

>*Providencia_vermicola*_DSM_17385_ARO:3000518_CRP

VAVLIKDEEGKEMILSYLNQGDFIGELGLFEEGQERTAWVRAKIACEVAEISYKKFRQLIQVNPDILMRLSAQMASRLQTTSEKVGNLAFLDVTGRIAQTLLNLAKQPDAMTHPDGMQIKITRQEIGQIVGCSRETVGRILKMLEDQNLISAHGKTIVVYGTR

>*Providencia_vermicola*_DSM_17385_ARO:3003577_Ugd_(PmrE)

MLLSQHHEVVAVDIIPEKVAMLNNHQSPIVDTEIEQFLTEKTLNFRATEDKVDAYTGAQYVIVATPTDYDPKTNYFNTKSVESVIHDVNEINPTATIIVKSTIPVGFTARLREEFGYTNVIFSPEFLREGRALWDNLHPSRIVVGERSERAQIFADLLLEGAIKKDVPVLFTDGTEAEAIKLFANTYLAMRVAYFNELDTYAESRGLNARQIIEGVSLDPRIGSHYNNPSFGYGGYCLPKDTKQLLANYDDVPNNLINAIVESNRTRKDFISDAIIAKAPRKVGVYRLVMKAGSDNFRASAVQGIMKRIKAKGIEVVVYEPVMKETEFFRSKVVNDLAEFKAQCDIILANRMVPEIEDVADKVYTRDLFGND

>*Providencia_vermicola*_DSM_17385_ARO:3000220_GyrB*

MYIGDTDDGTGLHHMVFEVVDNAIDEALAGFCDDIVITIHADNSISVQDDGRGIPTGIHEEEGVSAAEVIMTVLHAGGKFDDNSYKVSGGLHGVGVSVVNALSEKLELVIKRDGKVHEQIYKHGEPQGPLSVVGETDQTGTRVRFWPSMDTFKGVTEFEYDVLAKRLRELSFLNSGVSIKLIDKRDGKEDHFHYEGGIKAFVEYLSRNKTPIHPSVFYFSTEKDGIGVEVSMQWNDGFQENVYCFTNNIPQRDGGTHLAGFRAAMTRTLNNYMEKEGYQKKSKVNATGDDAREGLIAVISVKVPDPKFSSQTKEKLVSSEVKTAVETMMNEKLVEYLLENPNDAKIVVGKIIDAARAREAARKAREMTRRKGALDLAGLPGKLADCQERDPALSELYLVEGDSAGGSAKQGRNRKNQAILPLKGKILNVEKARFDKMLSSQEVATLITALGCGIGRDEYNPDKLRYHSIIIMTDADVDGSHIRTLLLTFFYRQMPEIVERGHIFIAQPPLYKVKRGKQEQYIKDDDAMDDYLISIALDGAELHLSADAPAMKGEELEKLVVEYNAAHRIIRRLERLYPQALLNSLVYQSTLTEDDLKTKEKVEEWAKTLVQRLTDNEQFGSTYSYTIHENRERQLFEPTIRIRTHGVDTDYNLDFDFVHGSEYRRITHLGDIIGGLIEEGAYIQRGERRQDINNFEEALAWLTRESRRGLYVQRYKGLGEMNPEQLWETTMNPETRRMMRVTVKDAIATDLLFTTLMGDAVEPRRAFIEENALKAANIDI

>*Providencia_vermicola*_DSM_17385_ARO:3000808_MexI

MKFTDIFVRRPVLALVVSALIVLIGLFALSKLPIRQYPQLESATVTITTQYPGASAKLMQGFVTQPIAQAVSSVEGVDYLSSSSVQGSSLVTVRMELNRDSTQALAQVMAKVNQVRYKLPKEAYDPVIELSSGESTAVAYIGFSSTELSIPALTDYISRVVEPMYSSIEGVAKVQVFGGQQLAMRLWLDADKLAGRGLSAADVAQAVRQNNYQAAPGKVKGEYVISNVYVNTDLTNVDEFRDLVIRNDGNGLVRLKDVGTIELGAASTETSGLMNGEPAIYLGLFATPTGNPLVIVDGMNKLMPDIDKTLPPGVKVEMAFETSRFIKASIDQVINTLVEALLIVIAVIYLCLGSIRSVIIPILAIPLSMLGAAALMMAFGFSINLLTLLAMVLAIGLVVDDAIVVVENVHRHIEEGKSPVLAALIGAREVAGPVIAMTITLAAVYAPIGLMSGLTGALFKEFALTLAGAVIVSGIVALTLSPVMSSFMLNSKQNEGRMARMAETFFSTLAHYYTILLNFSLKNRWITGVIAVAVFISLPILYQSAPRELAPVEDQSSVLTAIKSPQHANLEYVERFSRKLHDVFMELPETESTWIINGTDGPSASFGGTNLSSWEQRDRPASAIQADLQGRVGDVEGNSIFVFQLPALPGSTGGLPIQMVLRSPQDYSVLYKTMEEIKQQARESGLFMVVDSDLDYNNPVVEVRINRSKANSLGIRMQDIGESLTLLVGENYINRFGMDGRSYDVIPQSMRNQRLTPEALSRHYVKAETGSMIPLSTVVDIHTQVEPNKLTQFNQQNAAIFQAIPAPGVTLGQAVAYLDEIANELPAGFSHDWQSDSRQYKQEGNTLAFAFMAALIIIYLVLAAQYESLVDPLIILITVPLSICGALIPLALGYATLNIYTQIGLVTLIGLISKHGILMVEFANELQANEGLDRRHAILKAAQIRLRPILMTTAAMVIGLVPLLFATGAGANSRFGLGLVIVTGMLVGTLFTLFVLPTIYSVLARNHSATALTPRRYELAEANRLIKETQETSQ

>*Providencia_vermicola*_DSM_17385_ARO:3000807_MexH

MSKKTIITLCTILIAAGGGSAIYSTYAQNEDGEKAAYQYPPTKVALAAVTLDTAPRTFYGVGELEAGSQVQVAAETNGRITKITFESGQQVKKGQVLVQLNDAVEQADLSRYQAQLRNAARLYQRTSSLSAQHLVAEAQVDSTRAERDIAQGLIRQTQALIAQKTIRVPFDGTIGIRQVHEGQYLTPGETIASLVDTKTLKLNFSLDEQASPELHQGQVVDVTVDAYPNKTFPARITAIDPLIGKSRTIALQATLENSDGTLKAGMYANVNVVRQANNQVLTIPETAVTYTAYGDTVFITEGEGDAMTVKRVSVKTGQRWDGKIEIEHGLSANDKVVTSGQLRLNNGSAVTPVAQDTLSEPTAHTQQGS

>*Providencia_vermicola*_DSM_17385_ARO:3000806_MexG

MPLVITKMLESNSLWFIARLLVLVLFISSGLAKVLNYESSLAEMRAAGLHPDWFFNIASAAVMLIGSVFVLLNRLLWLATGALATFLFLTIVIVHTFWSYTGEQAQIAMFWAIEHIAVIGGLIAIAIAGHFRGLYFSLKMQK

>*Providencia_vermicola*_DSM_17385_ARO:3003801_Bcr1

MVKSFDKHIPHWLIPLLGSLVAFGPLSIDMYLPALPQMGTALQATQGQMQYTLGAFFAGFCVGMLFYGPLSDLLGRRKMLLSGLAIFTVASLLCAQATNANTLIIFRALQAFGSGAAIVMARAIARDVYPANELPKVLSLMTLVTMIAPLLAPLLGGFLLIHFQWQAIFYLLALVGLVSVSTIFLLLPETLVHQRTSENLLCVAFKNYMQVLTDREALSIIGTMAFSFAGMFAFISGSPFVYINYFGVSEQHYGLLFGCNILGMIVMLLLNVKLLKIYSLTRILTMQSGFQLAFGLLLLLFYQQNLLIIVILVVLFLSMVNAIGTNSLSLLLQHRGKIAGSASALAISIQFALAALASVAVSVLQDESPFAMALVMAICAGLSFASQRLSAKNIRPQIQSVSSEKNEK

>*Providencia_vermicola*_DSM_17385_ARO:3005069_CsrA_(RsmA)

MLILTRRVGETLMIGDEVTVTVLGVKGNQVRIGVNAPKEVSVHREEIYQRIQAEKTQPDNQ

>*Providencia_vermicola*_DSM_17385_ARO:3003369_EF-Tu*

MITGAAQMDGAILVVAATDGPMPQTREHILLGRQVGVPYIIVFLNKCDMVDDEELLELVEMEVRELLSQYDFPGDDTPVVRGSALKALEGNPEWEAKIVELAGYLDSYIPEPERAIDRPFLLPIEDVFSISGRGTVVTGRVERGIIKVGEEVEIVGIKDTVKTTCTGVEMFRKLLDEGRAGENVGVLLRGTKREEIERGQVLAKPGSIKPHTKFESEVYILSKDEGGRHTPFFKGYRPQFYFRTTDVTGTIELPEGVEMVMPGDNINMIVTLIHPIAMDDGLRFAIREGGRTVGAGVVAKIIA

>*Providencia_vermicola*_DSM_17385_ARO:300328_RpoB*

MVYSYTEKKRIRKDFGKRPQVLDIPYLLSIQLDSFQKFIEQDPDGQNGLEAAFRSVFPIQSYSGNAELQYVSYRLGEPVFDVKECQIRGVTYSAPLRVKLRLIVYEREAPEGTVKDIKEQEVYMGEIPLMTENGTFVINGTERVIVSQLHRSPGVFFDSDKGKTHSSGKVLYNARIIPYRGSWLDFEFDPKDNLFVRIDRRRKLPATIILRAMDYNTEEILNLFFEKTVFQIRDNKLMMTLVPERLRGETASFDIEANGKVYVEKGRRITARHIRQLEKEEVSNIEVPVEYIAGKVVAKDYIDESTGELICAANMELSLDMLARLSQSGHKTIETLFTNDLDHGAYISETVRVDPTNDRLSALVEIYRMMRPGEPPTREAAENLFENLFFSEDRYDLSAVGRMKFNRSLNRDEIEGSGILSEEDIIEVMRKLIDIRNGKGEVDDIDHLGNRRIRSVGEMAENQFRVGLVRVERAVKERLSLGDLDALMPQDMINAKPISAAVKEFFGSSQLSQFMDQNNPLSEITHKRRISALGPGGLTRERAGFEVRDVHPTHYGRVCPIETPEGPNIGLINSLSVYAQTNEYGFLETPYRLVRDGLVTDEIHYLSAIEEGNFIIAQANTVLGEDGSFIEELVTCRNKGESSLFSREQVEYMDVSTQQVVSVGASLIPFLEHDDANRALMGANMQRQAVPTLRADKPLVGTGMERAVAVDSGVTAVARRGGSVQYVDASRIVIKVNEDEMYPGEAGIDIYNLTKYTRSNQNTCISQMPCVSLGEPVERGDVLADGPSTDLGELALGQNMRVAFMPWNGYNFEDSILVSERVVQEDRFTTIHIQELSCVSRDTKLGPEEITADIPNVGEAALSKLDESGIVYIGAEVKGGDILVGKVTPKGETQLTPEEKLLRAIFGEKASDVKDSSLRVPNGVSGTVIDVQVFTRDGVEKDKRALEIEETQLRDAKKDLTEELRIFEAGLFARIRSVLVNGGIEAEKLDKLPRDRWLELSLADEEKQNQLEQLAEQYDELKSEFEKKLDAKRRKITQGDDLAPGVLKIVKVYLAVKRQIQPGDKMAGRHGNKGVISKINPVEDMPYDENGNPVDIVLNPLGVPSRMNIGQILETHLGMAAKGIGEKINAMLKQHEEVAKLREFIQKAYDLGDDPRQKVDLNTFSDEEVMRLAENLKKGMPIATPVFDGAKEKEIKELLKLGGLPTSGQITLFDGRTGERFERQVTVGYMYMLKLNHLVDDKMHARSTGSYSLVTQQPLGGKAQFGGQRFGEMEVWALEAYGAAYTLQEMLTVKSDDVNGRTKMYKNIVDGSHQMEPGMPESFNVLLKEIRSLGINIELEDE
