# Supplementary figures and images for: Genomic analysis of the nomenclatural type strain of the nematode-associated entomopathogenic bacterium Providencia vermicola
Source: BMC Genomics. 2021 Oct 2;22:708. doi: 10.1186/s12864-021-08027-w (PMC8487129; doi:10.1186/s12864-021-08027-w)

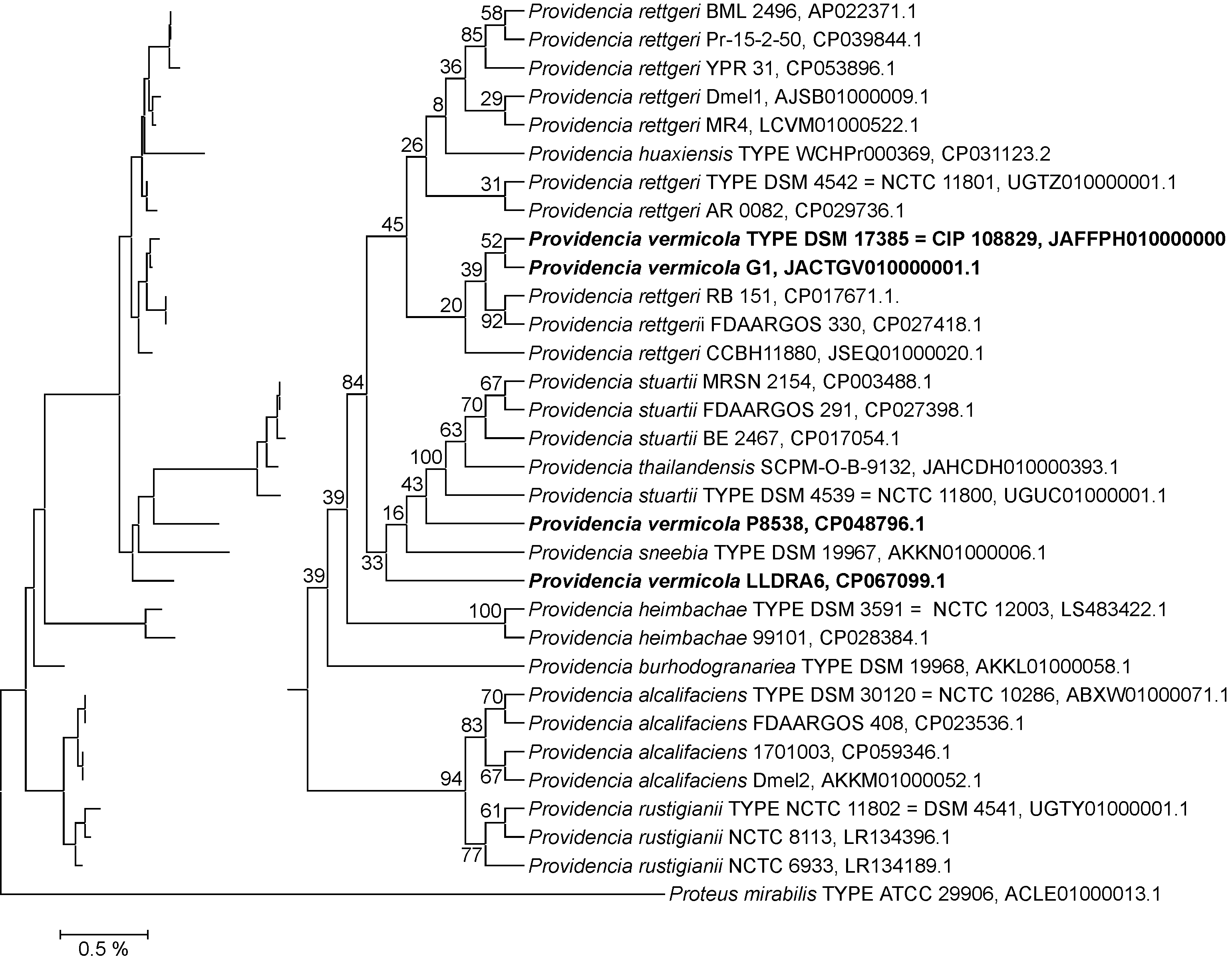

Supplement: Supplementary file 4 — Additional file 4: Suppl. Figure S1. Neighbor Joining (NJ) phylogeny of Providencia bacteria as reconstructed from complete 16S ribosomal RNA gene sequences. Terminal branches are labelled by genus, species and strain designations as well as GenBank accession numbers; “TYPE” indicates nomenclatural type strains of the respective taxonomic species. Bacterial strains that have been assigned to the species P. vermicola are in bold type. The phylogram representation of the tree has been expanded into a cladogram for better resolution and easier bootstrap support indication. Numbers on branches of the cladogram indicate bootstrap support percentages. The size bar corresponds to 0.5% sequence divergence along phylogram branches. The 16S rRNA encoding sequence from the closely related bacterium Proteus mirabilis has been used as outgroup. [file 12864_2021_8027_MOESM4_ESM.tif]

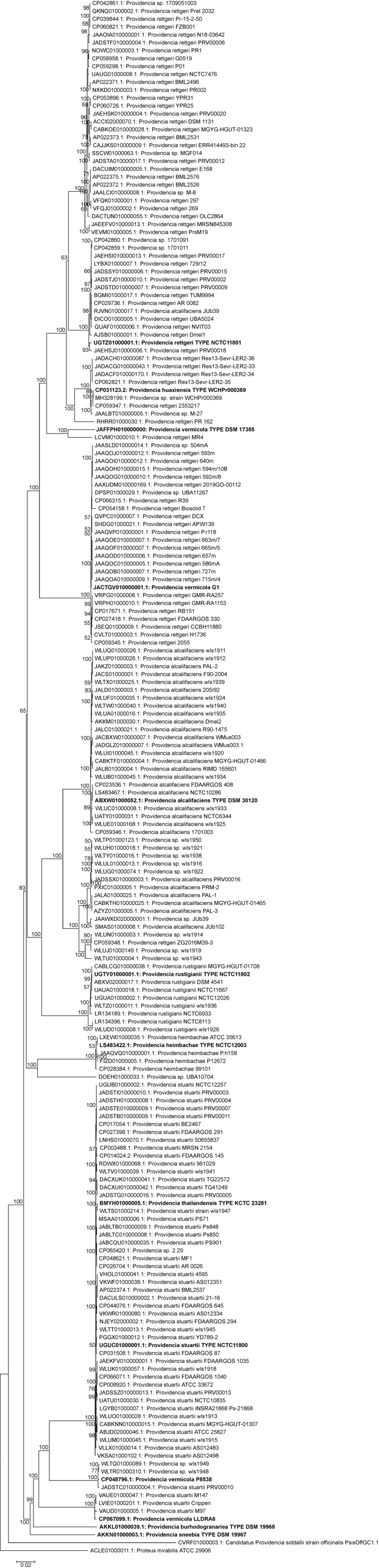

Supplement: Supplementary file 5 — Additional file 5: Suppl. Figure S2. Neighbor Joining (NJ) phylogeny of Providencia bacteria as reconstructed from concatenated complete fusA, gyrB, ileS, lepA and leuS gene sequences. Terminal branches are labelled by GenBank accession numbers followed by genus, species and strain designations. Providencia isolates provisionally assigned to the species P. vermicola and nomenclatural type strains representing the currently recognized Providencia species are presented in bold type; type strains are indicated by the word “TYPE” following the species designation. Numbers on branches indicate bootstrap support percentages superior to 50%. The size bar corresponds to 2% sequence divergence. The concatenation of orthologous sequences from the closely related bacterium Proteus mirabilis has been used as outgroup. [file 12864_2021_8027_MOESM5_ESM.tif]
